# Functionalisation of Lignin-Derived Diols for the Synthesis of Thermoplastic Polyurethanes and Polyester Resins

**DOI:** 10.3390/molecules30122604

**Published:** 2025-06-16

**Authors:** Rachele N. Carafa, Justin J. S. Kosalka, Brigida V. Fernandes, Unnati Desai, Daniel A. Foucher, Guerino G. Sacripante

**Affiliations:** Department of Chemistry and Biology, Toronto Metropolitan University, 350 Victoria Street, Toronto, ON M5B 2K3, Canada; rachele.carafa@torontomu.ca (R.N.C.); jkosalka@torontomu.ca (J.J.S.K.); brigida.fernandes@torontomu.ca (B.V.F.); unnati.desai@torontomu.ca (U.D.); daniel.foucher@torontomu.ca (D.A.F.)

**Keywords:** lignin, biobased, phenols, glycerol carbonate, ethylene carbonate, polyurethanes, polyesters

## Abstract

The functionalisation of lignin-derived phenolics (guaiacol, 4-propylguaiacol, eugenol, isoeugenol, phenol, *m*-cresol, catechol, syringol, syringaldehyde, and vanillin) for the synthesis of thermoplastic polyurethanes (PUs) and polyester (PE) resins is herein described. Diols were synthesised from phenolics in a one-step reaction using either glycerol carbonate or ethylene carbonate as a greener, solvent-free synthetic route. Nine of the diols were selected for the synthesis of Pus, and two of the diols were used for the synthesis of PE resins, with their physical and thermal properties characterised. Analysis of the PUs by differential scanning calorimetry (DSC) confirmed their amorphous nature, while thermogravimetric analysis (TGA) suggested improved thermal stability for all PUs with the addition of an alkyl or aldehyde substituent on the benzene ring regardless of the diisocyanate used. However, lower PU thermal stabilities were observed with the use of an aliphatic diisocyanate over an aromatic diisocyanate in the absence of an additional substituent. Analysis of the PEs by DSC also confirmed that the clear resins were all amorphous, and gel permeation chromatography (GPC) revealed significantly higher molecular weights and dispersities when an aliphatic diacid was utilised over an aromatic diacid.

## 1. Introduction

Approximately 70 million tonnes of lignin is discarded from the pulp and paper industry each year [1]. As a result, extensive efforts towards lignin valorisation to synthesise new materials has been undertaken [2]. Lignin, one of the main constituents of lignocellulose, is a complex 3-D amorphous macromolecule that is made of various phenylpropanoid units [3]. These units consist of monomeric phenolics, particularly *p*-hydroxyphenyl (H), guaiacyl (G), or syringyl (S) subunits, where their composition varies depending on the lignin source [4]. One common method of lignin valorisation is the depolymerisation of lignin into monomers, usually through methods such as pyrolysis. Due to the 3D network structure of lignin, the majority of the products from lignin pyrolysis are phenolics, such as guaiacol, 4-propylguaiacol, eugenol, catechol, phenol, and syringol, to name a few [5]. These phenolics can be further tailored to specialty polymers derived from biomass sources [6].

**Scheme 1:**
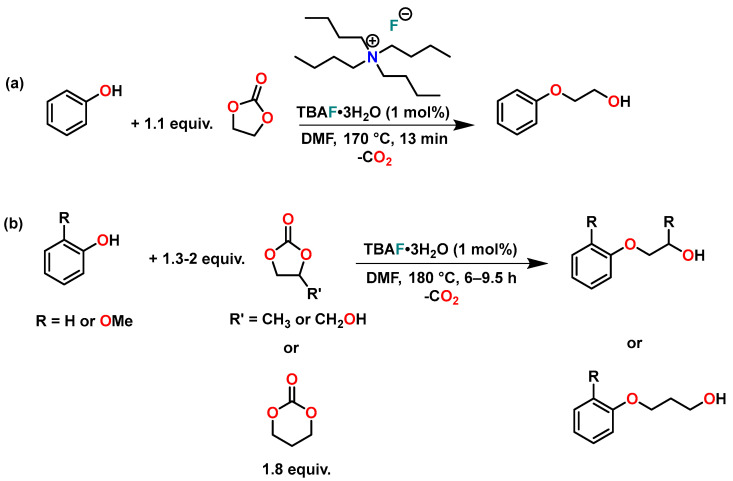
(**a**) Reaction of phenol with ethylene carbonate and TBAF•3H_2_O and (**b**) reaction of phenol or guaiacol with propylene carbonate, glycerol carbonate or trimethylene carbonate from Kao et al. [9].

Organic carbonates are of interest as greener candidates for alkylating agents, particularly replacing carcinogenic compounds like alkyl halides. They offer the advantage of being non-toxic, biodegradable, and good solvents due to their high boiling point [7]. Organic carbonates such as ethylene carbonate, propylene carbonate, and glycerol carbonate convert aromatic hydroxyl (OH) groups to hydroxy alkylated chains to increase compound reactivity for further functionalisation [8]. A study by Kao et al. utilised ethylene carbonate to synthesise a range of diols derived from phenolic compounds with the release of CO_2_ as a by-product [9]. They reacted their specific phenolics with 1.1 equivalents of ethylene carbonate, 1 mol% of tetrabutylammonium fluoride (TBAF•3H_2_O) at 170 °C under nitrogen (N_2_) and dimethyl formamide (DMF) as a solvent from anywhere between 13 min and 21 h to produce the hydroxy alkylated compounds in yields ranging from 66 to 98% (Scheme 1a). They also tested the scope of other cyclic carbonates, including propylene carbonate, glycerol carbonate, and trimethylene carbonate, on phenol or guaiacol to give the target compounds in moderate to high yields (Scheme 1b) [9].

Among these organic carbonates, glycerol carbonate is of interest since it can be derived from the reaction between glycerol and CO_2_ [10]. Glycerol is formed as a by-product in the formation of biodiesel and there are demands to repurpose captured CO_2_ from the atmosphere, making both of them available in high abundance [11,12]. Previous work in our group utilised a one-step reaction to form organic diols with glycerol carbonate acting as both the reagent and the solvent with either eugenol or vanillin [13]. Potassium carbonate (K_2_CO_3_) was used as a base, and the reaction was heated between 130 and 180 °C for anywhere between 5 and 24 h. While the eugenol-based diol was successfully synthesised, the vanillin-based diol was found to undergo a self-condensation polymerisation and was not investigated further for polymer production.

Polyester (PE) resins are known to have great low-temperature fixability, meaning they can easily be repaired at lower temperatures. They have been utilised as binder resins in toner production as they can allow for more controlled particle sizes and molecular weights [14]. Due the successful synthesis of the eugenol-based diol, Kosalka et al. selected it as the monomer of choice for PE co-block synthesis [13]. A PE block co-polymer was synthesised from eugenol using either succinic (SA), terephthalic (TA) or isophthalic acid (IA) as the diacid source under argon (Ar) for 48 to 72 h at high temperatures to be used for industrial scale toner production. It was found that only SA was successful at polymerising the eugenol diol, and the resulting polymer was found to have a high dispersity (*Ð*) that readily crosslinked upon further heating, making it unsuitable for applications in toners [13].

In addition to PEs, polyurethanes (PUs) are used in diverse markets such as packaging, automotive, construction, and electronics, to name a few [15]. All PUs can be classified as either thermoplastics or thermosets. Thermoplastic PUs can be synthesised from diols or polyols (such as hydroxyl terminated PEs) and diisocyanates with a functionality of two, forming compounds that not only have improved solubility but are also thermo-reprocessable, meaning they soften when heated while retaining their shape when cooled, allowing for them to be remelted and reused. Thermoset PUs are synthesised from the reaction between polyols and polyisocyanates with higher functionalities, which leads to crosslinked materials that are unable to be thermo-reprocessed or insoluble [16]. Thermoplastic PUs are also known to have high ductility and excellent biocompatibility, allowing them to be used in structures that require flexibility such as wearable devices [17], making them more desirable than thermoset PUs. Previous work in our group explored the synthesis of thermoplastic PUs from diols derived from 4-hydroxybenzaldehyde, vanillin or syringaldehyde [18]. The diols were synthesised in a two-step reaction from either a hydroxy alkylation with ethylene carbonate followed by a reduction with sodium borohydride (NaBH_4_), or a reduction with NaBH_4_ followed by Williamson–Ether synthesis with 1,3-dibromopropane. The PUs were then synthesised by reacting these diols with either methylene diphenyl diisocyanate (MDI) or pentamethylene diisocyanate (PDI) as the diisocyanate under N_2_ for 24 h at ambient temperatures, resulting in polymers with moderate to high molecular weights and good thermal stabilities [18]. However, abiding by the 12 principles of green chemistry [19], the two-step synthesis previously outlined results in diols with more derivatives produced, resulting in lower atom economy, while also requiring more energy for synthesis due to the multiple steps employed.

**Scheme 2:**
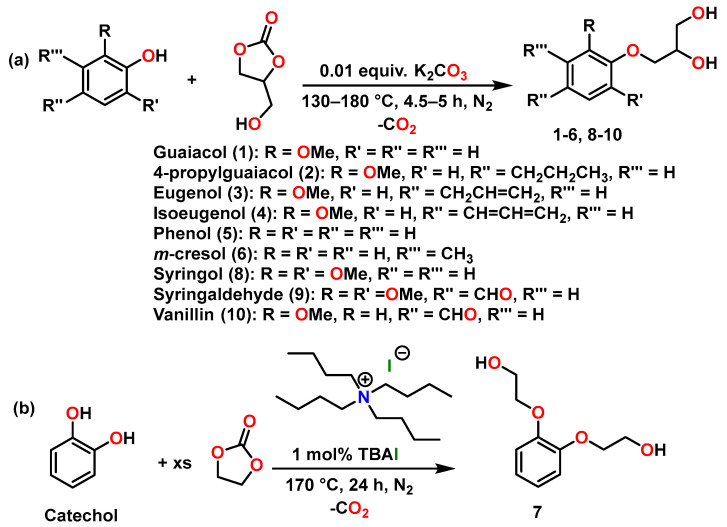
(**a**) Reaction of phenolics with glycerol carbonate. (**b**) Reaction of catechol with ethylene carbonate.

**Scheme 3:**
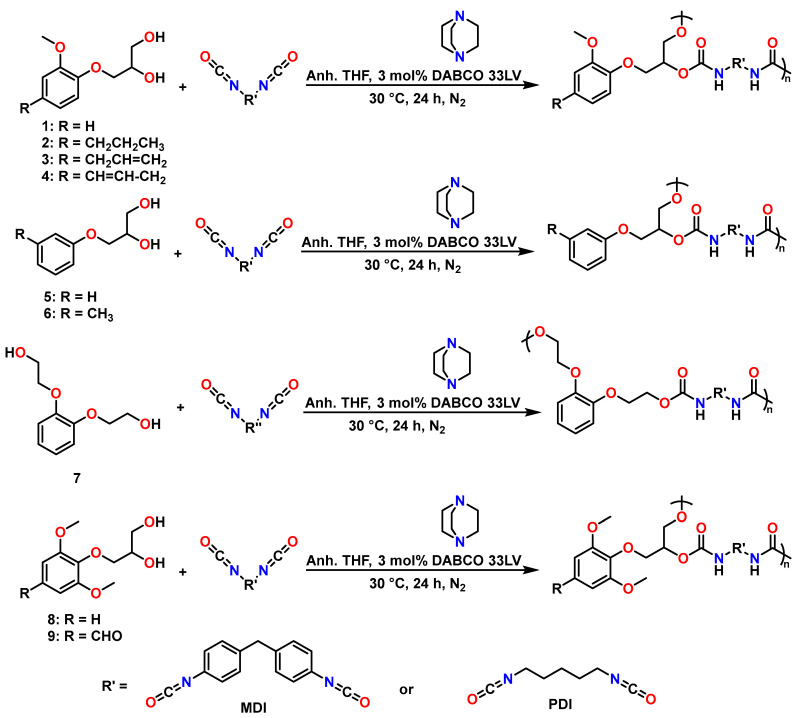
Reaction of lignin-based diols with diisocyanates to prepare PUs.

**Scheme 4:**
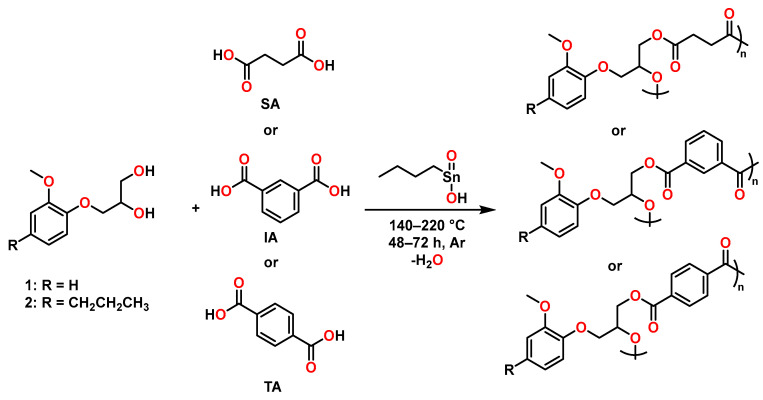
Reaction of lignin-based diols with diacids to yield PE resins.

The overall objective of this study is to synthesise various diols in a one-step solvent-free process from lignin-derived monomers using either glycerol carbonate or ethylene carbonate (Scheme 2) for the synthesis of PUs and PE resins for potential biobased applications. Lignin-based phenolics were reacted with glycerol carbonate, while catechol, which contains two phenolic OH groups, was reacted with ethylene carbonate to obtain the desired diols. These diols then underwent a one-step addition polymerisation with one of two diisocyanates (MDI or PDI) to synthesise PUs (Scheme 3). Select diols also underwent a one-step condensation polymerisation with one of three carboxylic acids (SA, IA or TA) to prepare high-molecular-weight PE resins (Scheme 4).

## 2. Results and Discussion

### 2.1. Lignin-Based Diol Synthesis

**Figure 1:**
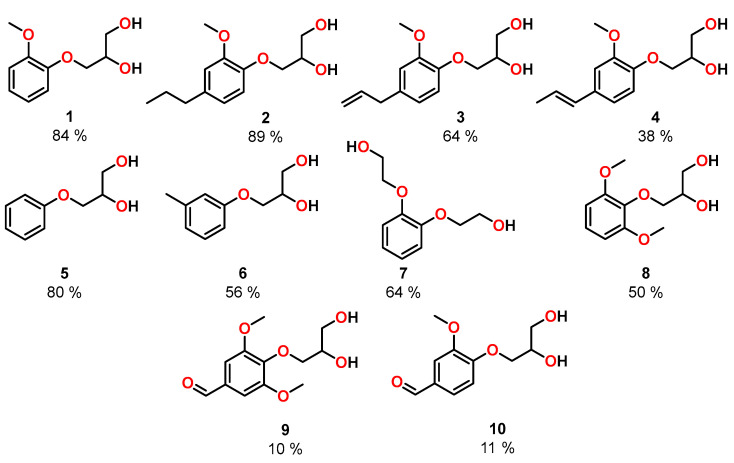
Structure and yields of diols **1**–**10**.

The synthesis of diols **1**–**6** and **8**–**10** followed a literature procedure from Kosalka et al. [13] (Scheme 2a). The respective phenol and glycerol carbonate were added in a 1:1 ratio, and K_2_CO_3_ was added in catalytic amounts as the base for the initial deprotonation step. The mixture was then refluxed over a temperature range of 130 to 180 °C for either 4.5 or 5 h under N_2_. Once cooled to room temperature, the diol initially formed as an oil and sometimes solidified to a waxy solid after leaving it to further dry. Purification was performed using either recrystallisation techniques or flash column chromatography to yield the diol as either a powder, an oil, or a glassy solid. The synthesis of diol **7** followed a modified procedure from Kao et al. [9] (Scheme 2b). Ethylene carbonate was added in a slight excess of catechol along with catalytic amounts of tetrabutylammonium iodide (TBAI) as a phase transfer catalyst in the absence of DMF. The reaction proceeded under N_2_ for 24 h at 170 °C, after which diol **7** was obtained after recrystallising it with diethyl ether (Et_2_O). The resulting diols and their respective yields are shown in Figure 1.

Analysis by ^1^H and ^13^C NMR for diols **1**–**3**, **5**–**8** and **10** matched previously reported literature data [9,11,13,20,21,22]. For diol **4**, analysis by ^1^H NMR revealed a multiplet at ~6.9 ppm for the aromatic protons, a doublet at ~6.3 ppm for the trans alkene proton, a doublet at ~6.1 ppm for the cis alkene proton, two multiplets at ~4.1 and ~3.7 ppm for the newly formed alkylated protons, a singlet at ~3.8 ppm for the methoxy protons, two broad singlets at ~2.3 and ~3.0 ppm for the aliphatic OH protons, and a doublet of doublets at ~1.9 ppm for the methyl protons (Figure S1). Confirmation of the protons interacting with each other was performed using 2D Correlation Spectroscopy (COSY) NMR, justifying the previously made ^1^H NMR assignments (Figure S2). Analysis by ^13^C NMR showed the ipso aromatic carbons at ~132, ~147 and ~150 ppm containing the alkene, ether, and methoxy substituents, respectively, with the vinyl carbons appeared at ~124 and ~130 ppm. The protonated aromatic carbon signals were revealed at ~109, ~115 and ~119 ppm. The newly synthesised alkylated carbons were observed at ~64, ~70 and ~72 ppm, similar to what was seen with diols **6**–**8**. The signals at ~56 and ~18 ppm represents the methoxy and methyl carbons, respectively (Figure S3). Further confirmation of the successful synthesis of diol **9** was observed by high resolution mass spectrometry (HRMS) using the direct analysis in real time (DART) method, where the calculated and found masses closely matched (Figure S4).

For diol **9**, the ^1^H NMR spectra revealed a singlet resonance at ~9.9 ppm for the aldehyde signal, a doublet at ~7.1 ppm for the aromatic protons, multiplets at ~3.8, ~4.0 and ~4.1 ppm for the newly formed alkylated protons, a doublet at ~3.9 ppm for the methoxy protons, and a broad singlet at ~2.7 ppm for the aliphatic OH protons (Figure S5). These assignments were confirmed by 2D COSY NMR (Figure S6). The ^13^C NMR spectra showed signals at ~191 ppm for the aldehyde carbon, the ipso aromatic signals at ~132, ~142, and ~153 ppm containing the aldehyde, ether, and methoxy substituents, respectively, the protonated aromatic carbons at ~107 ppm, the new alkylated carbons at ~63, ~71 and ~76 ppm, and the methoxy carbons at ~56 ppm (Figure S7). Analysis by DART-HRMS showed that the calculated and found masses closely matched, further confirming the successful synthesis of diol **9** (Figure S8).

The presence of unreacted starting material and, in some cases, a potential second product contributed to the lower yields of select diols, particularly those that required purification by column chromatography to isolate the target diol. This second product, which was observed by TLC, was suspected to be a disubstituted phenolic, which was previously proposed by Tabanelli et al. due to the intramolecular rearrangement of the glycerol carbonate moiety, particularly the carbonate group over the free OH group [7]. In addition, diol **10**, which was previously synthesised by Kosalka et al. [13], was found to undergo self-polymerisation when reacted for 12 h, whereas we found that the same diol remained stable after a 4.5 h reaction. The shorter time frame might allow for the aldehyde group to remain stable and not trigger polymerisation compared to longer times. Although the synthesis of diol **10** was successful, the yield was extremely low compared to the other diols apart from diol **9**, which was derived from syringaldehyde. In both cases, TLC analysis suggested that most of the product remained as unreacted starting material, which may be attributed to the aldehyde group. Since the reaction time cannot be increased at the risk of self-polymerisation, diol **10** was not investigated further for polymer synthesis.

### 2.2. PU Synthesis and Characterisation

Diols **1**–**9** underwent a polyaddition reaction to synthesise PUs with a diisocyanate, 1,4-diazabicyclo[2.2.2]octane (DABCO) 33LV as the catalyst, and anhydrous tetrahydrofuran (THF) as the solvent (Scheme 3) [18]. The diisocyanates selected were MDI and PDI, which were added in an equimolar amount of the diol, along with catalytic amounts of DABCO 33LV in dry THF. The reactions proceeded for 24 h under N_2_ after were then purified by precipitation into cold methanol (MeOH) for the MDI-containing PUs or hexanes for the PDI-containing PUs. After precipitation, the resulting PUs were either powders, clear gel-like materials, or gummy resins in low to high yields. The gel-like materials appear as a sticky substance that resembles a gel while the gummy resins collectively joined together in the shape of chewing gum during precipitation. The characterisation data is summarised in Table 1.

Analysis by ^1^H NMR for the MDI-containing PUs showed additional signals at ~7.1 and ~7.3 ppm for the aromatic MDI backbone as well as an additional signal at ~3.8 ppm for the methylene bridge in MDI (Figures S9, S13, S17, S20, S24, S28, S32, S36 and S40). For the PDI-containing PUs, ^1^H NMR spectroscopy showed additional signals between ~2.9 and ~3.1 ppm and ~1.2 to 1.4 ppm for the aliphatic PDI backbone (Figures S11, S15, S19, S22, S26, S30, S34, S38 and S42). However, due to the poor solubility of PU-3b, the ^1^H NMR spectrum could not be integrated, although the location of the signals confirmed its structure. Analysis by ^13^C NMR for the PUs did not always reveal all carbon signals due to poor solubility in dimethyl sulfoxide (DMSO). However, new signals were typically observed at ~120 and ~129 ppm for the aromatic MDI carbons (Figures S10, S14, S18, S21, S25, S29, S33, S37 and S41) and ~24 and ~30 ppm for the aliphatic PDI carbons (Figures S12, S16, S23, S27, S31, S35 and S39). Additionally, the poor solubility of PU-3b and PU-9b resulted in no ^13^C NMR spectra being obtained for either PU as both compounds swelled in DMSO-d_6_ when heated, although ^1^H NMR spectroscopy sufficiently confirmed the target structures. However, due to their overall poor solubility in organic solvents, GPC analysis could not be performed on these polymers.

Fourier transform infrared spectroscopy (FTIR) analysis confirmed the N-H stretch at ~3300 cm^−1^ and the C=O urethane stretches at ~1700 cm^−1^ for the MDI-containing PUs (Figure S43) and at ~1690 cm^−1^ for the PDI-containing PUs (Figure S44). C-H stretching was also observed for both sets of PUs at ~2800 and ~2900 cm^−1^, although the stretches were sharper in the PDI-containing PUs due to the aliphatic carbons present.

**Figure 2:**
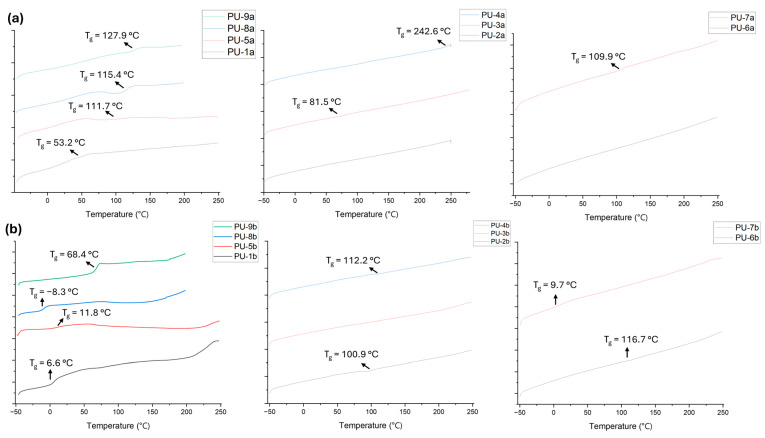
DSC traces of (**a**) PU-1a to PU-9a and (**b**) PU-1b to PU-9b (exotherm down).

Analysis by DSC revealed higher glass transition temperatures (T_g_) for the MDI-containing PUs than the PDI-containing PUs (Figure 2a,b) due to the rigidity of the aromatic rings in MDI compared to the flexibility of the aliphatic carbons in PDI. T_g_s were not observed for PU-2a, PU-6a and PU-3b, which may be due to them having very weak T_g_s that are not visible by DSC. While no noticeable trend was observed for the MDI containing PUs in terms of their T_g_, it was found that the PDI containing PUs derived from diols **1**, **5**, **7** and **8**, which all lacked a substituent, exhibited the lowest T_g_s compared to the remaining PDI PUs. This suggests that the addition of a substituent on the benzene ring improves the thermal properties of the PUs, particularly when the polymer exhibits a high degree of flexibility.

**Figure 3:**
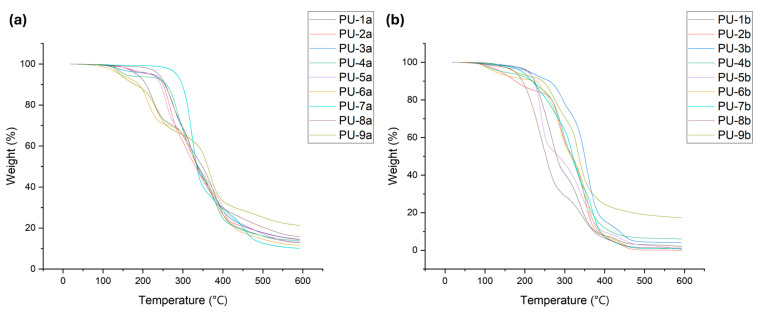
TGA curves of (**a**) PU-1a to PU-9a and (**b**) PU-1b to PU-9b.

Analysis by TGA on the thermal decomposition (T_d_) of these polymers revealed a similar trend. For the PUs synthesised from diols **1**, **5** and **8**, the MDI-containing polymers demonstrated significantly higher thermal stability at 50% weight loss compared to the PDI-containing polymers by ~50 to 80 °C (Figure 3). However, the addition of an alkyl, aldehyde or even an additional hydroxyalkyl substituent on the benzene ring showed similar thermal stabilities regardless of the diisocyanate used, suggesting that substituents improve the overall thermal stability of the PUs. This could lead to these particular monomers also being used in polyol formulations for PU foams to potentially improve their mechanical properties, which was also previously explored in our group [18]. The derivative weight changes for all PUs exhibited two to three decomposition temperatures (Figures S45 and S46), representing the breakdown of the aromatic rings and urethane linkages [18,23].

### 2.3. PE Synthesis and Characterisation

Diols **1** and **2** were selected for polycondensation reactions since they could be obtained in yields greater than 80% to compare against the PE resin previously synthesised from diol **3** in our group (Scheme 4) [13]. Carboxylic acids utilised in these reactions were SA, TA and IA. Diol **1** was successfully polymerised with SA (PE-1a), TA (PE-1b) and IA (PE-1c) to yield a yellow glassy solid. Purification was attempted using toluene and methanol mixtures; however, these procedures proved ineffective.

Analysis by ^1^H and ^13^C NMR confirmed the successful synthesis of all three PE resins. In addition to the signals corresponding to the polymeric aromatic and methoxy regions, signals unique to each carboxylic acid utilised were identified. For PE-1a, ^1^H NMR spectra showed a new singlet at ~2.7 ppm, representing the protons coming from SA (Figure S47), while ^13^C NMR spectra showed two new signals at ~171.8 ppm, representing the C=O groups from SA (Figure S48). For PE-1b, the TA aromatic protons were observed as a singlet at ~8.0 ppm (Figure S49) while new carbon signals were observed at ~165 ppm for the C=O resonance and ~129–133 ppm for the aromatic carbons of TA (Figure S50). For PE-1c, the IA protons appeared as multiplets at ~7.4, 8.2, and 8.7 ppm (Figure S51) and the IA carbons appeared at ~165 ppm for the C=O resonance and between 125–133 ppm for the aromatic carbons (Figure S52). There is also evidence of diol **1** being present in all three PE resins due to using an excess amount to achieve the desired acid value.

**Scheme 5:**
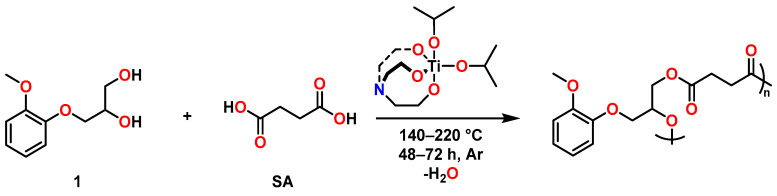
Reaction of **1** with SA and a titanium catalyst.

The reaction with SA was also repeated using a tetraalkyl titanate catalyst instead of a tin catalyst (Scheme 5). This was carried out to evaluate other options instead of using a tin-based catalyst as tin species are known to have detrimental environmental effects. ^1^H NMR analysis showed identical signals to that of PE-1a (Figure S53), suggesting a successful synthesis. Although the reaction was successful, titanium catalysts are susceptible to water, which is the by-product of this reaction. Therefore, if lignin-derived diols requiring higher and longer reaction times are employed, the tin catalyst will continue to be used as the titanium catalyst may degrade.

Diol **2** was also reacted with all three diacid species (Scheme 4), where SA was found to be the only diacid successfully yielding the desired PE resin. This is potentially due to the heating requirements being too intense when employing either TA or IA. This result was also previously observed when diol **3** was reacted with these same carboxylic acids, suggesting the alkyl substituent plays a role in the overall reaction mechanism [13]. The resulting polymer (PE-2) crosslinks before any useful material is generated, thereby making NMR analysis inconclusive. As this work was focused on producing bio-based materials suitable for toner production, PE resins tailored with specific thermal properties such as softening point (T_s_) and T_g_’s were desired. Table 2 summarises the properties of PE-1a, PE-1b, PE-1c and PE-2 in comparison to PE-3.

A patent by Shinji et al. specified that the ideal T_s_ for PE toners should be between 100 °C and 160 °C and the ideal T_g_ should be between 50 °C and 70 °C [24]. PE-1a, PE-1b and PE-1c, all polymerised from diol **1**, displayed similar T_s_’s slightly higher than 100 °C, whereas PE-2 was found to have a T_s_ less than 100 °C, which was also observed for PE-3. The DSC data showed significantly different results depending on the diacid source. Compounds PE-1b and PE-1c are similar in nature relative to PE-1a. The difference in T_g_ between either PE-1b or PE-1c and PE-1a is almost 30 °C, which can be attributed to the flexibility of the organic acids. PE-1a has flexibility due mainly to the ethyl chain, where it can bend and rotate, which is also why PE-2 and PE-3 exhibit similar T_g_’s, although PE-3 has a higher T_g_ by ~10 °C. PE-1b and PE-1c are much more rigid due to the aromatic species and as such, require higher energy inputs relative to PE-1a to initiate intramolecular rotation. The GPC data showed that PE-1a had a higher molecular weight (M_W_) and dispersity (*Ð*) compared to PE-1b and PE-1c, which may be due to the use of SA compared to IA or TA. The M_W_ and *Ð* of PE-2 were significantly higher than the PEs synthesised from diol **1**, although it was still not as high as PE-3. Unfortunately, PE-2 does not solidify and is gummy, limiting its applications. This high M_W_ may indicate that, as the polymer reaction conditions are intensified, the polymer chains grow to the point where they can no longer remain in the melt and precipitates. This may explain why the material solidifies after several days of growth. Due to its low T_g_ and liquid state at room temperature, PE-2 is not suitable for toner production. However, PE-1b and PE-1c show some promise in applications for toner production due to their relatively lower molecular weights and dispersities and their higher T_g_’s and T_s_’s that are within or close to the ideal range of toner properties specified by Shinji et al. [24].

## 3. Materials and Methods

### 3.1. Materials

Glycerol carbonate was supplied by Huntsman Corporation in Houston, TX, USA. Ethylene carbonate was purchased from Alfa Aesar in Ward Hill, MA, USA. MDI, PDI, and Fascat 4100 was supplied by ChemPoint in Bellevue, MA, USA. All other materials, reagents and catalysts were purchased from MilliporeSigma Canada in Oakville, ON, Canada. Anhydrous tetrahydrofuran (THF) was dried using an SPS solvent purification solvent. All other reagents and solvents were reagent grade and used as received. All reactions carried out in a N_2_ or Ar environment were performed using standard Schlenk line techniques.

#### 3.1.1. General Synthesis of Diols **1**–**6** and **8**–**10**

For exact reaction conditions, please refer to the Supplementary Materials. In a 50 mL round bottom flask, the phenol was added along with glycerol carbonate (1 equiv.) and K_2_CO_3_ (0.01 equiv.). The mixture was refluxed under N_2_ at 130 °C for 30 min, and the temperature was increased by 10 °C every 30 min until a temperature of 180 °C was reached. At 180 °C, the reaction proceeded for an additional 2–2.5 h. The material was extracted using H_2_O or brine and ethyl acetate (EtOAc) (~50 mL × 3), and the organic layer was dried using sodium sulfate (Na_2_SO_4_). The solvent was decanted and removed via rotary evaporation and the product was purified by either recrystallisation or flash column chromatography.

#### 3.1.2. Synthesis of Diol **7**

In a 100 mL 3 neck round bottom flask, catechol (1.101 g, 0.010 mol), ethylene carbonate (2.113 g, 0.024 mol), and TBAI (1 mol%, 0.037 g) were added together, and the reaction was heated at 170 °C for 24 h under N_2_. After cooling to RT, the material hardened into an orange amber solid. The product was then recrystallised using Et_2_O to yield a brown powder. Yield: 64% (1.260 g).

### 3.2. General Preparation of PUs

The diol (0.50–1.00 g) was added to a 100 mL 3-neck round bottom flask along with anhydrous THF (10–20 mL) and DABCO 33LV (3 mol% based on diol amount). MDI or PDI (1 equiv.) was added under N_2_, and the reaction stirred at 30 °C for 24 h, after which the mixture was precipitated or washed in cold MeOH (MDI) or hexanes (PDI) to remove any unreacted diol or diisocyanate. Yield: PU-1a: 94% (2.22 g); PU-1b: 75% (1.38 g); PU-2a: 33% (0.67 g); PU-2b: 27% (0.40 g); PU-3a: 52% (1.06 g); PU-3b: 44% (0.73 g); PU-4a: 94% (1.92 g); PU-4b: 61% (1.01 g); PU-5a: 58% (1.45 g); PU-5b: 66% (1.26 g); PU-6a: 12% (0.28 g); PU-6b: 17% (0.31 g; PU-7a: 98% (2.22 g); PU-7b: 33% (0.58 g); PU-8a: 83% (1.73 g); PU-8b: 30% (0.50 g); PU-9a: 92% (0.91 g); PU-9b: 57% (0.46 g).

### 3.3. General Preparation of PEs

#### 3.3.1. Preparation of PE-1a, PE-1b and PE-1c

In a 500 mL 3-neck flask, 1 (50.00 g, 0.25 mol) and SA (25.32 g, 0.21 mol) were added, resulting in a 1.15:1 ratio of diol to acid, along with Fascat 4100 (0.50 g, 0.002 mol). Independent experiments were also performed using TA (35.62 117 g, 0.21 mol) and IA (35.62 g, 0.21 mol) along with 1 (50.00 g, 0.25 mol). Polymerisations involving TA and IA followed an almost identical protocol. All polymerisations were carried out under an Ar blanket. Using a mechanical stirring rod, the reagents in the ratios mentioned above were heated to 140 °C resulting in a melt. The temperature was kept at 140 °C for 1 h. After the 1 h mark, the temperature was raised by 5 °C/h until a temperature of 180 °C was reached. The reaction then was left overnight to react (11–13 h). The reaction temperature was then increased from 180 °C to 185 °C and allowed to run for 1 h. The temperature was then increased 5 °C/h until 200 °C was reached and then allowed to react again overnight (14–16 h). The temperature was then increased by 5 °C/h until a temperature of 240 °C was reached. The reaction was then allowed to run for 14–30 h. Experiments involving TA and IA were left to run at 240 °C until the acid value was ~15 mg KOH/g. The resulting material was clear with a yellowish tint. Yield: PE-1a: 91% (70.75 g); PE-1b: 94% (81.07 g); PE-1c: 89% (76.76 g).

#### 3.3.2. Evaluation of the Catalyst for PE-1a

The polymerisation of 1 with SA was repeated in the presence of a tetraalkyl titanate catalyst, (titanium (IV) triethanolaminato isopropoxide (8.2 Ti wt%)) (0.50 g, 0.002 mol). The resulting material appeared identical to the material depicted above.

#### 3.3.3. Preparation of PE-2

In a 500 mL 3-neck flask, 2 (50.00 g, 0.21 mol) along with SA (20.89 g, 0.18 mol), resulting in a 1.15:1 ratio of diol to acid and Fascat 4100 (0.50 g, 0.002 mol). Polymerisations were carried out under an Ar blanket. Using a magnetic stirring rod, the reagents were heated to 140 °C resulting in a melt. The temperature was kept at 140 °C for 1 h, after which the temperature was raised by 5 °C/h until a temperature of 180 °C was reached. The reaction was left overnight to react (11–13 h). The colour of the material did not noticeably change. The reaction temperature was increased from 180 °C to 185 °C and allowed to run for 1 h. The temperature was then increased by 5 °C/h until 200 °C was reached. It was then allowed to react again overnight (14–16 h). The colour of the material had not changed after the second day of reacting. The temperature was then increased by 5 °C/h until a temperature of 220 °C was reached. The reaction was then allowed to run for 14–30 h. In all attempts, at some point after 48 h, the material in the melt precipitated out and was found to be insoluble in any solvent and incapable of melting. Prior to solidification, the resulting material was clear with a yellowish tint and was quite soft. Yield: 88% (57.47 g).

### 3.4. Characterisation Techniques

Flash column chromatography was carried out on silica gel (Silica Gel 60, 40–63 µm, EMD, MilliporeSigma, Oakville, ON, Canada). Reactions and chromatographic purifications were monitored by thin layer chromatography (TLC). Silica-coated aluminium plates (Alugram Sil G/UV_254_, Macherey-Nagel, Düren, Germany) were used for TLC tests. Plates were visualised with UV light. ^1^H, ^13^C and 2D NMR were carried out on a 400 MHz Bruker Avance II spectrometer (Billerica, MA, USA) using CDCl_3_ or DMSO-d_6_ as the solvent with the TOPSPIN software package. ^1^H spectra (400 MHz) and ^13^C spectra (101 MHz) were referenced against the internal standard tetramethylsilane (TMS). NMR data were reported as: chemical shift d (ppm) relative to the solvent, multiplicity, number of protons, and coupling constant *J* (Hz). Mass spectrometry data was obtained on a JEOL AccuTOF 4G (DART) mass spectrometer (Tokyo, Japan) at the Advanced Instrumentation for Molecular Structure Mass Spectrometry Laboratory at the University of Toronto with the JEOL msAxel software package. FTIR analysis was performed on an Agilent Cary 630 FTIR Spectrometer (Santa Clara, CA, USA) using the MicroLab PC software package. For the PU’s, DSC was completed on a DSC Q20 TA Instrument (New Castle, DE, USA) with a flow rate of 40 mL/min at a heating rate of 10 °C/min in an aluminium Tzero pan under a N_2_ atmosphere with ~10 mg of sample with the Universal Analysis software package. The third cycle was used for analysis of the sample. TGA was conducted on a PerkinElmer TGA 4000 (Waltham, MA, USA) under a N_2_ atmosphere with a flow rate of 40 mL/min and a heating rate of 10 °C/min in a Platinum HT pan with ~10 mg of sample with the TRIOS software package. For the PEs, DSC, GPC and TGA analysis were performed by the analytical staff at Xerox Research Centre of Canada (XRCC). Acid value measurements were performed by titration with a 0.1 N solution of potassium hydroxide in ethanol and 3–5 drops of phenolphthalein as an indicator. Samples were first dissolved in THF, and the volume of titrant used to cause a colour change was recorded to determine the acid value. Polymerisations were considered complete once the acid value of the material was between 15 and 20.

## 4. Conclusions

Ten diols were synthesised in a one-step reaction using either glycerol carbonate or ethylene carbonate and further reacted to synthesise PUs and PEs. While most of the diols were obtained in moderate to high yields, only the diols containing an aldehyde group exhibited yields lower than 20%. The synthesis of eighteen PUs was confirmed by NMR and FTIR while the synthesis of four PEs was confirmed by NMR. DSC analysis of the PUs and PEs was consistent with the degree of rigidity or flexibility of the diisocyanate or diacid used. TGA analysis of the PUs demonstrated lower thermal stabilities of the PDI-containing PUs compared to the MDI-containing PUs synthesised from diols **1**, **5** and **8**, which only contained methoxy or hydroxy alkylated substituents. Consistent thermal stabilities were observed for the remaining PUs with alkyl or aldehyde substituents regardless of the diisocyanate used, offering their respective starting monomers more promising applications in PU foams. GPC analysis of the PEs showed the highest molecular weights and dispersities when SA was used as a diacid, and the T_s_ values were relatively similar to each other. These results demonstrated the most potential for PE-1b and PE-1c, both derived from diol **1** and aromatic diacids TA or IA, as better candidates for PE-based toners.

## Figures and Tables

**Table 1:** Characterisation data of PUs.

Sample	Diisocyanate	Appearance	Yield (%)	T_g_ (°C)	T_d_ (5%) (°C)	T_d_ (50%) (°C)
PU-1a	MDI	Gummy	94	53.2	185	348
PU-1b	PDI	Gel	75	6.6	209	279
PU-2a	MDI	Powder	33	-	215	331
PU-2b	PDI	Gel	27	100.9	140	319
PU-3a	MDI	Gummy	52	81.5	224	335
PU-3b	PDI	Powder	44	-	212	350
PU-4a	MDI	Powder	94	242.6	155	338
PU-4b	PDI	Gummy	61	112.2	145	316
PU-5a	MDI	Gummy	58	111.7	222	339
PU-5b	PDI	Gummy	66	11.8	206	285
PU-6a	MDI	Gummy	12	-	142	336
PU-6b	PDI	Gel	17	116.7	124	317
PU-7a	MDI	Powder	98	109.9	284	335
PU-7b	PDI	Gel	33	9.7	193	320
PU-8a	MDI	Gummy	83	115.4	239	337
PU-8b	PDI	Gel	30	−8.3	175	253
PU-9a	MDI	Gummy	92	127.9	143	362
PU-9b	PDI	Powder	57	68.4	176	335

**Table 2:** Characterisation data of PE resins.

Sample	Diacid	Yield (%)	T_g_ (°C)	T_s_ (°C)	M_w_ (g/mol)	M_n_ (g/mol)	*Ð* (M_w_/M_n_)
PE-1a	SA	91	17.7	100.1	26,440	3730	7.1
PE-1b	TA	94	41.8	102.9	3900	2790	1.4
PE-1c	IA	89	49.5	104.1	5070	2530	2.0
PE-2	SA	88	20.1	<100	94,500	2840	33.3
PE-3 [13]	SA	90	29.9	<100	132,900	1850	71.9

## Data Availability

The original contributions presented in this study are included in the article/Supplementary Materials. Further inquiries can be directed to the corresponding author.

## Supplementary Materials

The following supporting information can be downloaded at: https://www.mdpi.com/article/10.3390/molecules30122604/s1, Complete synthesis and characterisation details of diols **1**–**10**; Figures S1–S4: ^1^H NMR, 2D COSY NMR, ^13^C NMR spectra and HRMS (DART) analysis of diol **4**; Figures S5–S8: ^1^H NMR, 2D COSY NMR, ^13^C NMR spectra and HRMS (DART) analysis of diol **9**; Figures S9–S18: ^1^H and ^13^C NMR spectra of PU-1a, PU-1b, PU-2a, PU-2b and PU-3a; Figure S19: ^1^H NMR spectra of PU-3b; Figures S20–S41: ^1^H and ^13^C NMR spectra of PU-4a, PU-4b, PU-5a, PU-5b, PU-6a, PU-6b, PU-7a, PU-7b, PU-8a, PU-8b and PU-9a; Figure S42: ^1^H NMR spectra of PU-9b; Figure S43: FTIR analysis of PU-1a to PU-9a; Figure S44: FTIR analysis of PU-1b to PU-9b; Figure S45: Derivative weight change of PU-1a to PU-9a; Figure S46: Derivative weight change of PU-1b to PU-9b; Figures S47–S52: ^1^H and ^13^C NMR of PE-1a, PE-1b and PE-1c; Figure S53: ^1^H NMR spectra of PE-1a using titanium catalyst.

## Abbreviations

The following abbreviations are used in this manuscript:

Ar	Argon
CDCl_3_	Deuterated chloroform
COSY	Correlation spectroscopy
*Ð*	Dispersity
DABCO	1,4-diazabicyclo[2.2.2]octane
DART	Direct analysis in real time
DMF	Dimethylformamide
DMSO	Dimethyl sulfoxide
DMSO-d_6_	Deuterated dimethyl sulfoxide
DSC	Differential scanning calorimetry
Et_2_O	Diethyl ether
EtOAc	Ethyl acetate
FTIR	Fourier transform infrared spectroscopy
GPC	Gel permeation chromatography
HRMS	High resolution mass spectrometry
IA	Isophthalic acid
K_2_CO_3_	Potassium carbonate
MeOH	Methanol
MDI	Methylene diphenyl diisocyanate
Mn	Number average molecular weight
MW	Weight average molecular weight
N_2_	Nitrogen
NaBH4	Sodium borohydride
Na_2_SO_4_	Sodium sulfate
NMR	Nuclear magnetic resonance
PDI	Pentamethylene diisocyanate
PE	Polyester
PU	Polyurethane
SA	Succinic acid
TA	Terephthalic acid
TBAI	Tetrabutylammonium iodide
TBAF•3H_2_O	Tetrabutylammonium fluoride
TGA	Thermogravimetric analysis
Tg	Glass transition temperature
THF	Tetrahydrofuran
TLC	Thin layer chromatography
Ts	Softening point
