# Supplementary material for: Functionalisation of Lignin-Derived Diols for the Synthesis of Thermoplastic Polyurethanes and Polyester Resins
Source: Molecules. 2025 Jun 16;30(12):2604. doi: 10.3390/molecules30122604 (PMC12195812; doi:10.3390/molecules30122604)
Supplement: Supplementary file 1 [file molecules-30-02604-s001.zip › molecules-3642863-supplementary.pdf]

Supporting Information

Contents

Diol Synthesis 1
Synthesis of Diol **1** 1
Synthesis of Diol **2** 2
Synthesis of Diol **3** 3
Synthesis of Diol **4** 4
Synthesis of Diol **5** 5
Synthesis of Diol **6** 6
Characterization of Diol **7** 7
Synthesis of Diol **8** 8
Synthesis of Diol **9** 9
Synthesis of Diol **10** 10
NMR and Mass Spectra of Diols 4 and 9 12
Polyurethane NMR Data 20
Polyurethane FTIR Data 54
Polyurethane TGA Data 56
Polyester NMRs 58

Diol Synthesis

Synthesis of Diol **1**

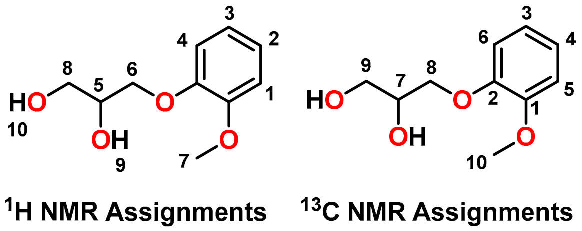

Guaiacol (5.000 g, 0.040 mol), glycerol carbonate (4.869 g, 0.041 mol), and K_2CO_3 (0.025 g, 0.0002 mol) were added to a 50 mL round bottom flask. The mixture was refluxed under

N_2 at 130 °C for 30 min, and the temperature was increased by 10 °C every 30 min until a temperature of 180 °C was reached. At 180 °C, the reaction proceeded for an additional 2.5 h. The material was extracted using H_2O and EtOAc (~50 mL x 3), and the organic layer was dried using Na_2SO_4. The solvent was decanted and removed *via* rotary evaporation to form a yellow oil. Recrystallization was performed using Et_2O to yield a white solid. Yield: 84 % (6.225 g). Agrees with literature.[1]

^{1}H NMR ($CDCl_3$, 400 MHz): δ = 6.88-6.97 (m, 4H, H1, H2, H3 and H4), 4.12-4.17 (m, 1H, H5), 4.03-4.07 (m, 2H, H6), 3.85 (s, 3H, H7), 3.75-3.84 (m, 2H, H8), 2.86 (br s, 2H, H9 and H10) ppm

^{13}C{^{1}H} NMR ($CDCl_3$, 101 MHz): δ = 149.70 (s, C1), 147.97 (s, C2), 122.26 (s, C3), 121.11 (s, C4), 114.88 (s, C5), 111.83 (s, C6), 72.29 (s, C7), 70.01 (s, C8), 63.88 (s, C9), 55.82 (s, C10) ppm

Synthesis of Diol **2**

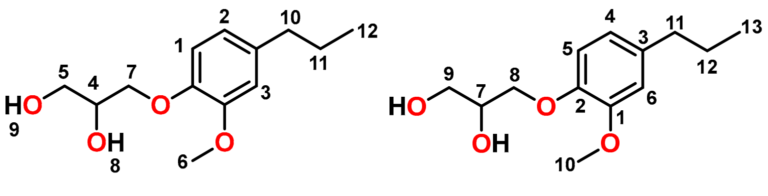

^{1}H NMR Assignments ^{13}C NMR Assignments

4-propylguaiacol (5.000 g, 0.030 mol), glycerol carbonate (3.649 g, 0.031 mol), and K_2CO_3 (0.025 g, 0.0002 mol) were added to a 50 mL round bottom flask. The mixture was refluxed under N_2 at 130 °C for 30 min, and the temperature was increased by 10 °C

every 30 min until a temperature of 180 °C was reached. At 180 °C, the reaction proceeded for an additional 2.5 h. The material was extracted using H_2O and EtOAc (50 mL x 3), and the organic layer was dried using Na_2SO_4. The solvent was decanted and removed *via* rotary evaporation to form a pale-yellow oil. Recrystallization was performed using Et_2O to yield a white solid. Yield: 89 % (6.430 g). Agrees with literature.[2]

^{1}H NMR ($CDCl_3$, 400 MHz): δ = 6.83-6.85 (d, 1H, H1, $^3J_{HH}$ = 8.6 Hz), 6.70-6.71 (m, 2H, H2 and H3), 4.13-4.14 (q, 1H, H4, $^3J_{HH}$ = 6.4 Hz), 4.03-4.05 (m, 2H, H5), 3.85 (s, 3H, H6), 3.77-3.81 (m, 2H, H7), 2.74 (br s, 2H, H8 and H9), 2.51-2.55 (t, 2H, H10, $^3J_{HH}$ = 7.5 Hz), 1.59-1.65 (sxt, 2H, H11, $^3J_{HH}$ = 7.4 Hz), 0.92-0.95 (t, 3H, H12, $^3J_{HH}$ = 7.3 Hz) ppm

^{13}C{^{1}H} NMR ($CDCl_3$, 101 MHz): δ = 149.54 (s, C1), 145.87 (s, C2), 137.19 (s, C3), 120.59 (s, C4), 115.22 (s, C5), 112.16 (s, C6), 72.85 (s, C7), 69.96 (s, C8), 63.92 (s, C9), 55.77 (s, C10), 37.76 (s, C11), 24.73 (s, C12), 13.83 (s, C13) ppm

Synthesis of Diol **3**

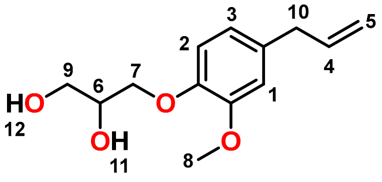

^{1}H NMR Assignments

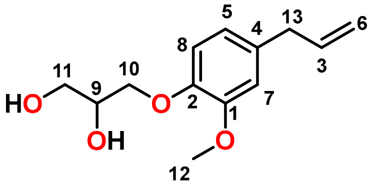

^{13}C NMR Assignments

Eugenol (5.000 g, 0.030 mol), glycerol carbonate (3.705 g, 0.031 mol), and K_2CO_3 (0.025 g, 0.0002 mol) were added to a 50 mL round bottom flask. The mixture was refluxed under N_2 at 130 °C for 30 min, and the temperature was increased by 10 °C every 30 min until

a temperature of 180 °C was reached. At 180 °C, the reaction proceeded for an additional 2 h. The material was extracted using H_2O and EtOAc (50 mL x 3), and the organic layer was dried using Na_2SO_4. The solvent was decanted and removed using rotary evaporation and high vac to form an orange oil. The material was left exposed to air, where it solidified after 2 days into an orange waxy solid. Recrystallization was performed using a 1:1 EtOAc:hexanes solution to yield a white powder. Yield: 64 % (4.603 g). Agrees with literature.[3]

^{1}H NMR ($CDCl_3$, 400 MHz): δ = 6.85-6.87 (d, 1H, H1, $^4J_{HH}$ = 8.6 Hz), 6.71-6.73 (m, 2H, H2, H3), 5.90-5.98 (m, 1H, H4), 5.05-5.10 (m, 2H, H5), 4.13-4.14 (m, 1H, H6), 4.03-4.05 (m, 2H, H7), 3.85 (s, 3H, H8), 3.79 (m, 2H, H9), 3.33-3.35 (d, 1H, H10, $^3J_{HH}$ = 6.6 Hz), 3.27 (br s, 1H, H11), 2.52 (br s, 1H, H12) ppm

^{13}C{^{1}H} NMR ($CDCl_3$, 101 MHz): δ = 149.71 (s, C1), 146.27 (s, C2), 137.45 (s, C3), 134.40 (s, C4), 120.78 (s, C5), 115.82 (s, C6), 115.28 (s, C7), 112.28 (s, C8), 72.78 (s, C9), 69.97 (s, C10), 63.90 (s, C11), 55.80 (s, C12), 39.87 (s, C13) ppm

Synthesis of Diol **4**

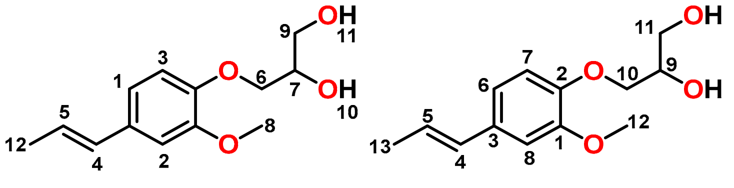

^{1}H NMR Assignments **^{13}C NMR Assignments**

Isoeugenol (4.926 g, 0.030 mol), glycerol carbonate (3.658 g, 0.031 mol), and K_2CO_3 (0.028 g, 0.0002 mol) were added to a 50 mL round bottom flask. The mixture was refluxed under N_2 at 130 °C for 30 min, and the temperature was increased by 10 °C every 30 min until a temperature of 180 °C was reached. At 180 °C, the reaction

proceeded for an additional 2.5 h. The material was extracted using H_2O and EtOAc (50 mL x 3), and the organic layer was dried using Na_2SO_4. The solvent was decanted and removed *via* rotary evaporation to form a yellow oil. The product was purified by column chromatography with 3:1 EtOAc:hexanes mixed with 1 mL of NEt_3 (R_f = 0.17) to yield a beige solid after removing the solvents *via* rotary evaporation. Yield: 38.2 % (2.732 g).

^{1}H NMR ($CDCl_3$, 400 MHz): δ = 6.86-6.81 (m, 3H, H1, H2 and H3), 6.34-6.30 (d, 1H, H4, $^3J_{HHtrans}$ = 17.3 Hz), 6.13-6.08 (dd, 1H, H5, $^3J_{HHcis}$ = 6.6 Hz), 4.01-4.09 (m, 3H, H6 and H7), 3.84 (s, 3H, H8), 3.75-3.78 (m, 2H, H9), 2.98 (br s, 1H, H10), 2.33 (br s, 1H, H11), 1.85-1.90 (dd, 3H, H12, $^3J_{HH}$ = 1.6 Hz) ppm

^{13}C{^{1}H} NMR ($CDCl_3$, 101 MHz): δ = 149.51 (s, C1), 147.03 (s, C2), 132.38 (s, C3), 130.49 (s, C4), 124.42 (s, C5), 118.85 (s, C6), 114.50 (s, C7), 109.00 (s, C8), 71.99 (s, C9), 70.13 (s, C10), 63.83 (s, C11), 55.79 (s, C12), 18.38 (s, C13) ppm

HRMS (DART) *m/z* [$M^+NH_4^+$] calcd for $C_{13}H_{18}O_4$: 256.15433, found: 256.15478

Synthesis of Diol **5**

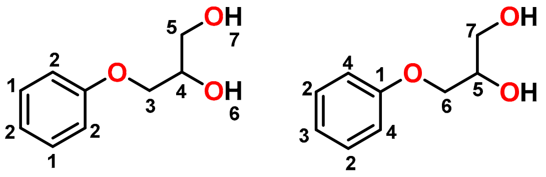

^{1}H NMR Assignments **^{13}C NMR Assignments**

Phenol (2.823 g, 0.030 mol), glycerol carbonate (3.658 g, 0.031 mol), and K_2CO_3 (0.025 g, 0.0002 mol) were added to a 50 mL round bottom flask. The mixture was refluxed under N_2 at 130 °C for 30 min, and the temperature was increased by 10 °C every 30 min until a temperature of 180 °C was reached. At 180 °C, the reaction proceeded for an additional

2.5 h. The material was extracted using brine and EtOAc (50 mL x 3), and the organic layer was dried using Na_2SO_4. The solvent was decanted and removed *via* rotary evaporation to form a pale-yellow oil. The product was left in a desiccator for 2 days, where it solidified to a yellowish-brown waxy solid. Recrystallization was performed using Et_2O to yield an off-white solid. Yield: 80.3 % (4.050 g). Agrees with literature.[1]

^{1}H NMR ($CDCl_3$, 400 MHz): δ = 7.27-7.31 (m, 2H, H1), 6.91-7.00 (m, 3H, H2), 4.04-4.12 (m, 3H, H3 and H4), 3.73-3.87 (m, 2H, H5), 2.72 (s, 1H, H6), 2.17 (s, 1H, H7) ppm

^{13}C{^{1}H} NMR ($CDCl_3$, 101 MHz): δ = 158.39 (s, C1), 129.58 (s, C2), 121.35 (s, C3), 114.54 (s, C4), 70.40 (s, C5), 69.12 (s, C6), 63.69 (s, C7) ppm

Synthesis of Diol **6**

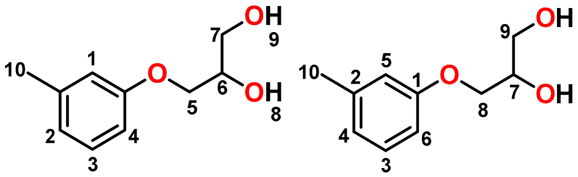

^{1}H NMR Assignments **^{13}C NMR Assignments**

m-cresol (3.244 g, 0.030 mol), glycerol carbonate (3.658 g, 0.031 mol), and K_2CO_3 (0.025 g, 0.0002 mol) were added to a 50 mL round bottom flask. The mixture was refluxed under N_2 at 130 °C for 30 min, and the temperature was increased by 10 °C every 30 min until a temperature of 180 °C was reached. At 180 °C, the reaction proceeded for an additional

2.5 h. The material was extracted using brine and EtOAc (50 mL x 3), and the organic layer was dried using Na_2SO_4. The solvent was decanted and removed using rotary evaporation to form an orange-yellow oil and was dried for an additional 2 days in a desiccator. The product was purified by column chromatography with 3:1 EtOAc:hexanes with 1 mL of NEt_3 (R_f = 0.40). After removing the solvents *via* rotary evaporation and further drying in a desiccator for 2 days, the resulting material was a white powder. Yield: 56.3 % (3.078 g). Agrees with literature.[4]

^{1}H NMR ($CDCl_3$, 400 MHz): δ = 7.13-7.17 (t, 1H, H1, $^3J_{HH}$ = 7.8 Hz), 6.69-6.79 (m, 3H, H2, H3 and H4), 4.07-4.12 (q, 1H, H5, $^3J_{HH}$ = Hz), 4.00 (d, 2H, H6, $^3J_{HH}$ = 5.5 Hz), 3.70-3.90 (m, 2H, H7), 3.02 (br s, 2H, H8 and H9), 2.31 (s, 3H, H10) ppm

^{13}C{^{1}H} NMR ($CDCl_3$, 101 MHz): δ = 158.45 (s, C1), 139.65 (s, C2), 129.30 (s, C3), 122.12 (s, C4), 115.40 (s, C5), 111.45 (s, C6), 70.57 (s, C7), 69.02 (s, C8), 63.74 (s, C9), 21.49 (s, C10) ppm

Characterization of Diol **7**

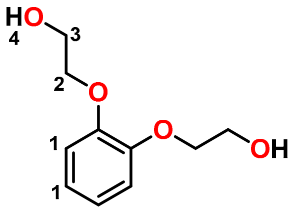

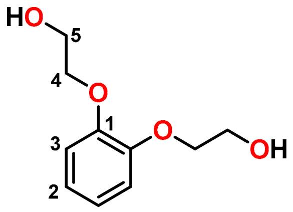

^{1}H NMR Assignments **^{13}C NMR Assignments**

^{1}H NMR ($CDCl_3$, 400 MHz): δ = 6.97 (s, 4H, H1), 4.10-4.13 (t, 4H, H2, $^3J_{HH}$ = 4.5 Hz), 3.91-3.94 (t, 4H, H3, $^3J_{HH}$ = 4.2 Hz), 2.85 (br s, 2H, H4) ppm

^{13}**C{^{1}H} NMR** ($CDCl_3$, 101 MHz): δ = 149.10 (s, C1), 122.52 (s, C2), 115.95 (s, C3), 71.96 (s, C4), 61.23 (s, C5) ppm

Synthesis of Diol **8**

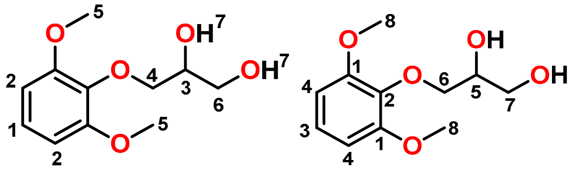

Syringol (5.000 g, 0.0324 mol), glycerol carbonate (3.823 g, 0.0324 mol), and K_2CO_3 (0.025 g, 0.0002 mol) were added to a 50 mL round bottom flask. The mixture was refluxed under N_2 at 130 °C for 30 min, and the temperature was increased by 10 °C every 30 min until a temperature of 180 °C was reached. At 180 °C, the reaction proceeded for an additional 2.5 h. The material was extracted using brine and EtOAc (50 mL x 3), and the organic layer was dried using Na_2SO_4. The solvent was decanted and removed *via* rotary evaporation to form an orange-yellow oil and was dried for an

additional 2 days in a desiccator. The product was purified by column chromatography with 3:1 EtOAc:hexanes with 1 mL of NEt_3 (R_f = 0.29) to yield a pale-yellow oil after removing the solvents *via* rotary evaporation. Yield: 50 % (3.702 g). Agrees with literature.[5]

^{1}H NMR ($CDCl_3$, 400 MHz): δ = 6.97-7.02 (t, 1H, H1, $^3J_{HH}$ = 8.4 Hz), 6.56-6.58 (d, 2H, H2, $^3J_{HH}$ = 8.4 Hz), 4.18-4.23 (q, 1H, H3, $^3J_{HH}$ = 6.9 Hz), 3.93-3.94 (d, 2H, H4, $^3J_{HH}$ = 6.9 Hz), 3.84 (s, 6H, H5), 3.65-3.75 (m, 2H, H6), 3.22 (br s, 2H, H7) ppm

^{13}C{^{1}H} NMR ($CDCl_3$, 101 MHz): δ = 153.16 (s, C1), 136.81 (s, C2), 124.09 (s, C3), 105.15 (s, C4), 75.81 (s, C5), 70.48 (s, C6), 63.49 (s, C7), 56.06 (s, C8) ppm

Synthesis of Diol **9**

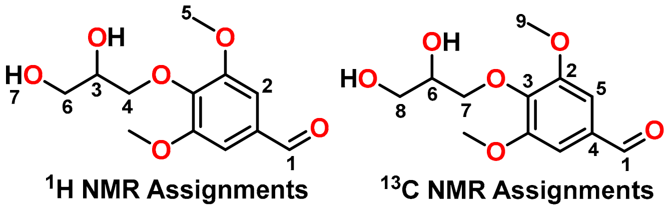

Syringaldehyde (2.000 g, 0.0110 mol), glycerol carbonate (1.303 g, 0.0110 mol), and K_2CO_3 (0.016 g, 0.0001 mol) were added to a 50 mL round bottom flask. The mixture was refluxed under N_2 at 130 °C for 30 min, and the temperature was increased by 10 °C every 30 min until a temperature of 180 °C was reached. At 180 °C, the reaction proceeded for an additional 2 h. The material was extracted using H_2O and EtOAc (50 mL x 4), and the organic layer was dried using Na_2SO_4. The product was purified by column chromatography with 3:1 EtOAc:hexanes (R_f = 0.14). The solvent was removed

via rotary evaporation to form an off-white powder that was further dried in a desiccator for 3 days. Yield: 10 % (0.270 g).

^{1}H NMR ($CDCl_3$, 400 MHz): δ = 9.86 (s, 1H, H1), 7.13-7.15 (d, 2H, H2, $^4J_{HH}$ = 11.3 Hz), 4.28-4.31 (m, 1H, H3), 3.99-4.04 (m, 1H, H4), 3.93-3.94 (d, 6H, H5), 3.83-3.89 (m, 1H, H4), 3.67-3.79 (m, 2H, H6), 2.73 (br s, 2H, H7) ppm

^{13}C{^{1}H} NMR ($CDCl_3$, 101 MHz): δ = 190.98 (s, C1), 153.43 (s, C2), 142.25 (s, C3), 132.11 (s, C4), 106.58 (s, C5), 75.95 (s, C6), 70.53 (s, C7), 63.33 (s, C8), 56.30 (s, C9) ppm

HRMS (DART) *m/z* [M^+H^+] calcd for $C_{12}H_{16}O_6$: 257.10196, found: 257.10228

Synthesis of Diol **10**

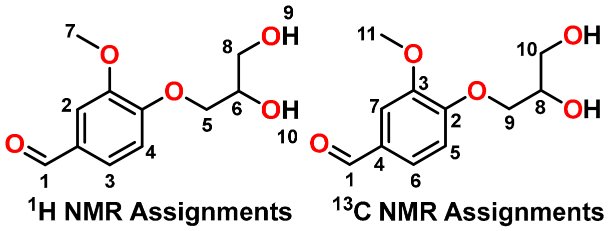

Vanillin (2.085 g, 13.7 mmol), glycerol carbonate (1.576 g, 13.3 mmol) and K_2CO_3 (0.017 g, 0.123 mmol) were added to a 50 mL round bottom flask. A reflux condenser was attached to the flask and its contents were refluxed under N_2 at 130 °C for 30 min. The temperature was increased by 10 °C every 30 min until 180 °C was reached. The reaction mixture was refluxed for an additional 2 h at 180 °C. The resulting amber coloured mixture was dissolved in $CHCl_3$ and extracted with dH_2O. The combined organic layers were dried with Na_2SO_4, and the solvent was removed *via* rotary evaporation. The product was

purified through column chromatography with 3:1 EtOAc/Hexane (R_f = 0.21). The solvent was removed *via* rotary evaporation to yield a brown solid that was placed in a desiccator for a week. Yield: 11.1 % (0.33 g). Agrees with literature.[3]

^{1}H NMR ($CDCl_3$, 400 MHz): δ = 9.84 (s, 1H, H1), 7.40-7.44 (m, 2H, H2 and H3), 6.97-6.99 (d, $^3J_{H-H}$ = 8.1 Hz, 1H, H4), 4.08-4.21 (m, 3H, H5 and H6), 3.90 (s, 3H, H7), 3.80-3.87 (m, 2H, H8), 2.59 (br s, 2H, H9 & H10) ppm

^{13}C{^{1}H} NMR ($CDCl_3$, 101 MHz): δ = 190.91 (C1), 153.40 (C2), 149.83 (C3), 130.66 (C4), 126.73 (C5), 112.13 (C6), 109.33 (C7), 71.41 (C8), 69.77 (C9), 63.66 (C10), 55.97 (C11) ppm

NMR and Mass Spectra of Diols 4 and 9

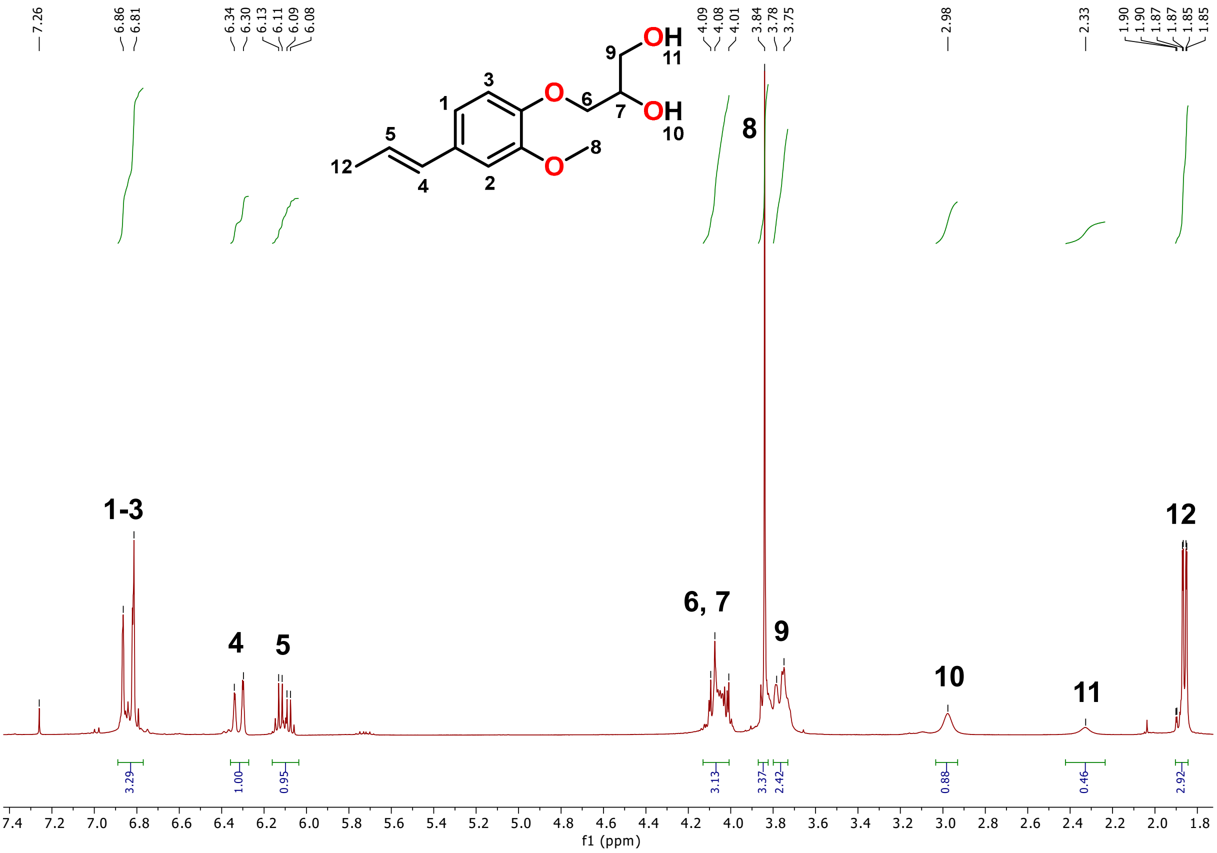

Figure S1: ^{1}H NMR of **4** in $CDCl_3$

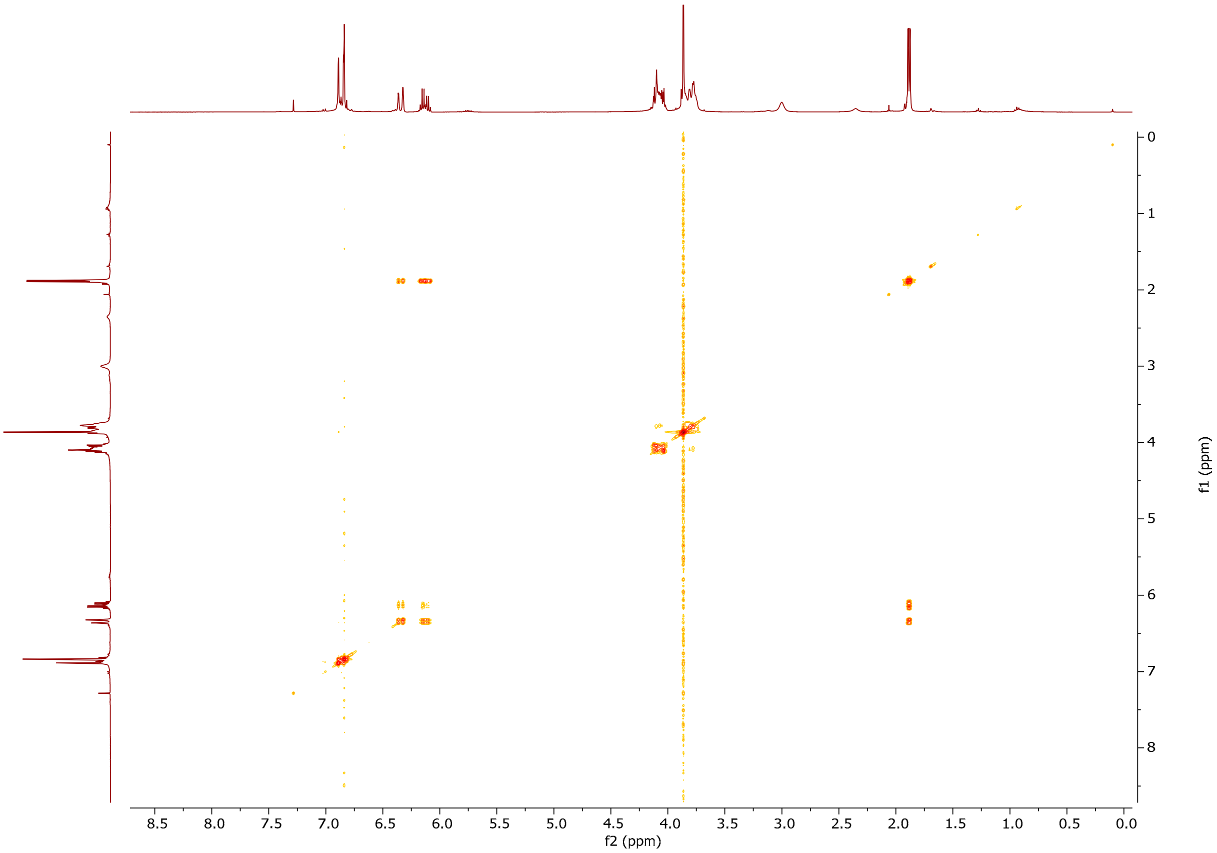

Figure S2: 2D COSY NMR of **4** in $CDCl_3$

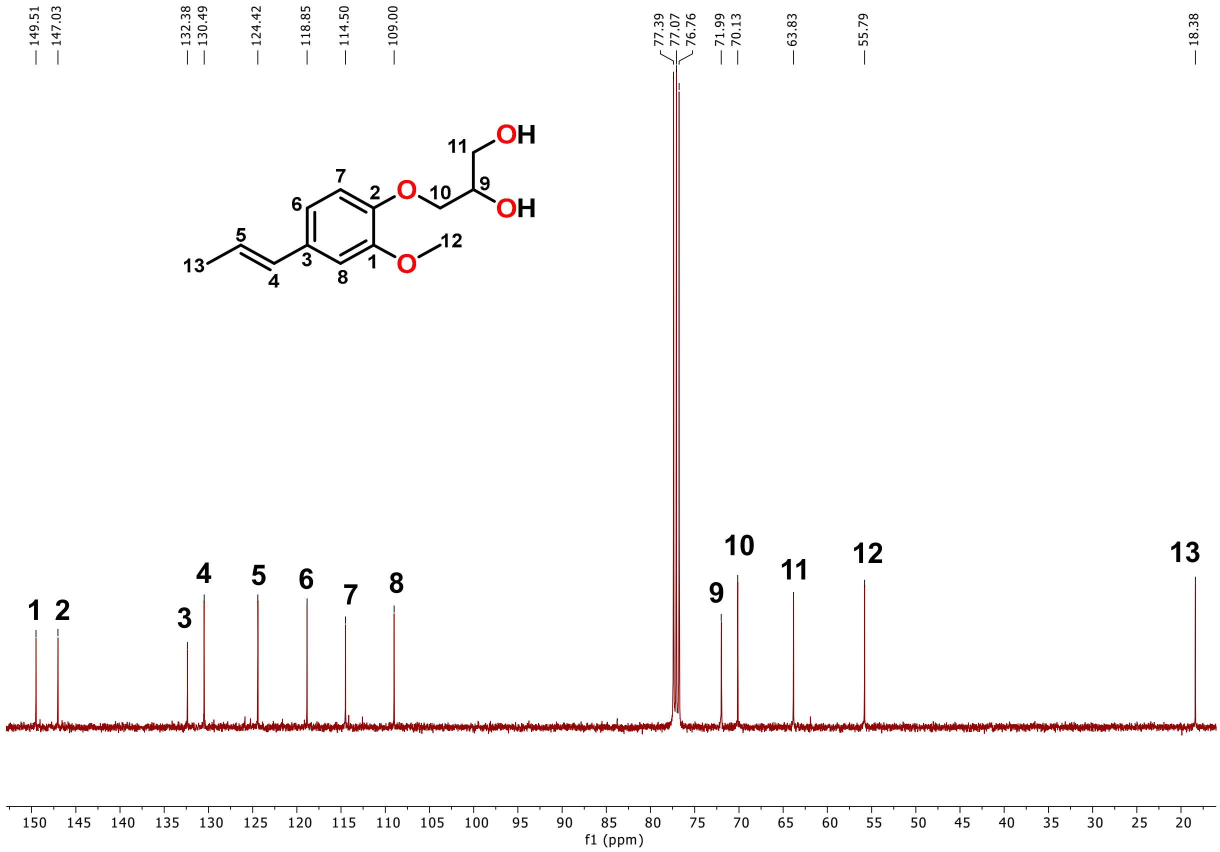

Figure S3: ^{13}C NMR of **4** in $CDCl_3$

Spectrum

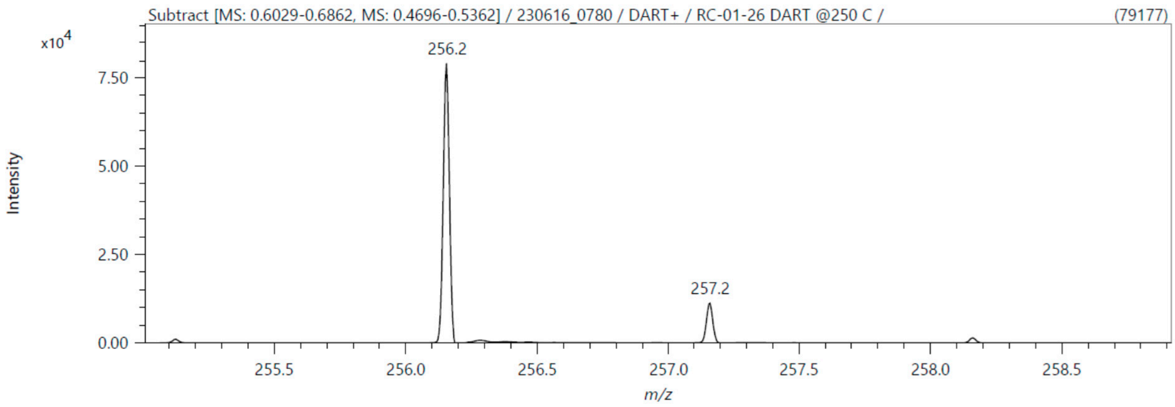

Elemental Composition

Parameters		Elements Set 1:				
Tolerance:	±10.00 mDa	Symbol	C	H	O	N
Electron:	Even	Min	0	0	0	0
Charge:	+1	Max	100	200	20	10
DBE:	-1.5 - 100.0					

Results

Mass	Intensity	Formula	Calculated Mass	Mass Difference [mDa]	Mass Difference [ppm]	DBE
256.15478	79177.10	C13 H22 N O4	256.15433	0.44	1.72	3.5
		C14 H18 N5	256.15567	-0.90	-3.50	8.5
		C9 H18 N7 O2	256.15165	3.13	12.20	4.5
		C8 H22 N3 O6	256.15031	4.46	17.42	-0.5
		C7 H22 N5 O5	256.16155	-6.77	-26.43	-0.5
		C4 H18 N9 O4	256.14763	7.15	27.91	0.5

Figure S4: HRMS (DART) Analysis of **4**

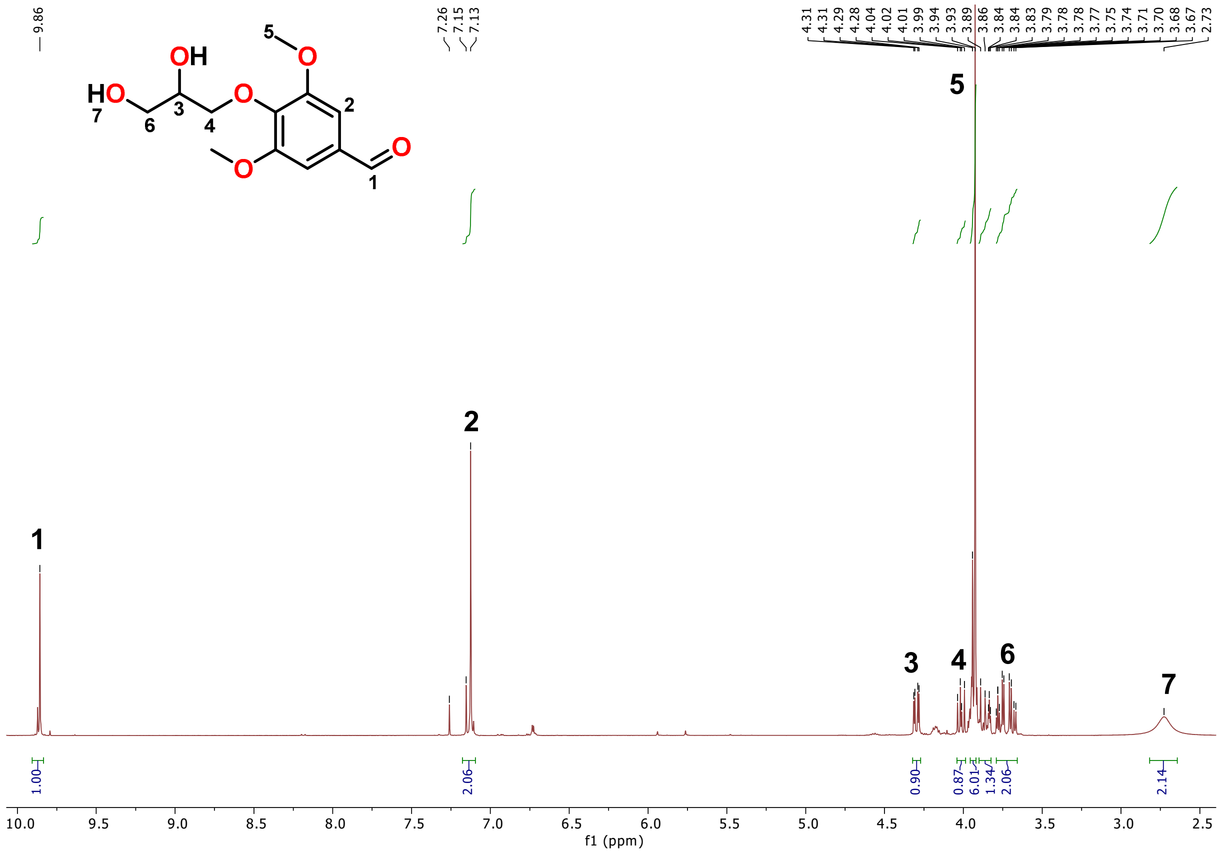

Figure S5: ^{1}H NMR of **9** in $CDCl_3$

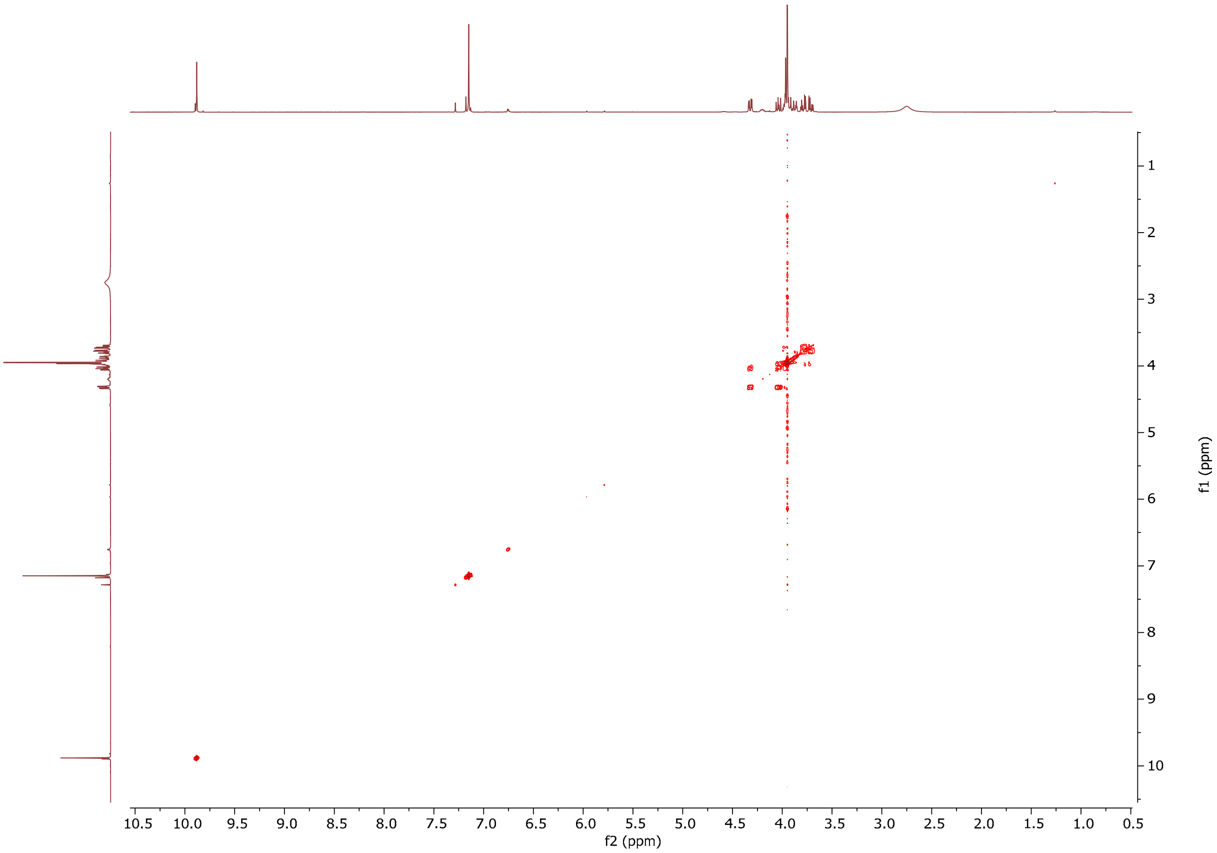

Figure S6: 2D COSY NMR of **9** in $CDCl_3$

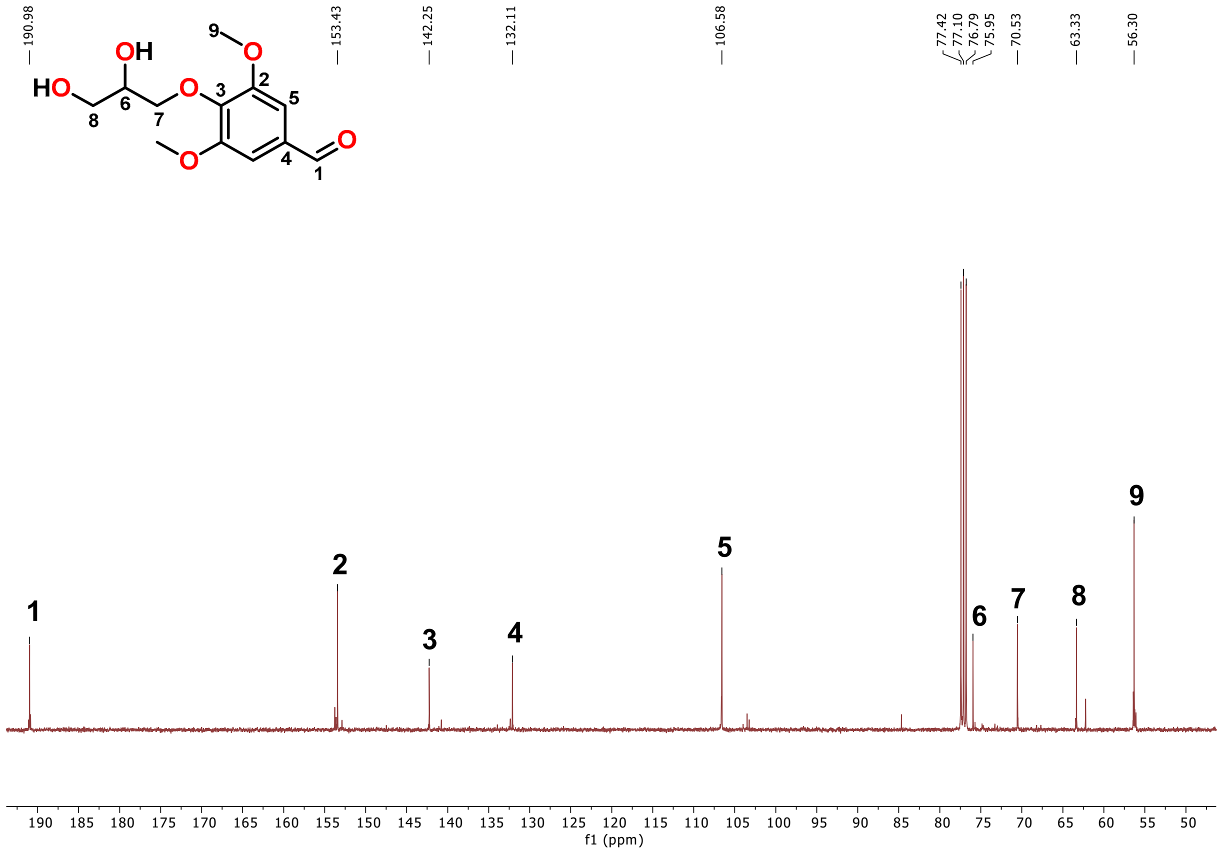

Figure S7: ^{13}C NMR of **9** in $CDCl_3$

Spectrum

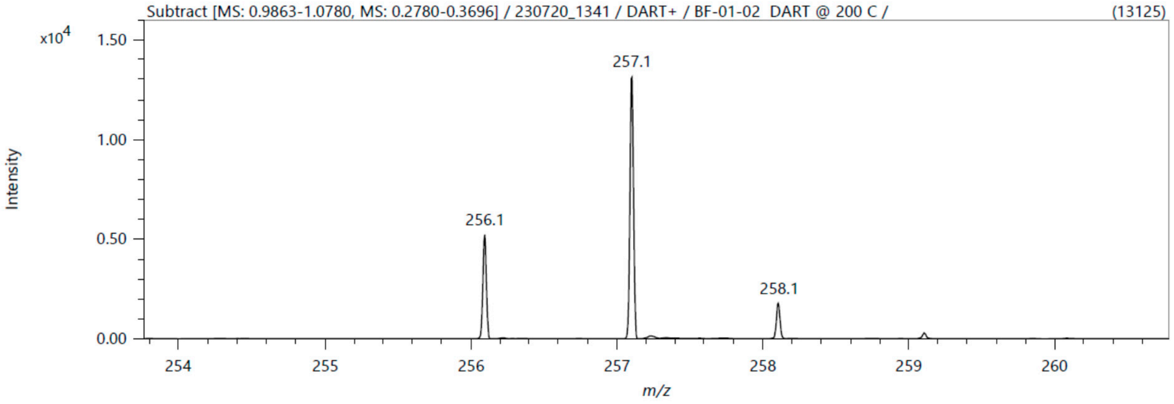

Elemental Composition

Parameters		Elements Set 1:				
		Symbol	C	H	O	N
Tolerance:	±10.00 mDa	Min	0	0	0	0
Electron:	Even	Max	100	200	20	10
Charge:	+1					
DBE:	-1.5 - 100.0					

Results

Mass	Intensity	Formula	Calculated Mass	Mass Difference [mDa]	Mass Difference [ppm]	DBE
257.10228	13125.50	C12 H17 O6	257.10196	0.31	1.22	4.5
		C13 H13 N4 O2	257.10330	-1.02	-3.98	9.5
		C9 H9 N10	257.10062	1.66	6.46	10.5
		C8 H13 N6 O4	257.09928	3.00	11.67	5.5
		C2 H13 N10 O5	257.10649	-4.21	-16.38	1.5
		C7 H17 N2 O8	257.09794	4.34	16.87	0.5

Figure S8: HRMS (DART) Analysis of **9**

Polyurethane NMR Data

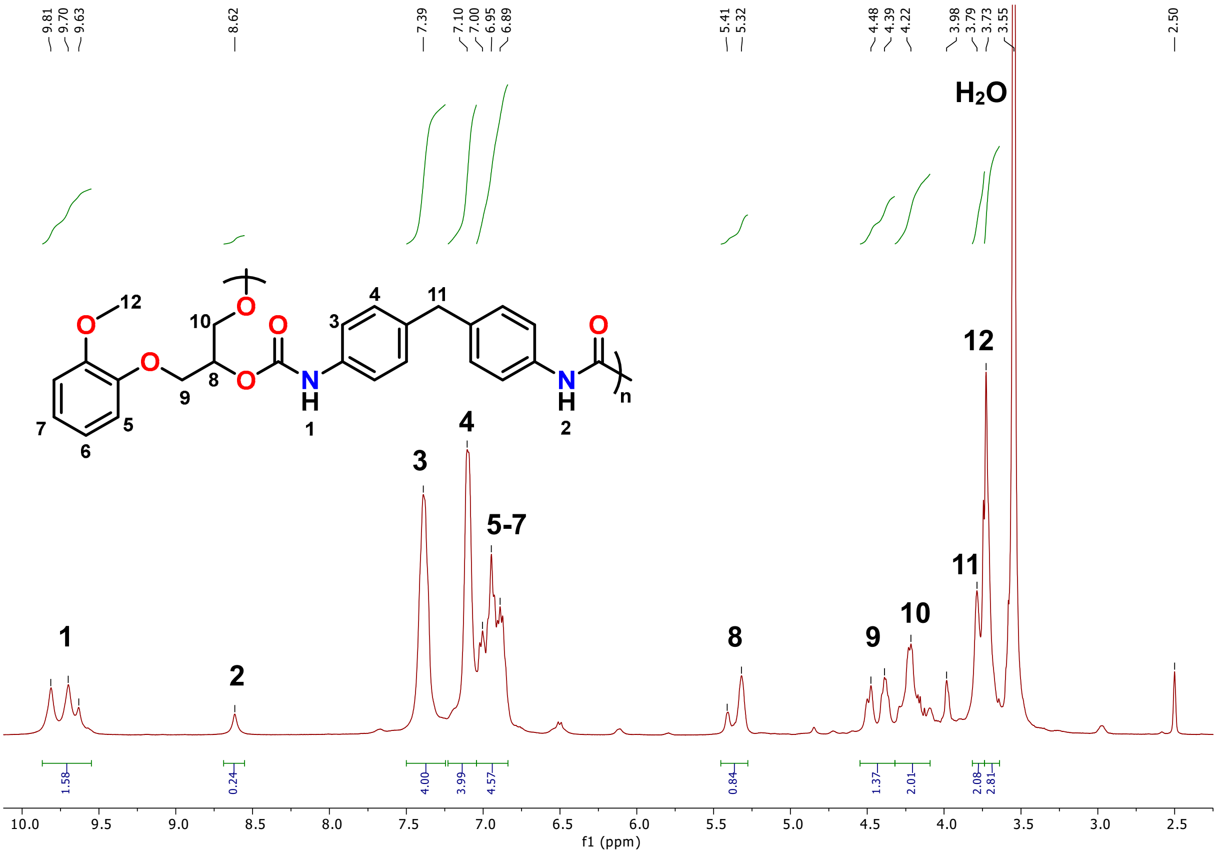

Figure S9: ^{1}H NMR of **PU-1a** in DMSO-d_6

^{1}H NMR (DMSO-d_6): δ = 9.63-9.81 (br t, 2H, H1 and H2), 7.39 (br s, 4H, H3), 7.10 (br s, 4H, H4), 6.89-7.00 (br m, 5H, H5, H6 and H7). 5.32-5.41 (br d, 1H, H8), 4.39-4.48 (br d, 2H, H9), 4.22 (br m, 2H, H10), 3.79 (br s, 2H, H11), 3.73 (br s, 3H, H12) ppm

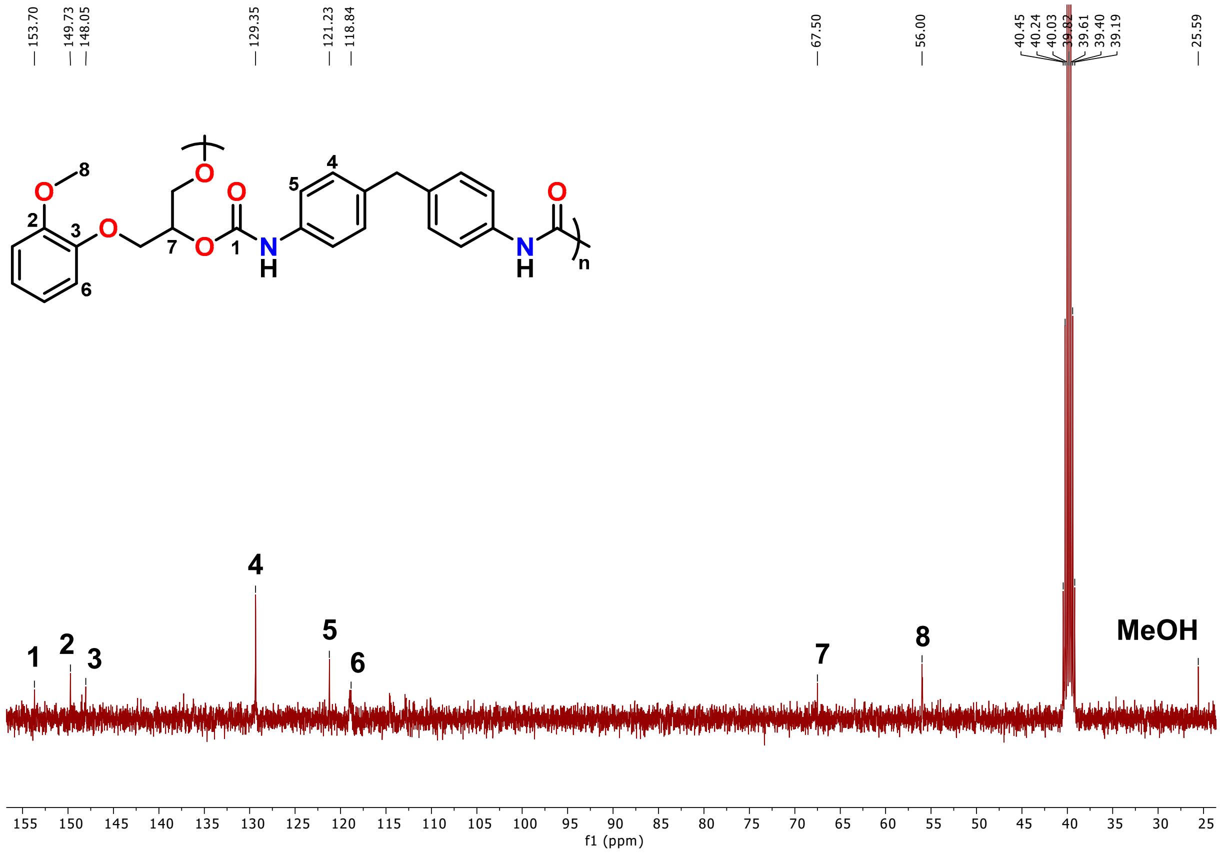

Figure S10: ^{13}C NMR of **PU-1a** in DMSO-d$_6$

^{13}C NMR (DMSO-d$_6$): δ = 153.70 (s, C1), 149.73 (s, C2), 148.05 (s, C3), 129.35 (s, C4), 121.23 (s, C5), 118.84 (s, C6), 67.50 (s, C7), 56.00 (s, C8) ppm (other carbon signals not observed)

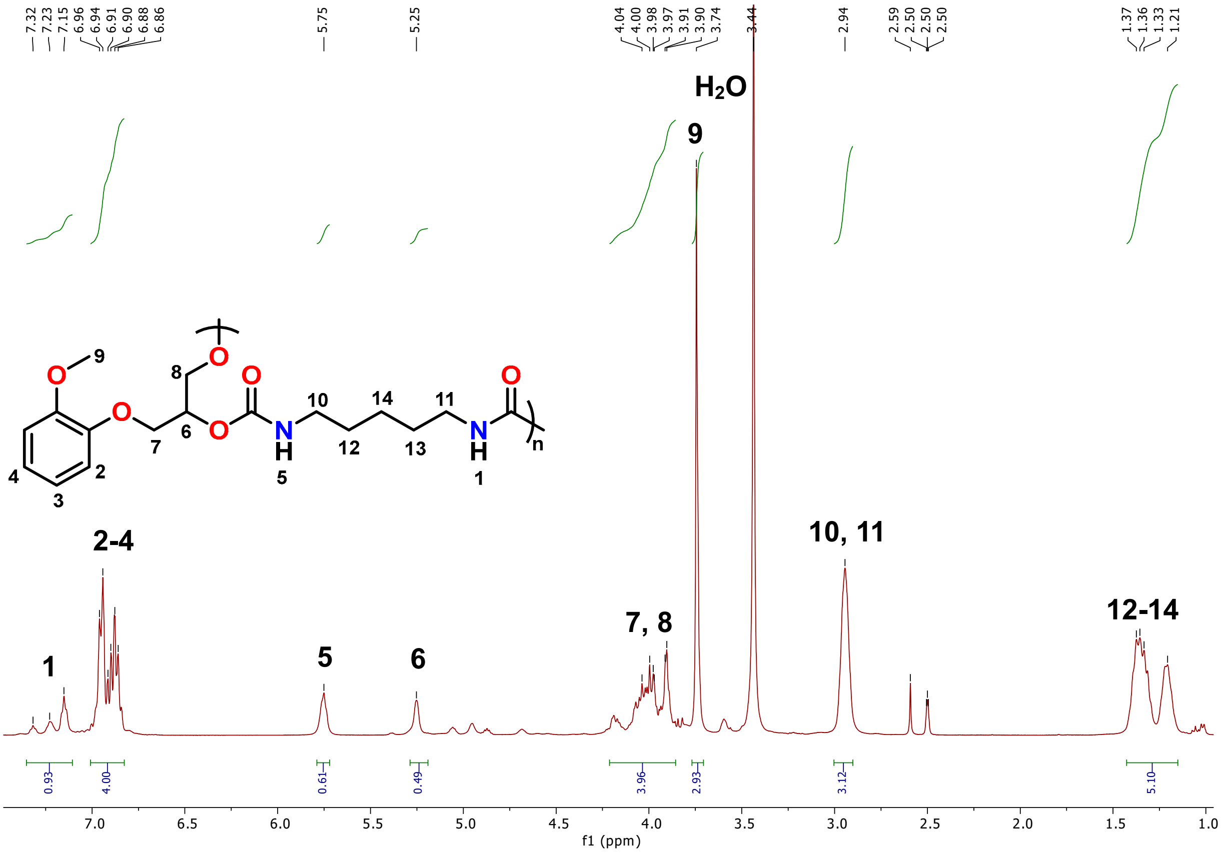

Figure S11: ^{1}H NMR of **PU-1b** in DMSO-d$_6$

^{1}H NMR (DMSO-d$_6$): δ = 7.15-7.32 (br t, 1H, H1), 6.86-6.96 (m, 4H, H2, H3 and H4), 5.75 (br s, 1H, H5), 5.25 (br s, 1H, H6), 3.74-4.04 (m, 4H, H7 and H8), 3.74 (br s, 3H, H9), 2.94 (br s, 3H, H10 and H11), 1.21-1.37 (br m, 5H, H12, H13 and H14) ppm

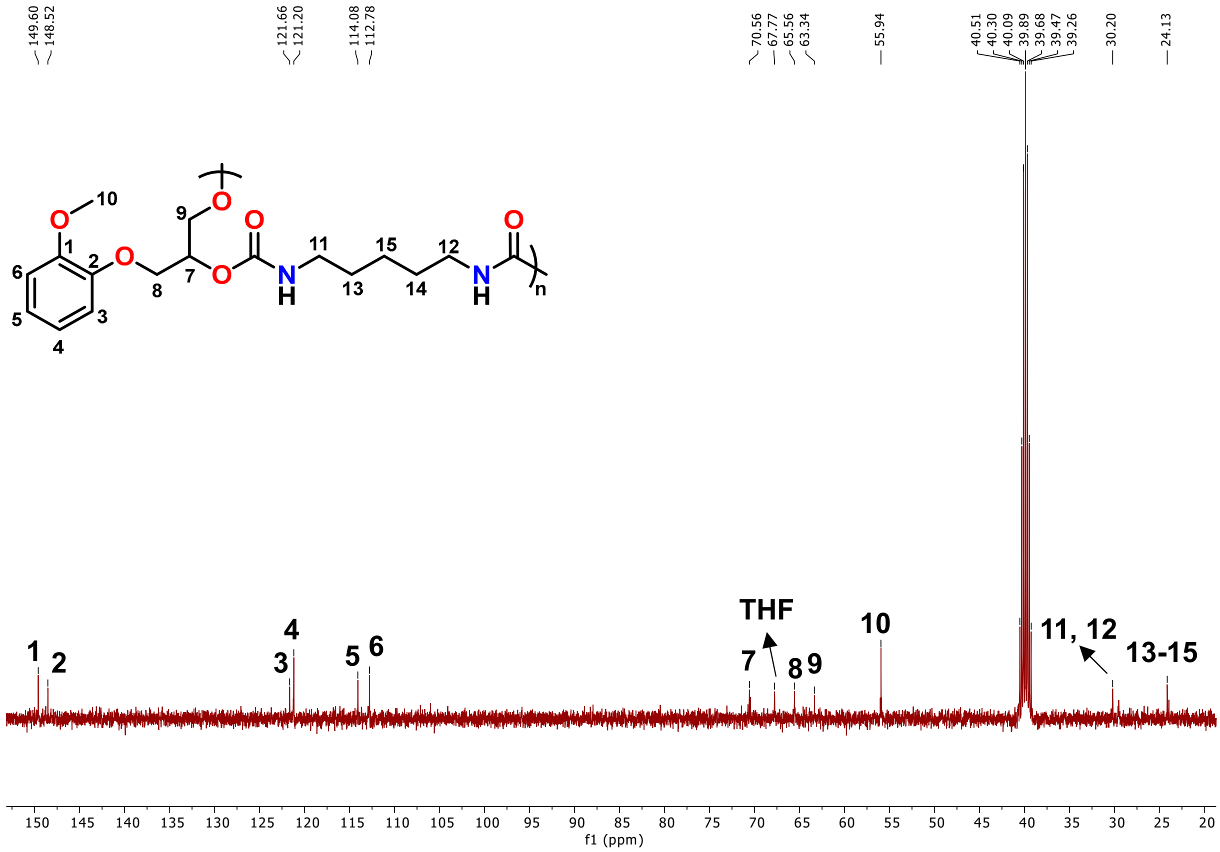

Figure S12: ^{13}C NMR of **PU-1b** in DMSO-d6

^{13}C NMR (DMSO-d6): δ =149.60 (s, C1), 148.52 (s, C2), 121.66 (s, C3), 121.20 (s, C4), 114.08 (s, C5), 112.78 (s, C6), 70.56 (s, C7), 65.56 (s, C8), 63.34 (s, C9), 55.94 (s, C10), 30.20 (s, C11 and C12), 24.13 (s, C13, C14 and C15) ppm (other carbon signals not observed)

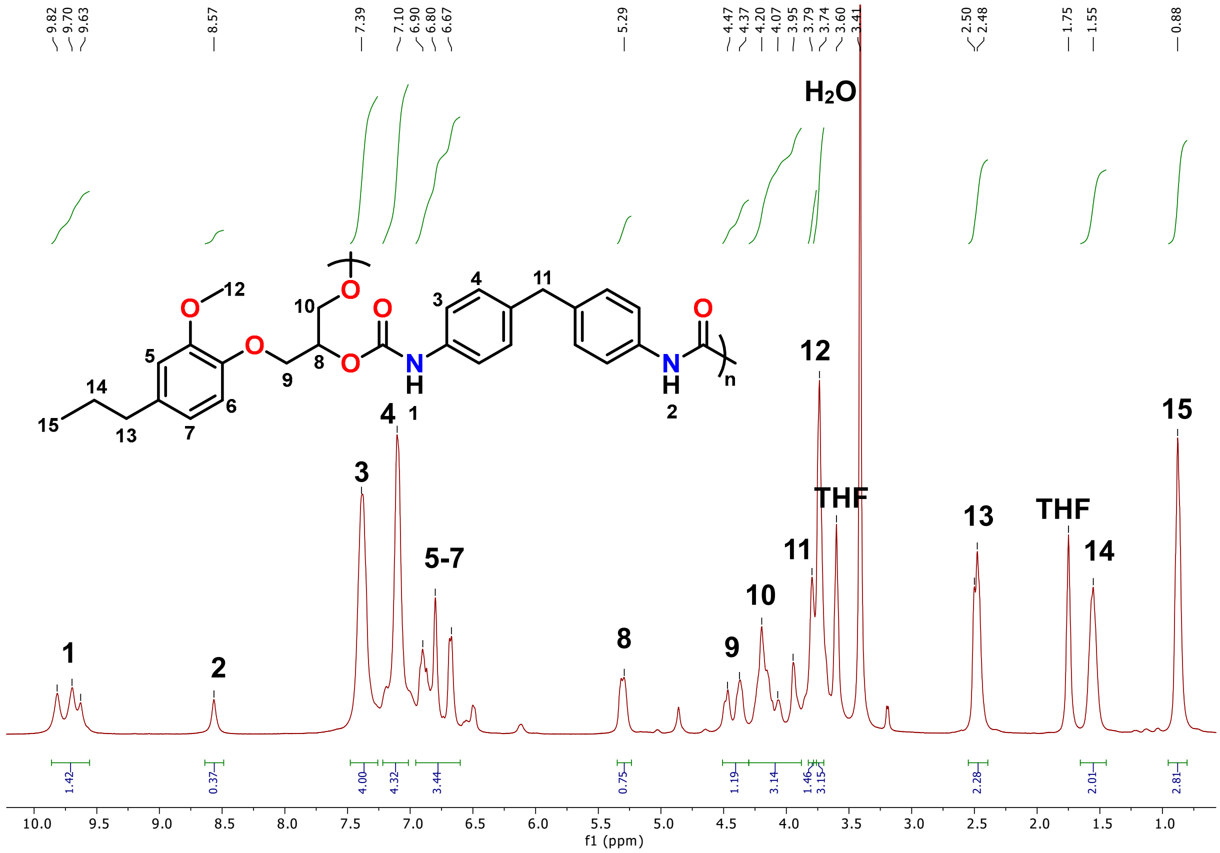

Figure S13: ^{1}H NMR of **PU-2a** in DMSO-d_6

^{1}H NMR (DMSO-d_6): δ = 9.63-9.82 (br t, 1H, H1), 8.57 (br s, 1H, H2), 7.39 (br s, 4H, H3), 7.10 (br s, 4H, H4), 6.67-6.90 (m, 3H, H5, H6 and H7), 5.29 (br s, 1H, H8), 4.37-4.47 (br d, 1H, H9), 3.95-4.20 (br m, 3H, H10), 3.79 (br s, 2H, H11), 3.74 (br s, 3H, H12), 2.48 (br s, 2H, H13), 1.55 (br s, 2H, H14), 0.88 (br s, 3H, H15) ppm

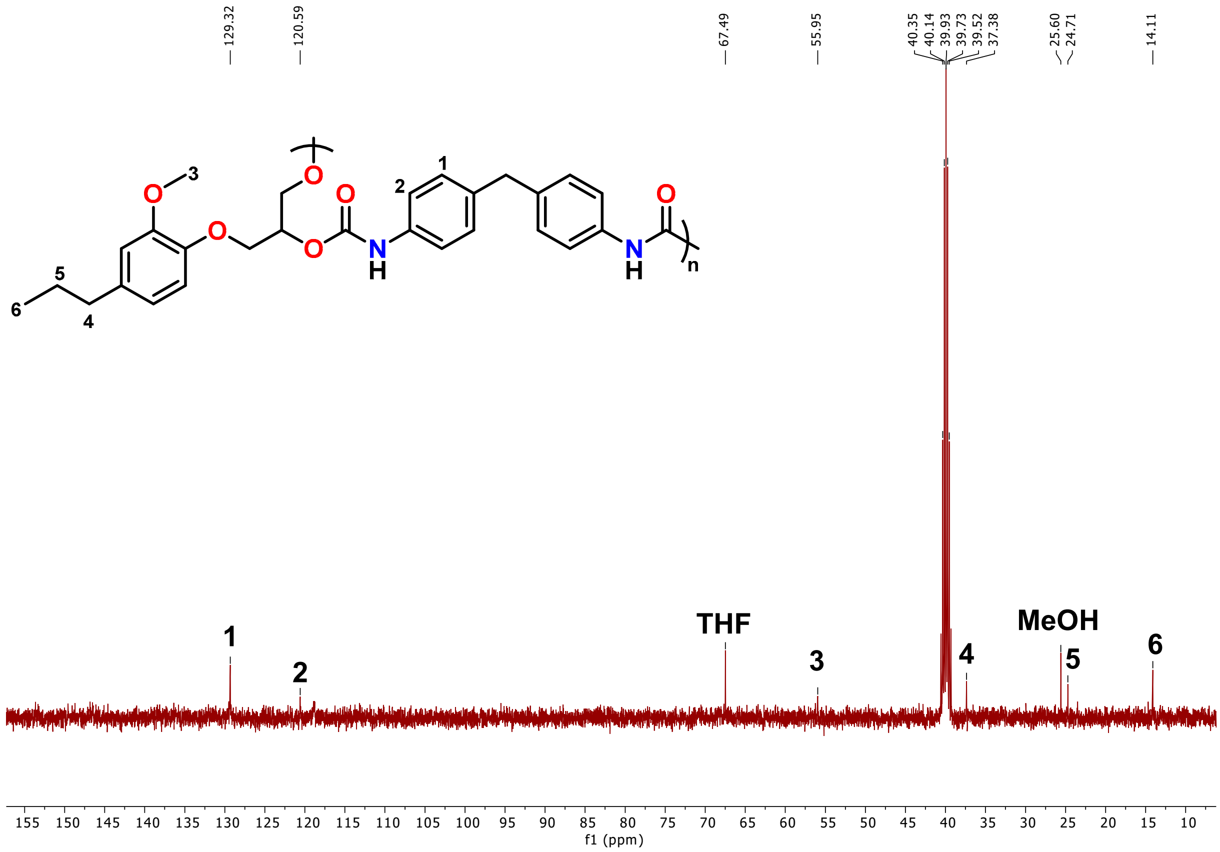

Figure S14: ^{13}C NMR of **PU-2a** in DMSO-d6

^{13}C NMR (DMSO-d6): δ = 129.32 (s, C1), 120.59 (s, C2), 55.95 (s, C3), 37.38 (s, C4), 24.71 (s, C5), 14.11 (s, C6) ppm (other carbon signals not observed)

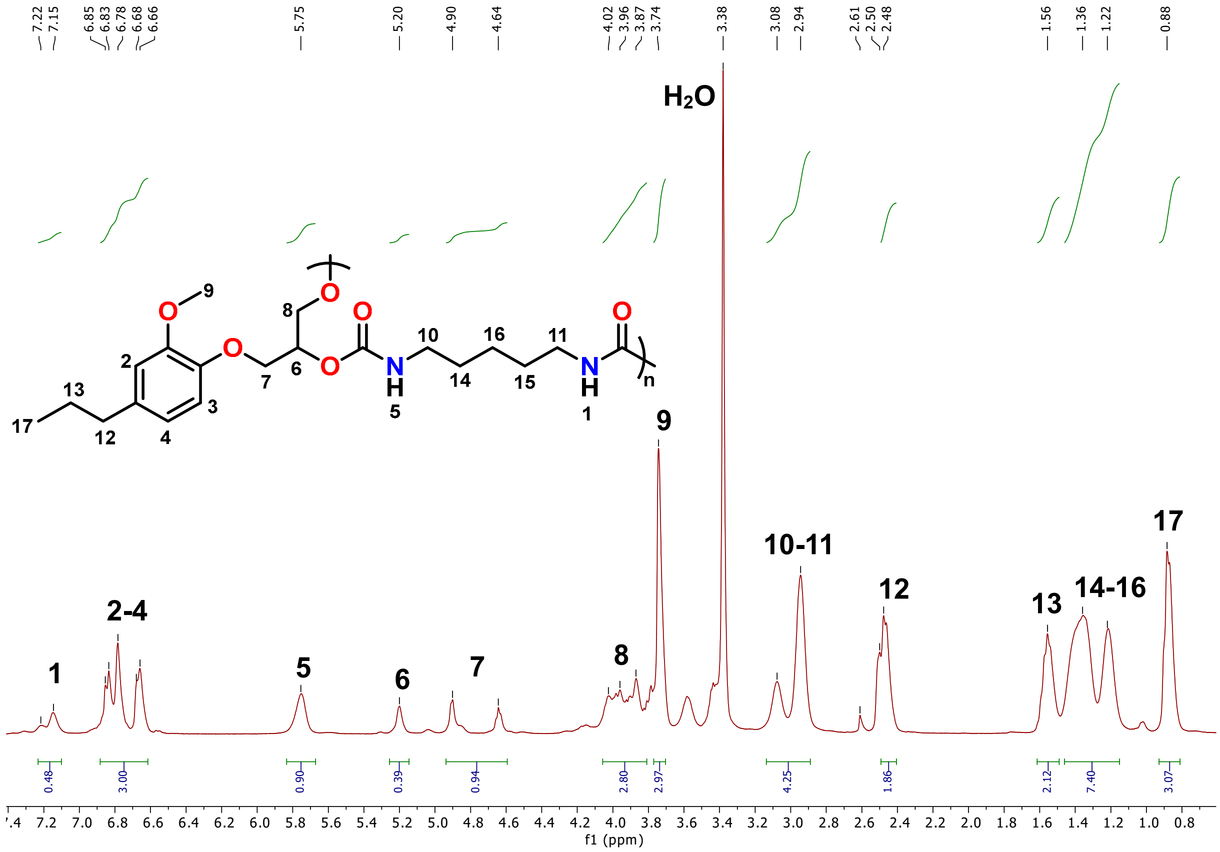

Figure S15: ^{1}H NMR of **PU-2b** in DMSO-d6

^{1}H NMR (DMSO-d6): δ = 7.15-7.22 (br d, 1H, H1), 6.66-6.85 (m, 3H, H2, H3 and H4), 5.75 (br s, 1H, H5), 5.20 (br s, 1H, H6), 4.64-4.90 (br d, 1H, H7), 3.87-4.02 (br m, 3H, H8), 3.74 (br s, 3H, H9), 2.94-3.08 (br d, 4H, H10 and H11), 2.48 (br s, 2H, H12), 1.56 (br s, 2H, H13), 1.22-1.36 (br d, 7H, H14, H15 and H16), 0.88 (br s, 3H, H17) ppm

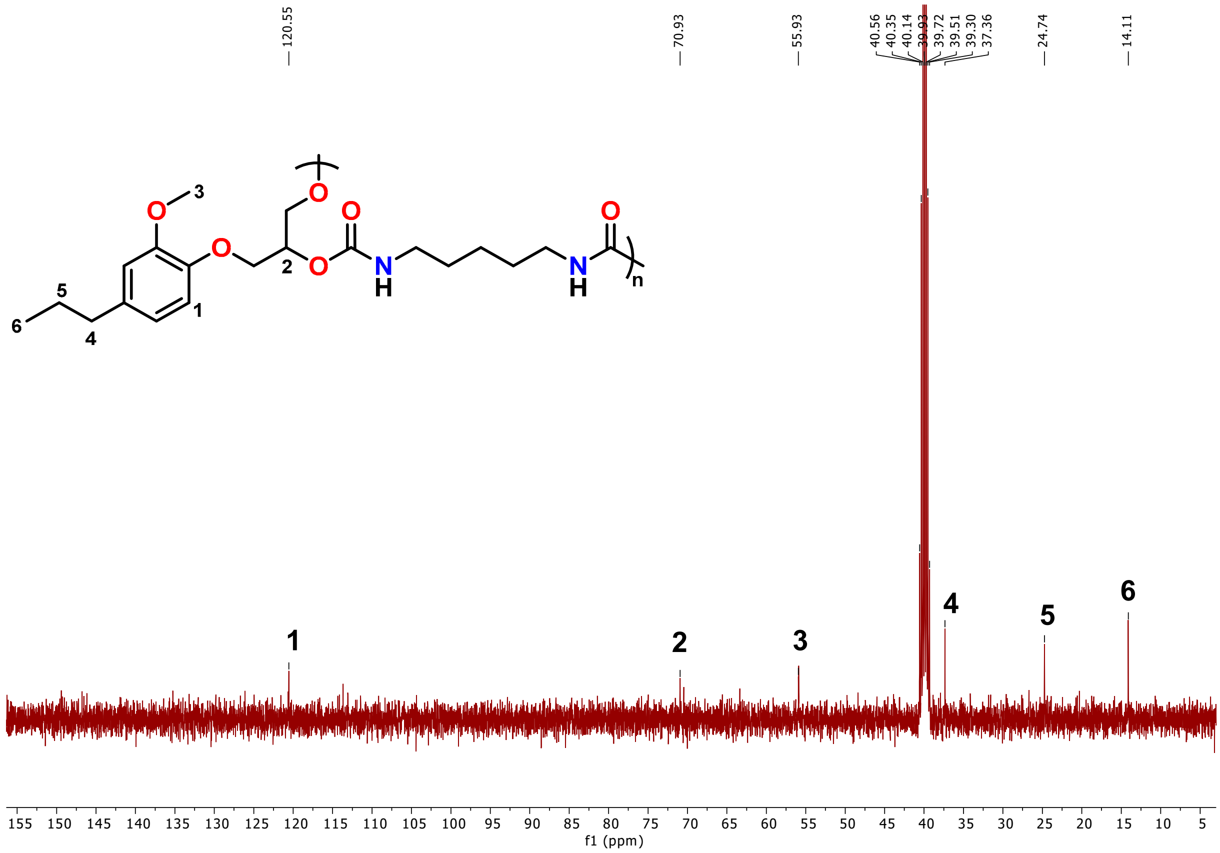

Figure S16: ^{13}C NMR of **PU-2b** in DMSO-d$_6$

^{13}C NMR (DMSO-d$_6$): δ = 120.55 (s, C1), 70.93 (s, C2), 55.93 (s, C3), 37.36 (s, C4), 24.74 (s, C5), 14.11 (s, C6) ppm (other carbon signals not observed)

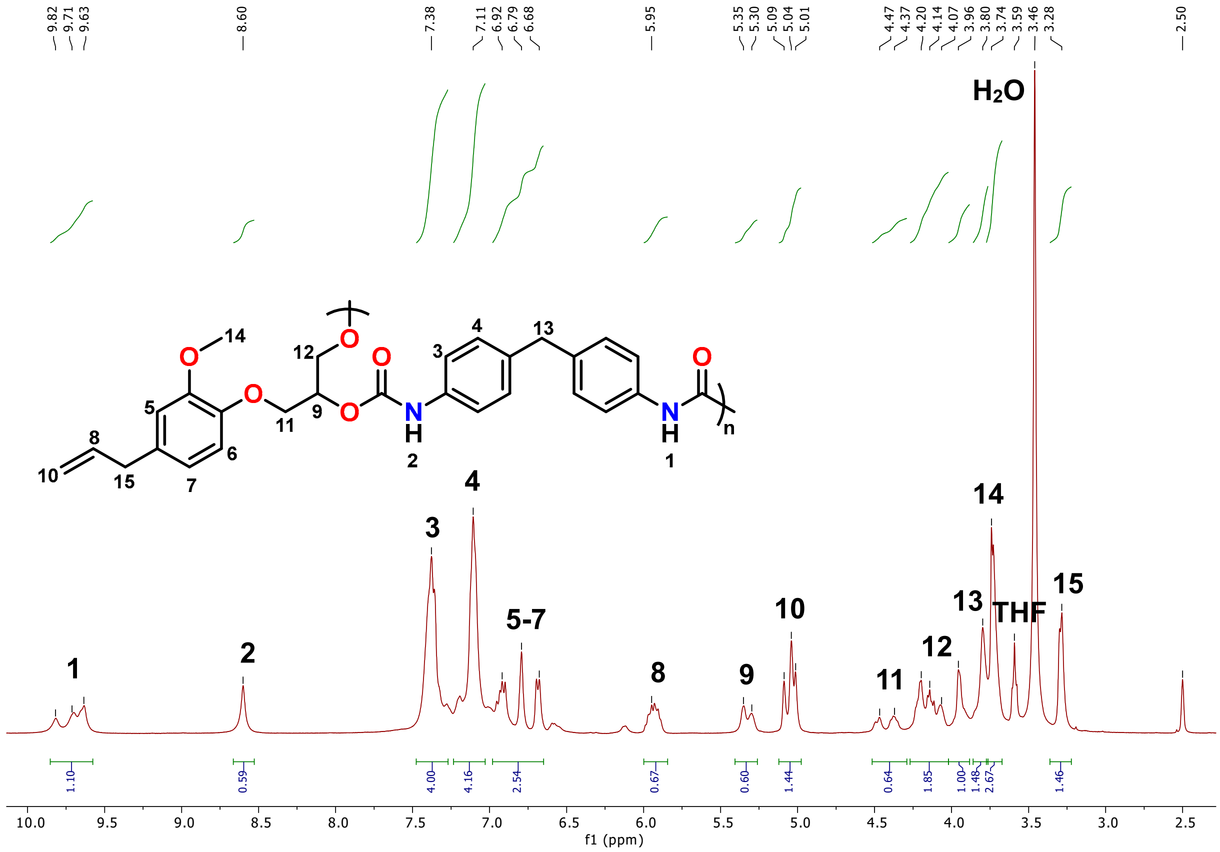

Figure S17: ^{1}H NMR of **PU-3a** in DMSO-d6

^{1}H NMR (DMSO-d6): δ = 9.63-9.82 (br t, 1H, H1), 8.60 (br s, 1H, H2), 7.38 (br s, 4H, H3), 7.11 (br s, 4H, H4), 6.68-6.92 (m, 3H, H5, H6 and H7), 5.95) m, 1H, H8), 5.30-5.35 (br d, 1H, H9), 5.01-5.09 (m, 2H, H10), 4.37-4.47 (br d, 1H, H11), 4.07-4.20 (m, 2H, H12), 3.96 (br s, 1H, H?), 3.80 (br s, 2H, H13), 3.74 (br s, 3H, H14), 3.28 (br s, 2H, H15) ppm

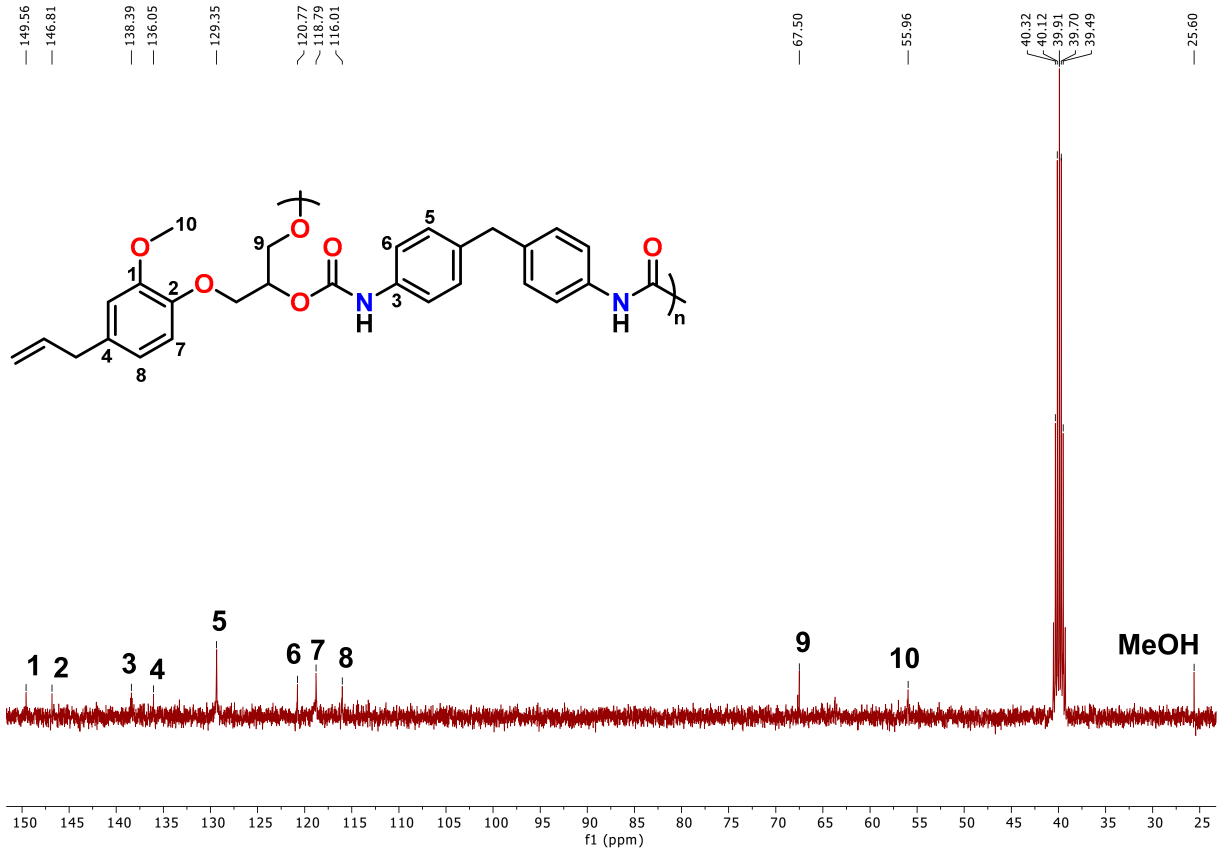

Figure S18: ^{13}C NMR of **PU-3a** in DMSO-d_6

^{13}C NMR (DMSO-d_6): δ = 149.56 (s, C1), 146.81 (s, C2), 138.39 (s, C3), 136.05 (s, C4), 129.35 (s, C5), 120.77 (s, C6), 118.79 (s, C7), 116.01 (s, C8), 67.50 (s, C9), 55.96 (s, C10) ppm (other carbon signals not observed)

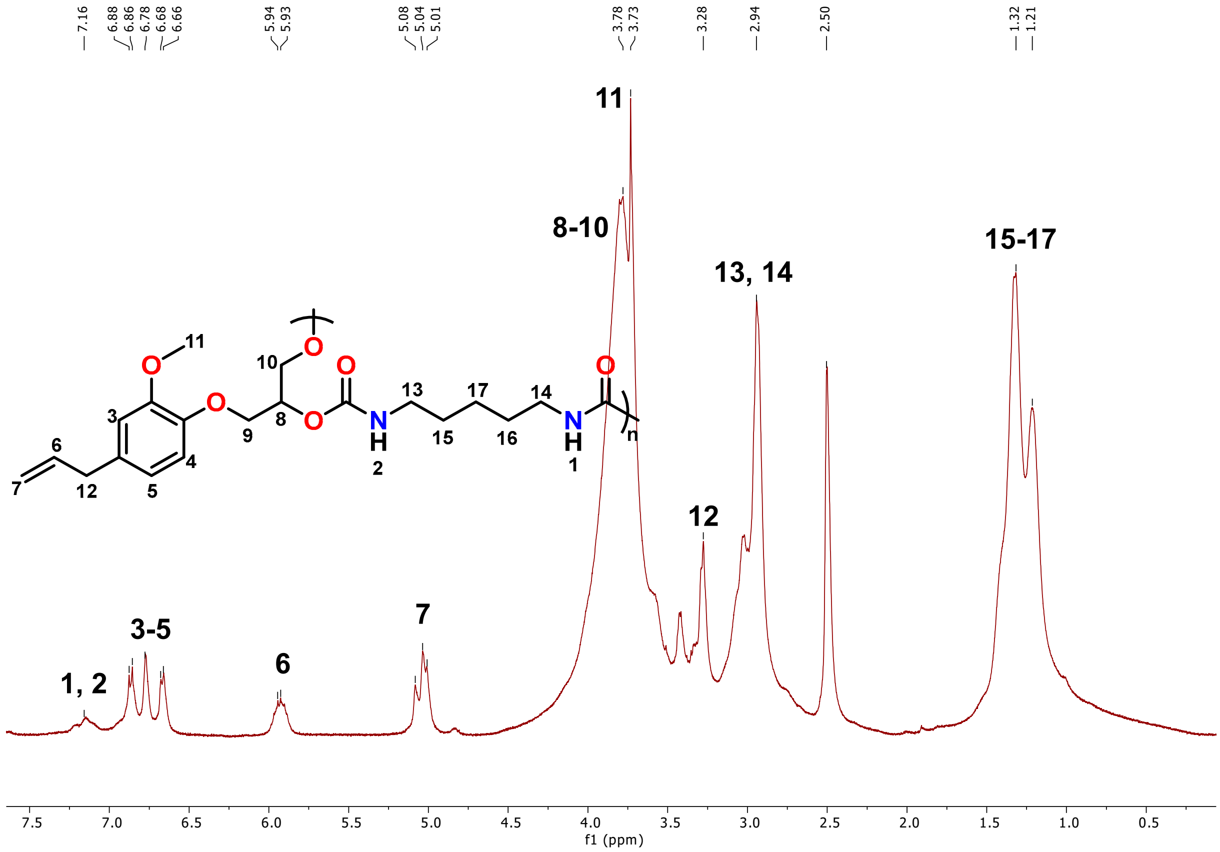

Figure S19: ^{1}H NMR of **PU-3b** in DMSO-d_6

^{1}H NMR (DMSO-d_6): δ = 7.16 (H1 and H2), 6.66-6.88 (H3, H4 and H5), 5.93-5.94 (H6), 5.01-5.08 (H7), 3.78 (H8, H9 and H10), 3.73 (H11), 3.28 (H12), 2.94 (H13 and H14), 1.21-1.32 (H15, H16 and H17) ppm

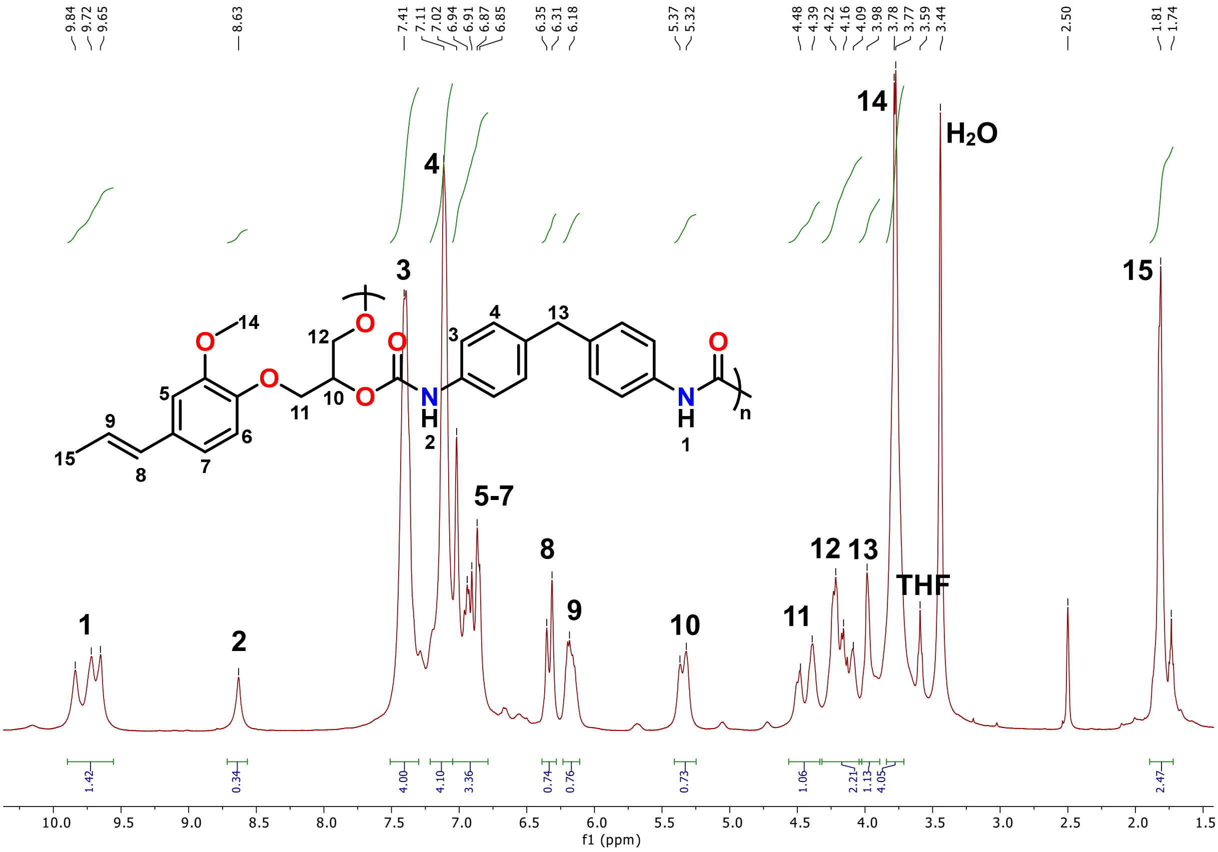

Figure S20: ^{1}H NMR of **PU-4a** in DMSO-d6

^{1}H NMR (DMSO-d6): δ = 9.65-9.84 (br t, 1H, H1), 8.63 (br s, 1H, H2), 7.41 (br s, 4H, H3), 7.11 (br s, 4H, H4), 6.85-7.02 (m, 3H, H5, H6 and H7), 6.31-6.35 (d, 1H, H8), 6.18 (br m, 1H, H9), 5.32-5.37 (br d, 1H, H10), 4.39-4.48 (br d, 1H, H11), 4.09-4.22 (br m, 2H, H12), 3.98 (br s, 1H, H13), 3.77-3.78 (br d, 4H, H14), 1.74-1.81 (br d, 3H, H15) ppm

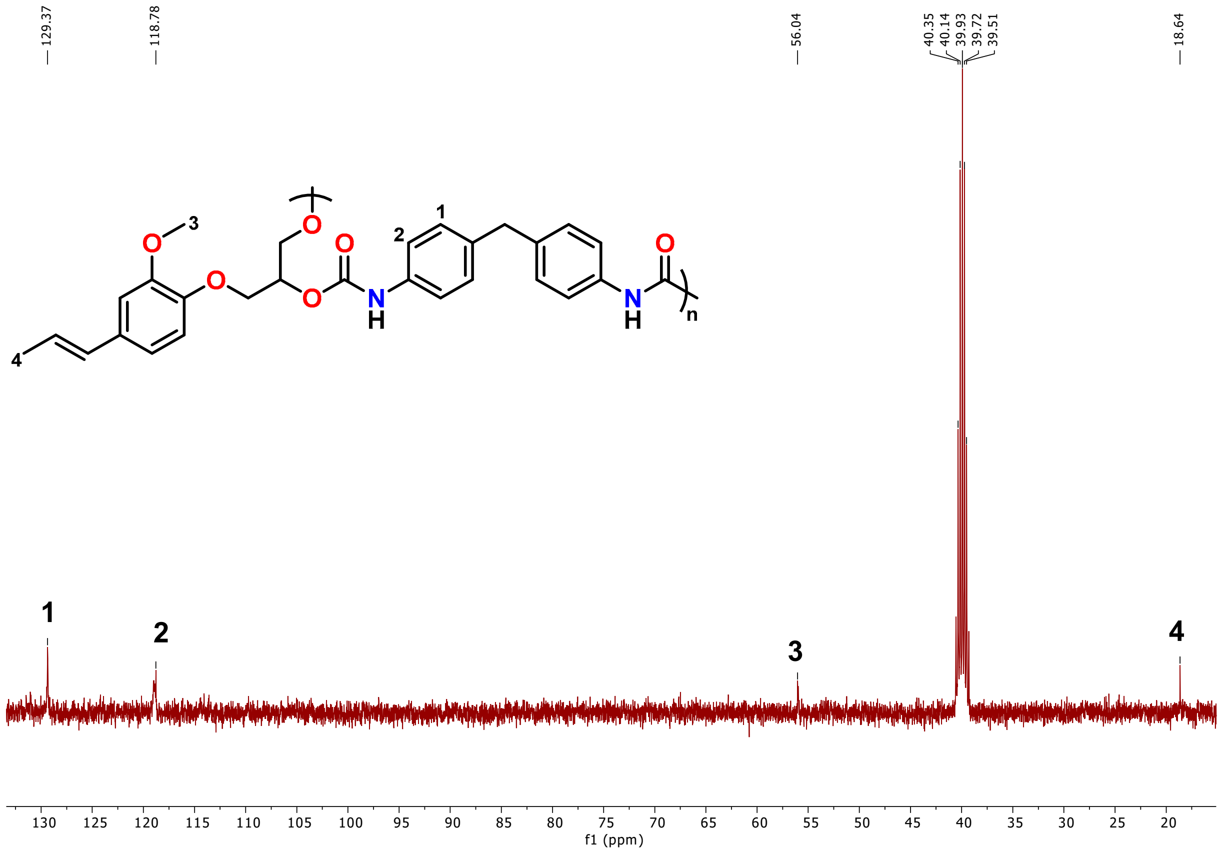

Figure S21: ^{13}C NMR of **PU-4a** in DMSO-d_6

^{13}C NMR (DMSO-d_6): δ = 129.37 (s, C1), 118.78 (s, C2), 56.04 (s, C3), 18.64 (s, C4) ppm (other carbon signals not observed)

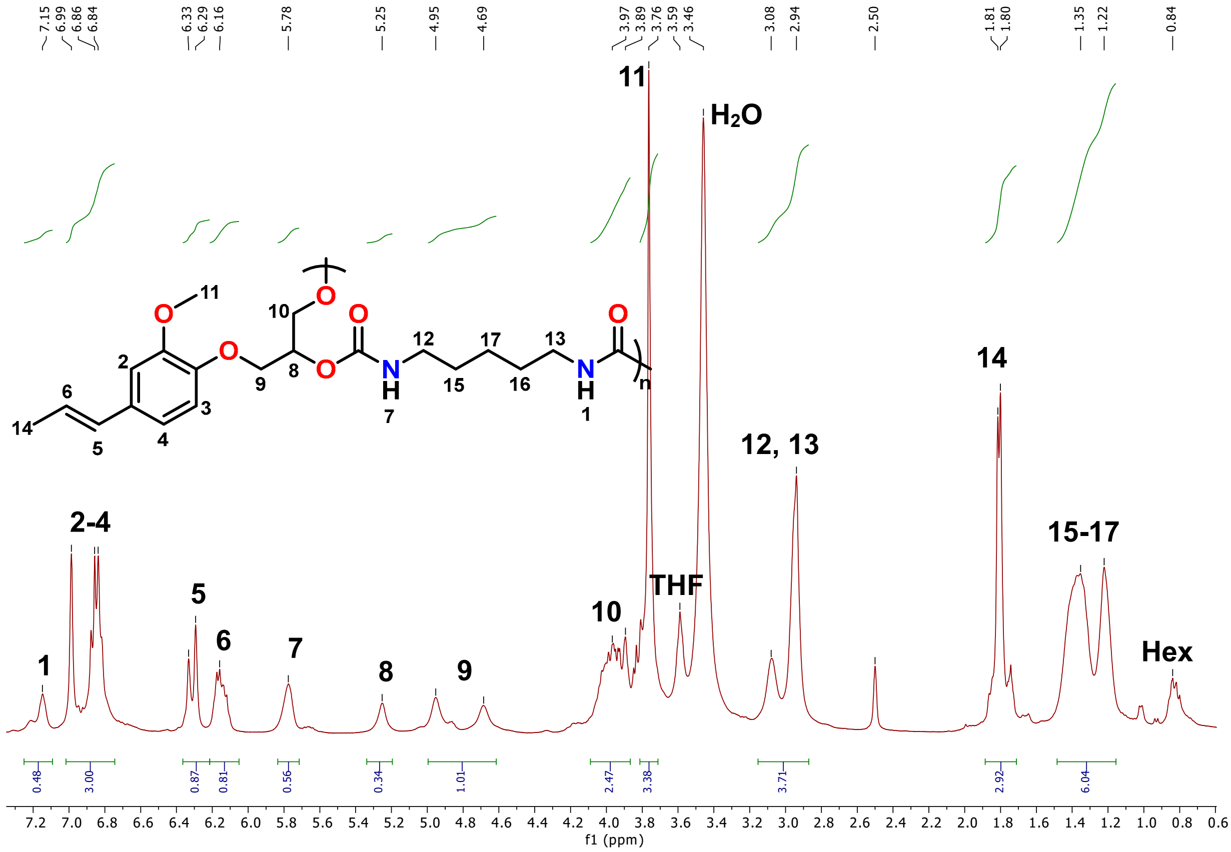

Figure S22: ¹H NMR of **PU-4b** in DMSO-d6

¹H NMR (DMSO-d6): δ = 7.15 (br s, 1H, H1), 6.84-6.99 (br m, 3H, H2, H3 and H4), 6.29-6.33 (d, 1H, H5), 6.16 (m, 1H, H6), 5.78 (br s, 1H, H7), 5.25 (br s, 1H, H8), 4.69-4.95 (br d, 1H, H9), 3.89-3.97 (m, 3H, H10), 3.76 (br s, 3H, H11), 2.94-3.08 (br d, 4H, H12 and H13), 1.80-1.81 (d, 3H, H14), 1.22-1.35 (br d, 6H, H15, H16 and H17)

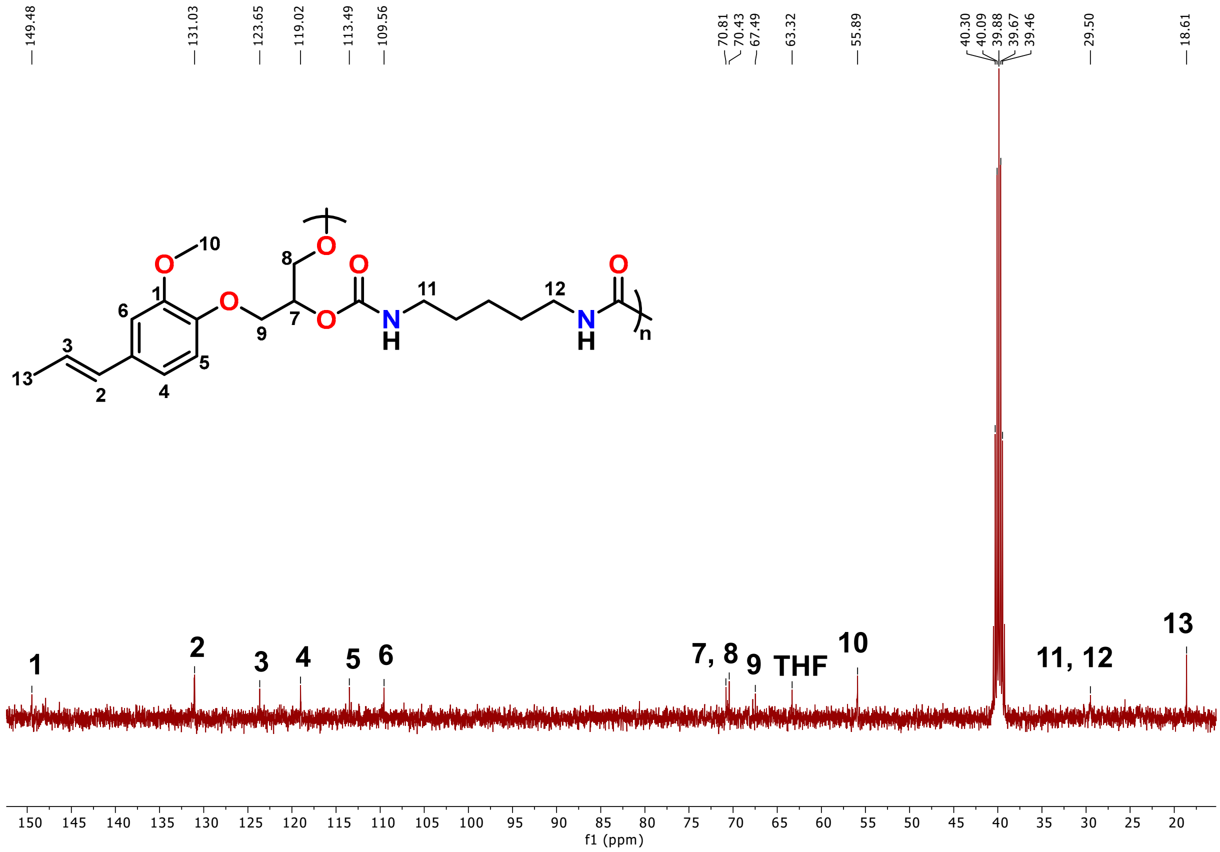

Figure S23: ^{13}C NMR of **PU-4b** in DMSO-d_6

^{13}C NMR (DMSO-d_6): δ = 149.48 (s, C1), 131.03 (s, C2), 123.65 (s, C3), 119.02 (s, C4), 113.49 (s, C5), 109.56 (s, C6), 70.81 (s, C7), 70.43 (s, C8), 67.49 (s, C9), 55.89 (s, C10), 29.50 (s, C11 and C12), 18.61 (s, C13) ppm (other carbon signals not observed)

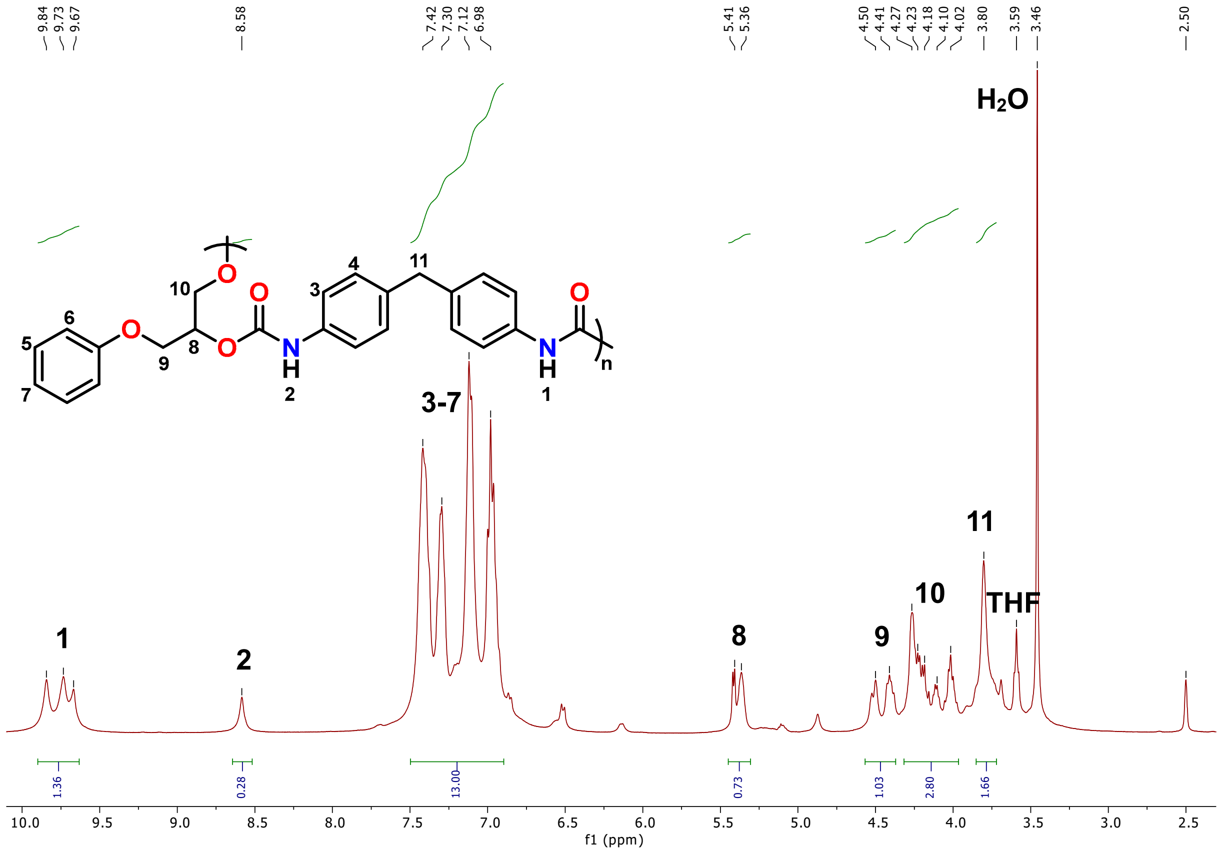

Figure S24: ${}^{1}H$ NMR of **PU-5a** in DMSO-d_6

^{1}H NMR (DMSO-d_6): δ = 9.67-9.84 (br t, 1H, H1), 8.58 (br s, 1H, H2), 6.98-7.42 (br m, 13H, H3, H4, H5, H6 and H7), 5.36-5.41 (br d, 1H, H8), 4.41-4.50 (br d, 1H, H9), 4.02-4.27 (br m, 3H, H10), 3.80 (br s, 2H, H11) ppm

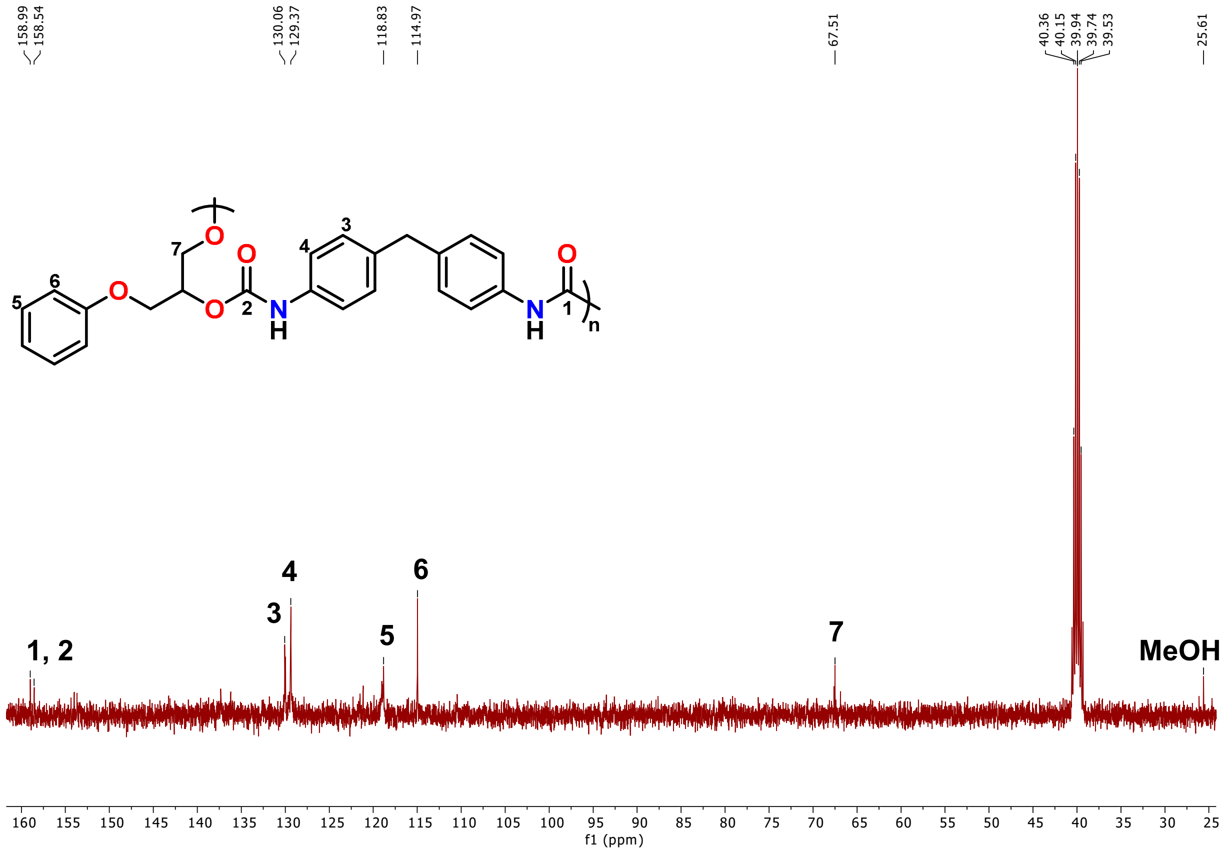

Figure S25: ^{13}C NMR of **PU-5a** in DMSO-d$_6$

^{13}C NMR (DMSO-d$_6$): δ = 158.99 (s, C1), 158.54 (s, C2), 130.06 (s, C3), 129.37 (s, C4), 118.83 (s, C5), 114.97 (s, C6), 67.51 (s, C7) ppm (other carbon signals not observed)

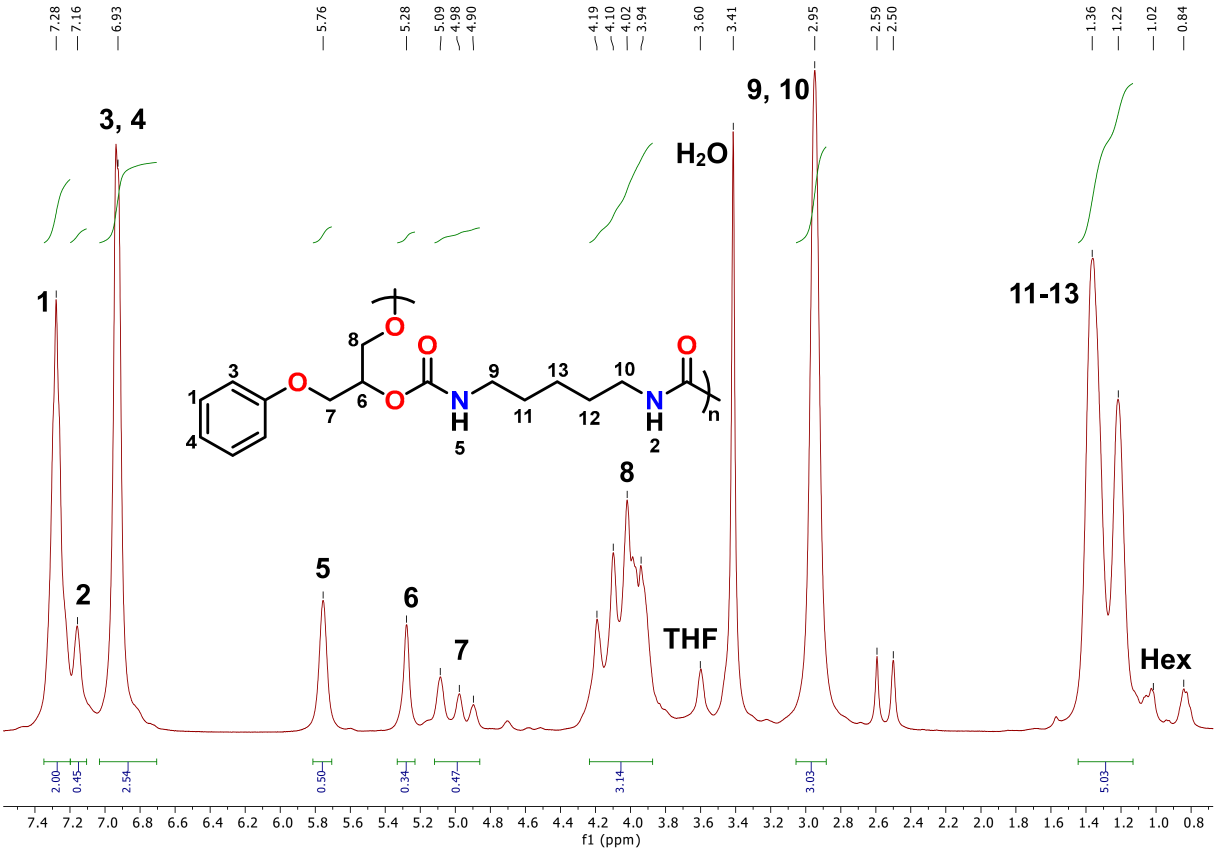

Figure S26: ^{1}H NMR of **PU-5b** in DMSO-d_6

^{1}H NMR (DMSO-d_6): δ = 7.28 (br s, 2H, H1), 7.16 (br s, 1H, H2), 6.93 (br s, 3H, H3 and H4), 5.76 (br s, 1H, H5), 5.28 (br s, 1H, H6), 4.90-5.09 (br t, 1H, H7), 3.94-4.19 (br m, 3H, H8), 2.95 (br s, 3H, H9 and H10), 1.22-1.36 (br d, 5H, H11, H12 and H13) ppm

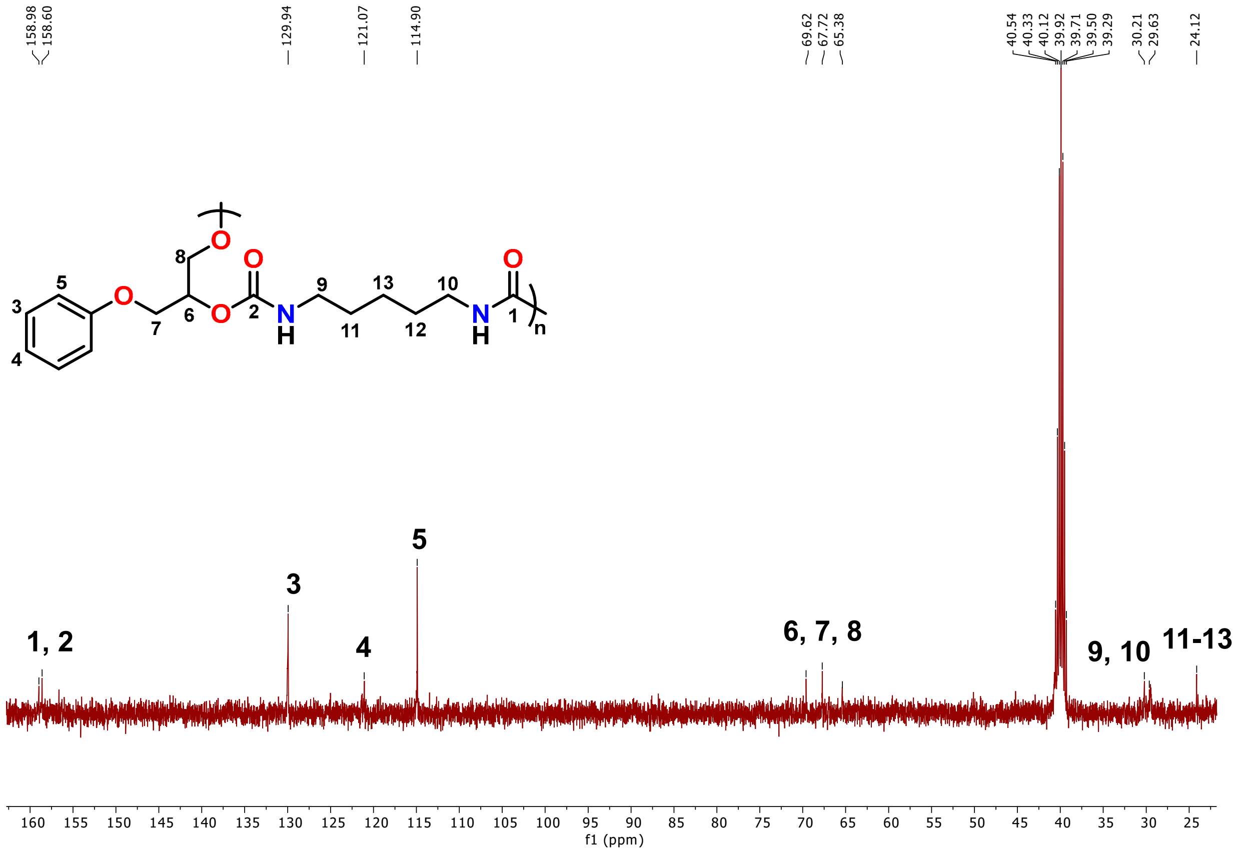

Figure S27: ^{13}C NMR of **PU-5b** in DMSO-d_6

^{13}C NMR (DMSO-d_6): δ = 158.98 (s, C1), 158.60 (s, C2), 129.94 (s, C3), 121.07 (s, C4), 114.90 (s, C5), 69.62 (s, C6), 67.72 (s, C7), 65.38 (s, C8), 30.21 (s, C9), 29.63 (s, C10), 24.12 (s, C11, C12 and C13) ppm

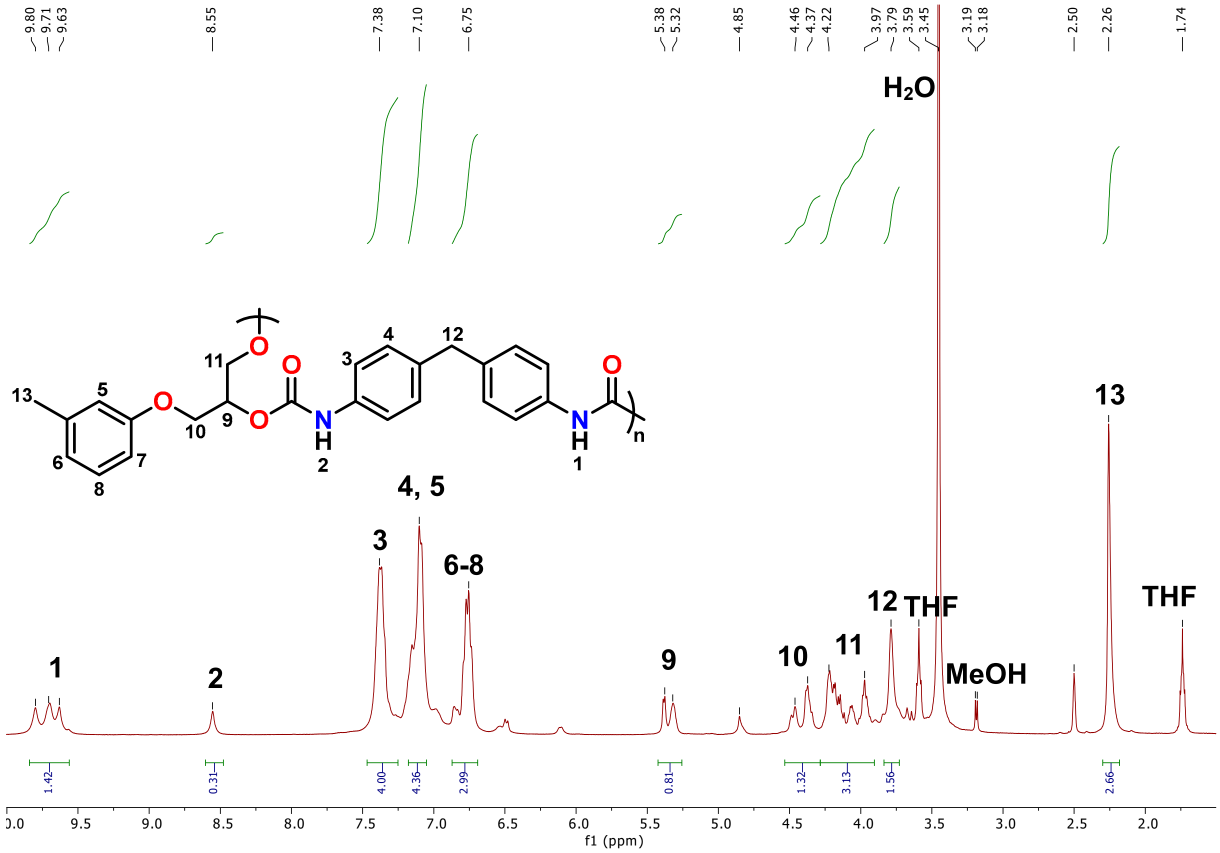

Figure S28: ^{1}H NMR of **PU-6a** in DMSO-d_6

^{1}H NMR (DMSO-d_6): δ = 9.63-9.80 (br t, 1H, H1), 8.55 (br s, 1H, H2), 7.38 (br s, 4H, H3), 7.10 (br s, 4H, H4 and H5), 6.75 (br m, 3H, H6, H7 and H8), 5.32-5.38 (br d, 1H, H9), 4.37-4.46 (br d, 1H, H10), 3.97-4.22 (br m, 3H, H11), 3.79 (br s, 2H, H12), 2.26 (br s, 3H, H13) ppm

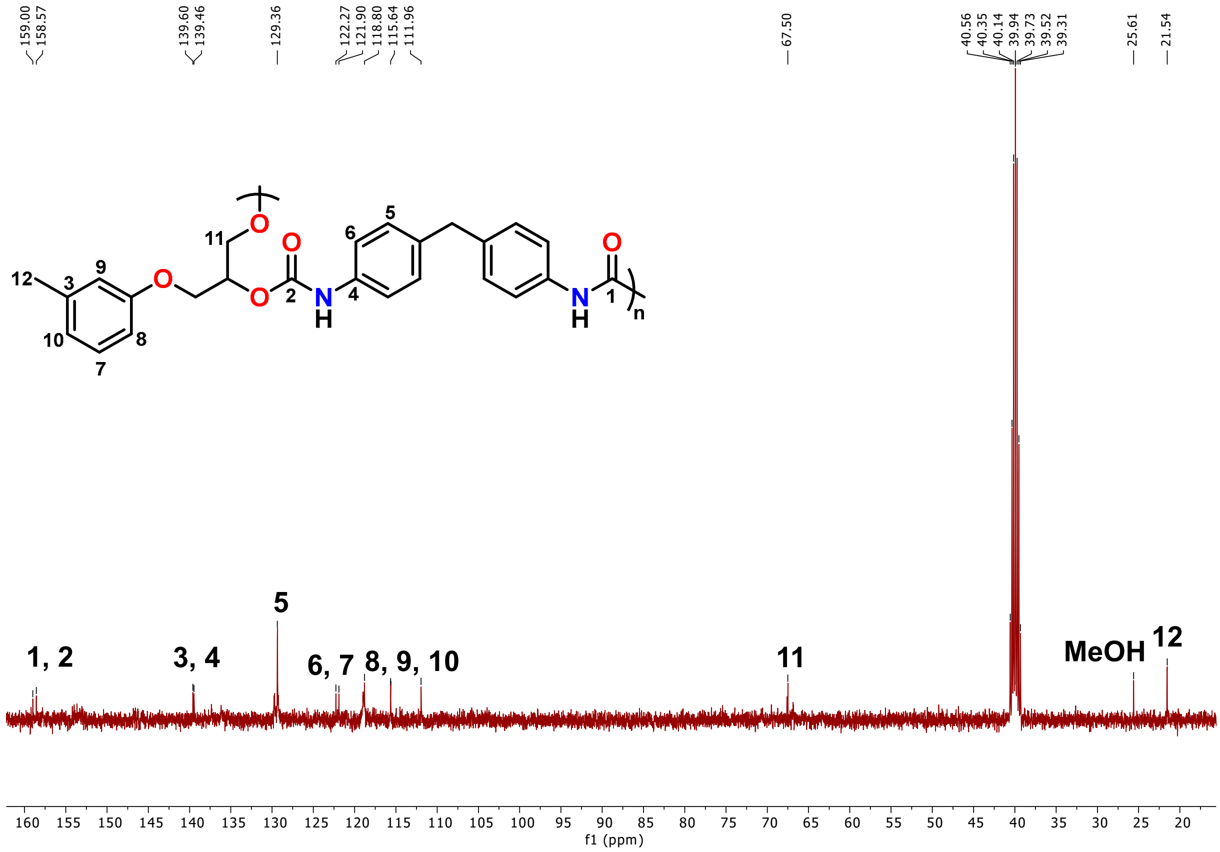

Figure S29: ^{13}C NMR of **PU-6a** in DMSO-d$_6$

^{13}C NMR (DMSO-d$_6$): δ = 159.00 (s, C1), 158.57 (s, C2), 139.60 (s, C3), 139.46 (s, C4), 129.36 (s, C5), 122.27 (s, C6), 121.90 (s, C7), 118.80 (s, C8), 115.64 (s, C9), 111.96 (s, C10), 67.50 (s, C11), 21.54 (s, C12) ppm (other carbon signals not observed)

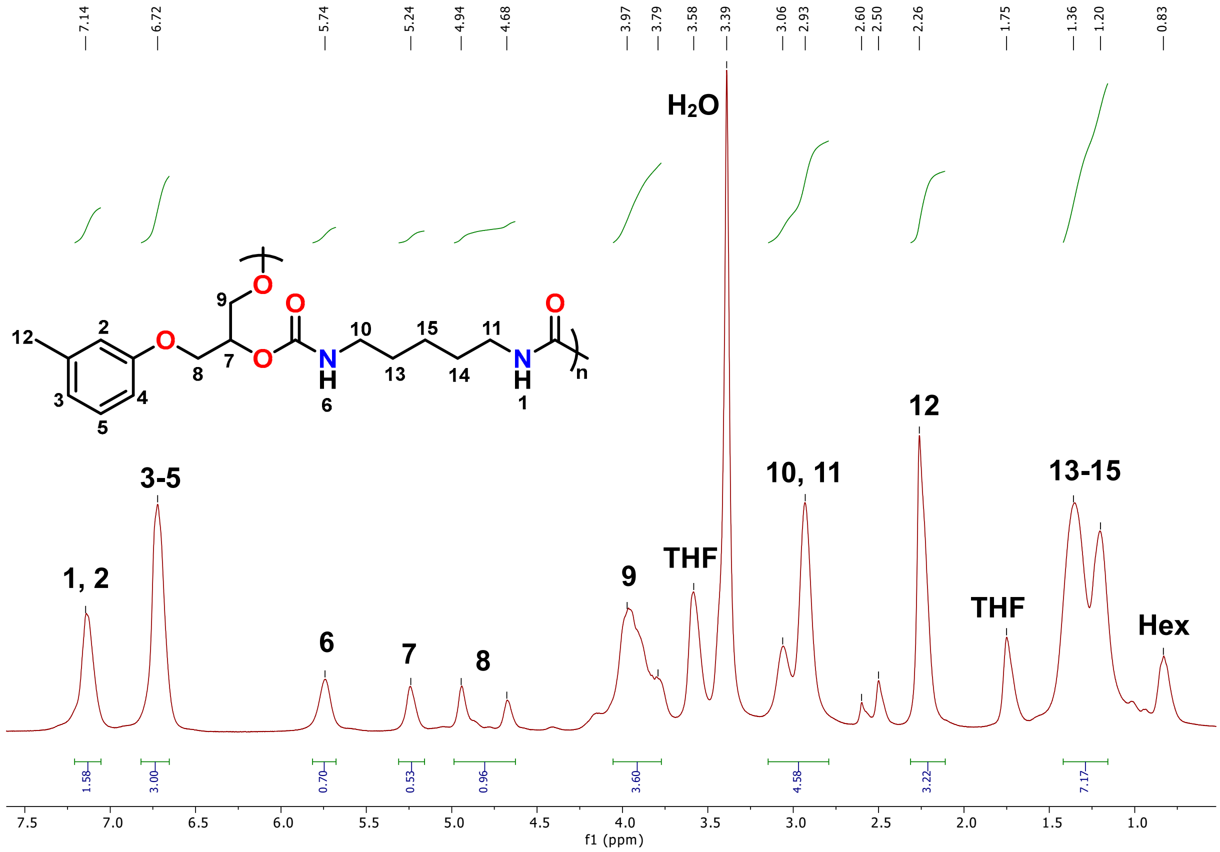

Figure S30: ^{1}H NMR of **PU-6b** in DMSO-d6

^{1}H NMR (DMSO-d6): δ = 7.14 (br s, 2H, H1 and H2), 6.72 (br s, 3H, H3, H4 and H5), 5.74 (br s, 1H, H6), 5.24 (br s, 1H, H7), 4.68-4.94 (br d, 1H, H8), 3.79-3.97 (br m, 4H, H9), 2.93-3.06 (br d, 5H, H10 and H11), 2.26 (br s, 3H, H12), 1.20-1.36 (br d, 7H, H13, H14 and H15) ppm

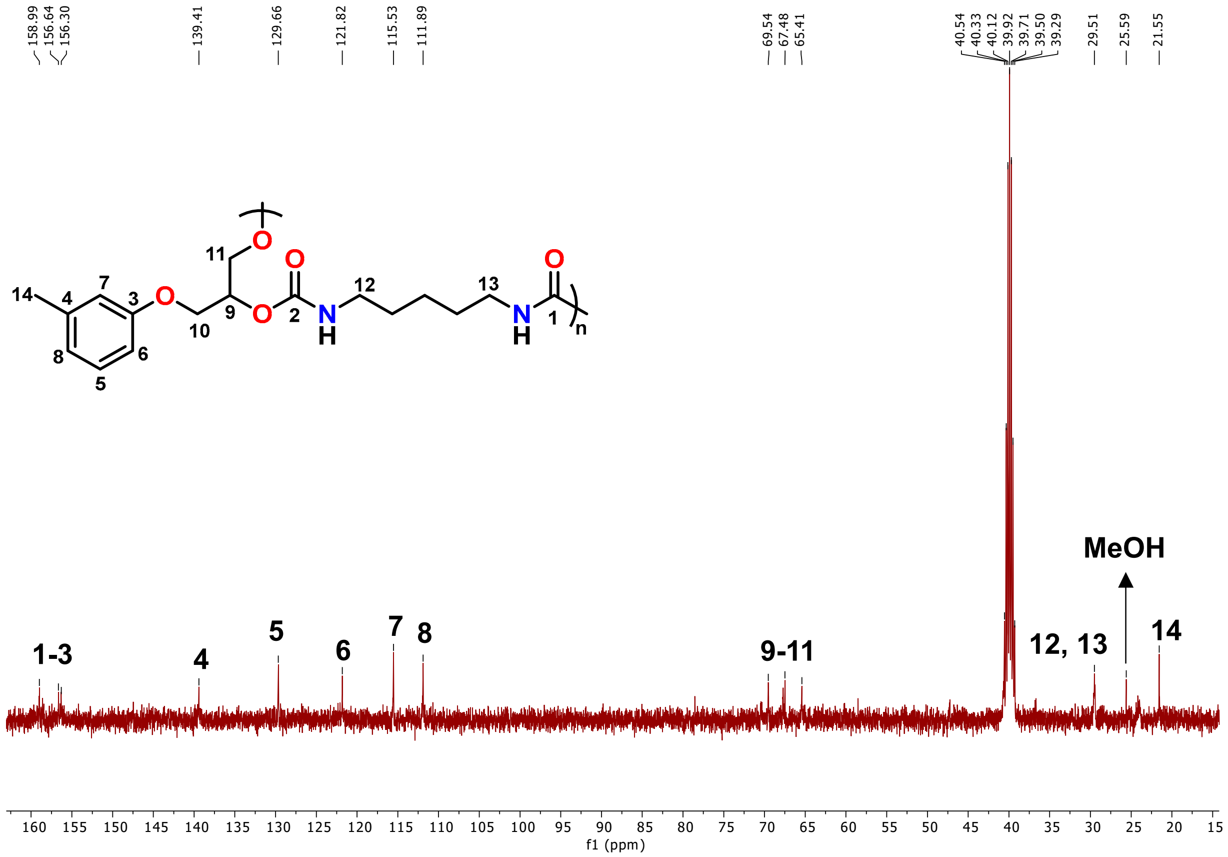

Figure S31: ^{13}C NMR of **PU-6b** in DMSO-d$_6$

^{13}C NMR (DMSO-d$_6$): δ = 158.99 (s, C1), 156.64 (s, C2), 156.30 (s, C3), 139.41 (s, C4), 129.66 (s, C5), 121.82 (s, C6), 115.53 (s, C7), 111.89 (s, C8), 69.54 (s, C9), 67.48 (s, C10), 65.41 (s, C11), 29.51 (s, C12 and C13), 21.55 (s, C14) ppm (other carbon signals not observed)

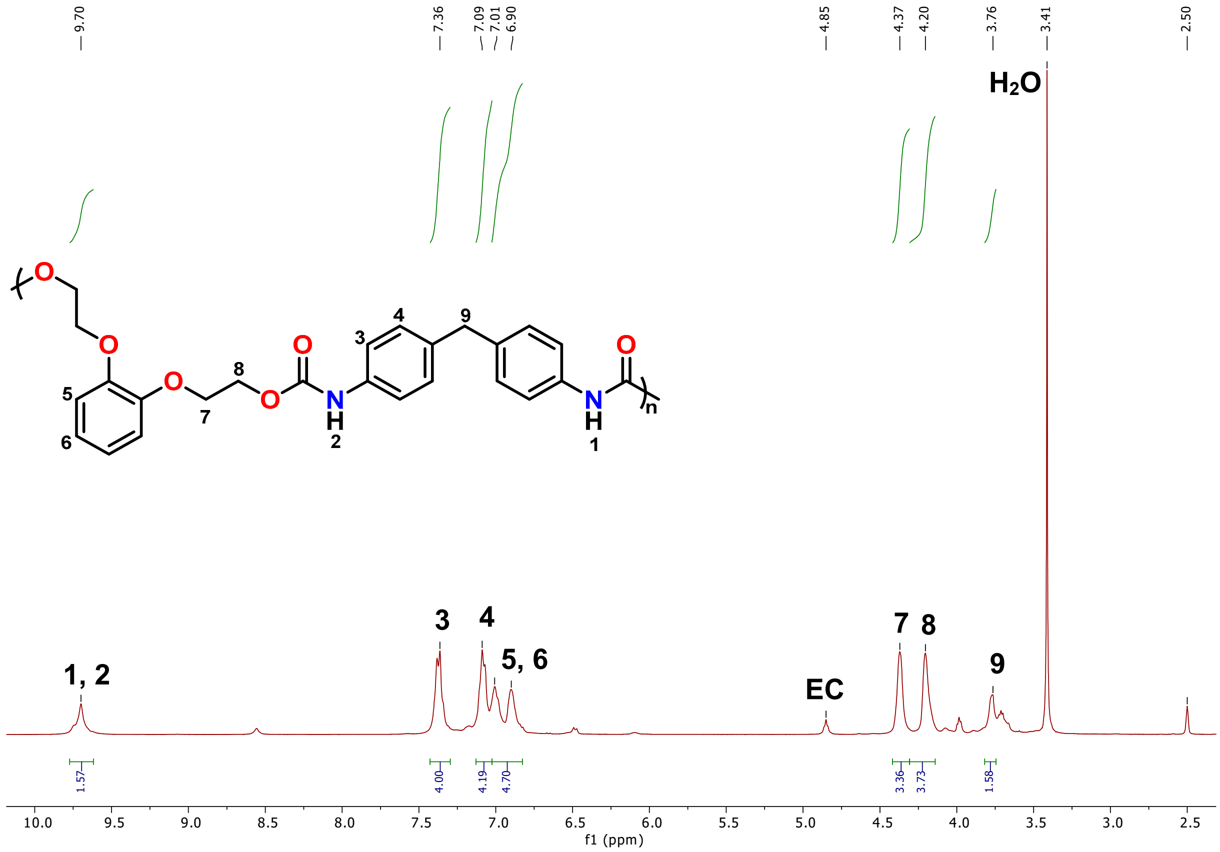

Figure S32: ^{1}H NMR of **PU-7a** in DMSO-d$_6$

^{1}H NMR (DMSO-d$_6$): δ = 9.70 (br s, 2H, H1 and H2), 7.36 (br d, 4H, H3), 7.09 (br s, 4H, H4), 6.90-7.01 (br d, 5H, H5 and H6), 4.37 (br s, 3H, H7), 4.20 (br s, 4H, H8), 3.76 (br s, 2H, H9) ppm

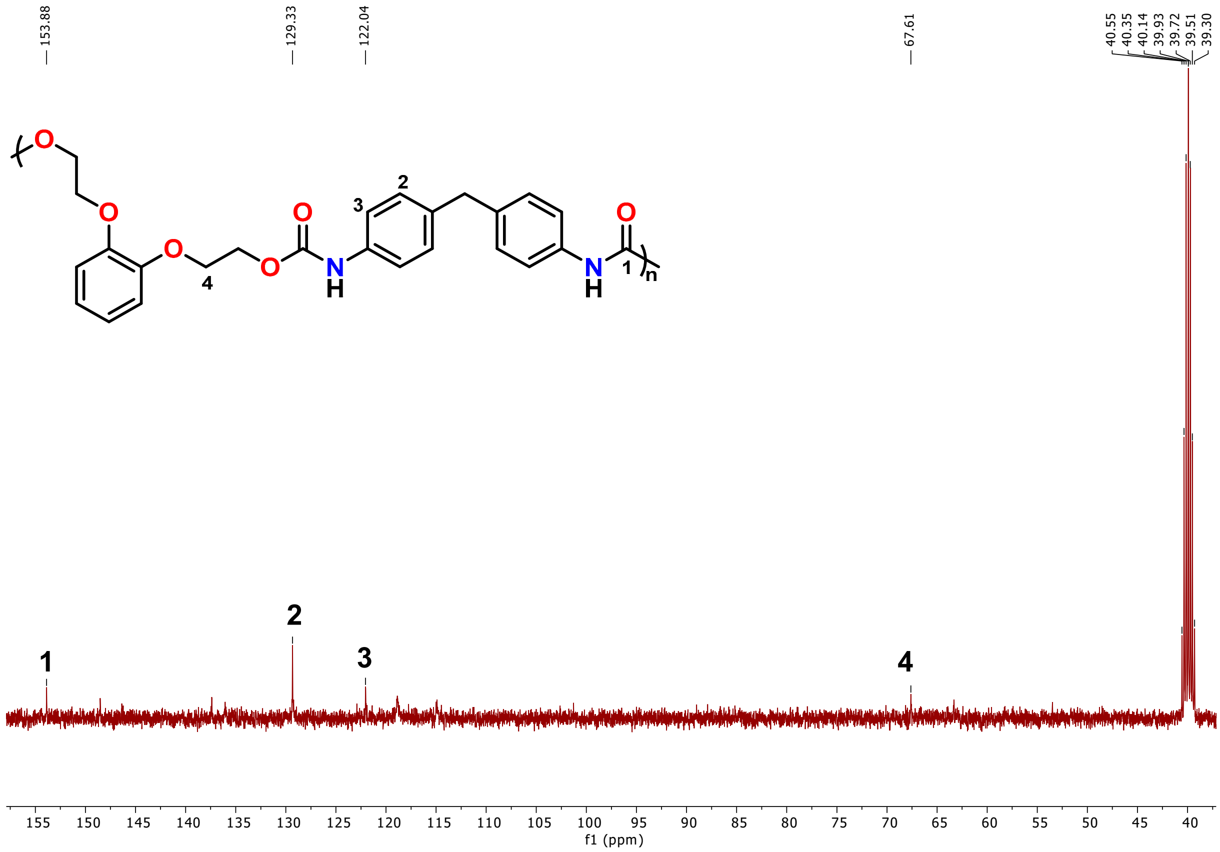

Figure S33: ^{13}C NMR of **PU-7a** in DMSO-d_6

^{13}C NMR (DMSO-d_6): δ = 153.88 (s, C1), 129.33 (s, C2), 122.04 (s, C3), 67.61 (s, C4) ppm (other carbon signals not observed)

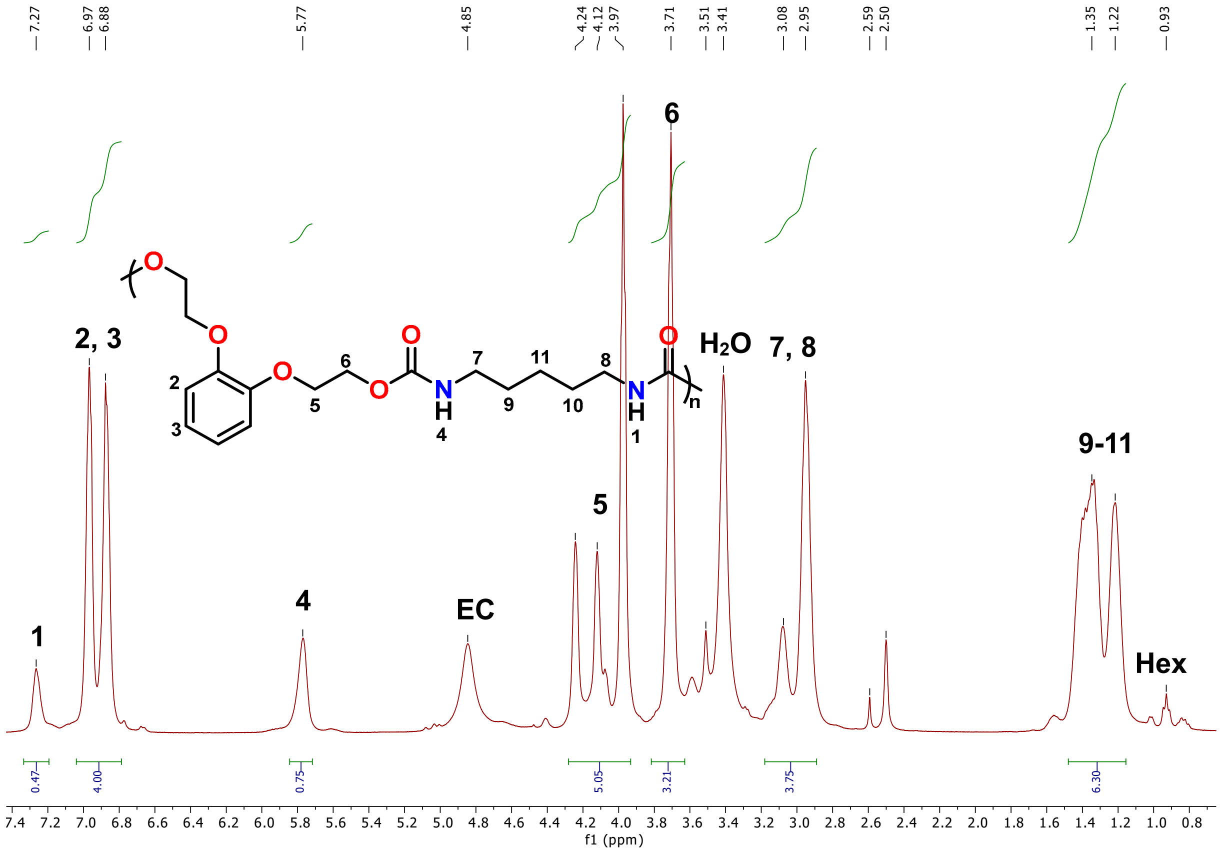

Figure S34: ^{1}H NMR of **PU-7b** in DMSO-d6

^{1}H NMR (DMSO-d6): δ = 7.27 (br s, 1H, H1), 6.88-6.97 (br d, 4H, H2 and H3), 5.77 (br s, 1H, H4), 4.12-4.24 (br d, 2H, H5), 3.97-4.24 (br t, 5H, H5), 3.71 (br s, 3H, H6), 2.95-3.08 (br d, 4H, H7 and H8), 1.22-1.35 (br d, 6H, H9, H10 and H11) ppm

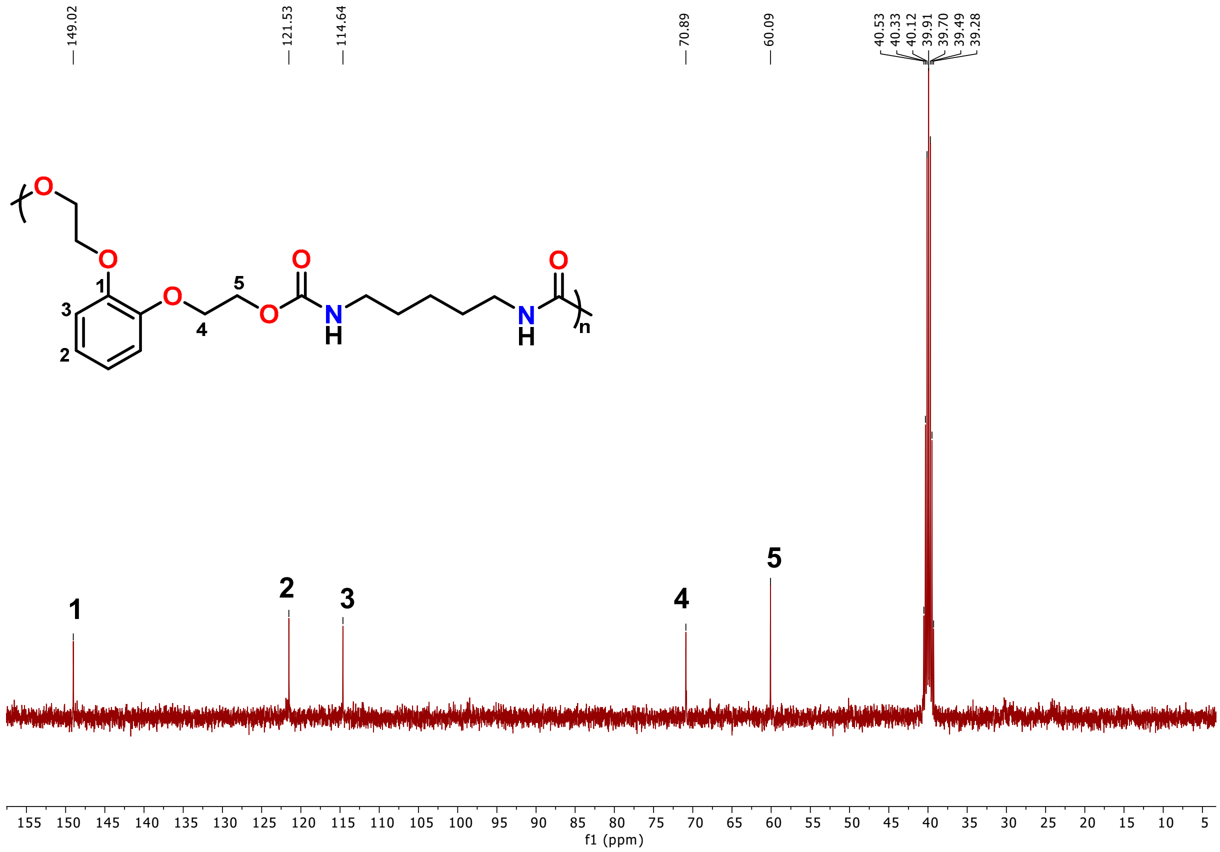

Figure S35: ^{13}C NMR of **PU-7b** in DMSO-d$_6$

^{13}C NMR (DMSO-d$_6$): δ = 149.02 (s, C1), 121.53 (s, C2), 114.64 (s, C3), 70.89 (s, C4), 60.09 (s, C5) ppm (other carbon signals not observed)

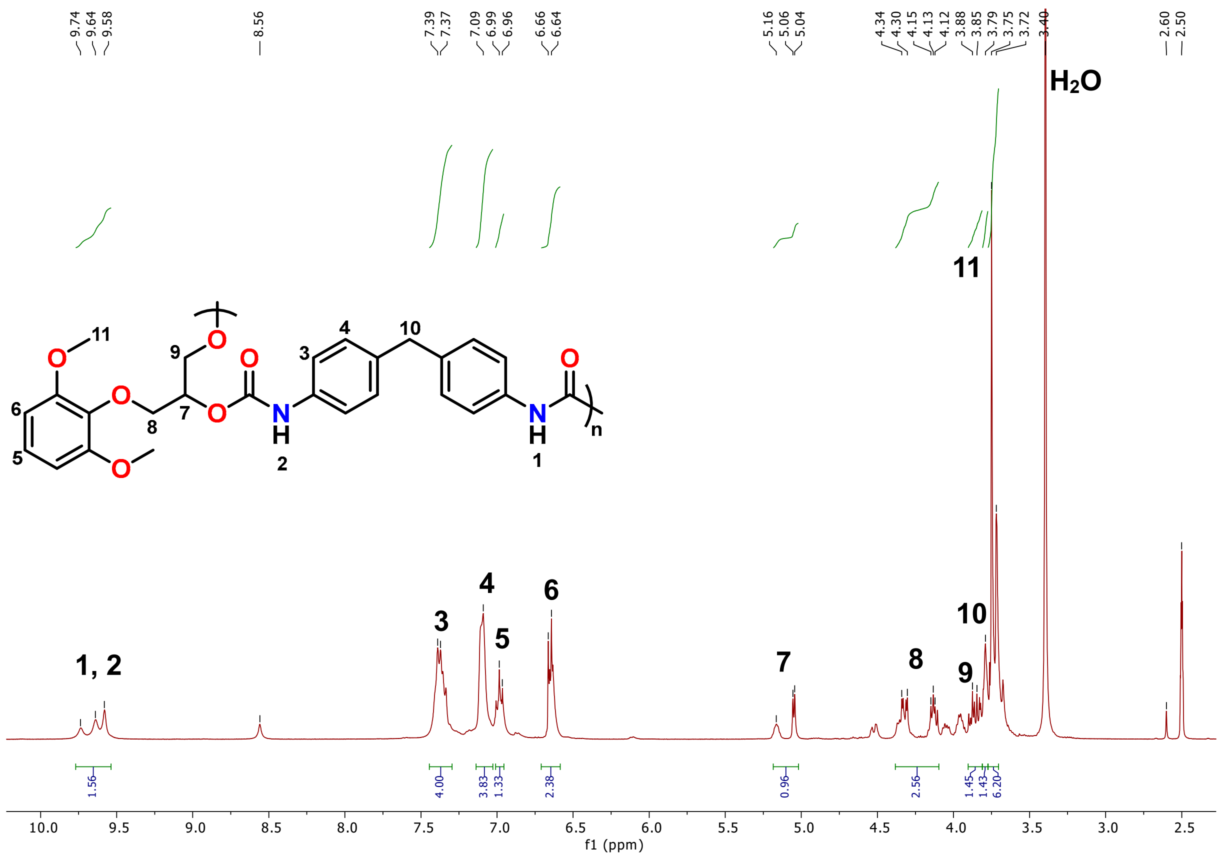

Figure S36: ^{1}H NMR of **PU-8a** in DMSO-d_6

^{1}H NMR (DMSO-d_6): δ = 9.58-9.74 (br t, 2H, H1 and H2), 7.37-7.39 (m, 4H, H3), 7.09 (br s, 4H, H4), 6.96-6.99 (m, 1H, H5), 6.64-6.66 (br d, 2H, H6), 5.04-5.16 (br m, 1H, H7), 4.12-4.34 (br m, 3H, H8), 3.85-3.88 (m, 1H, H9), 3.79 (br s, 1H, H10), 3.72-3.75 (d, 6H, H11) ppm

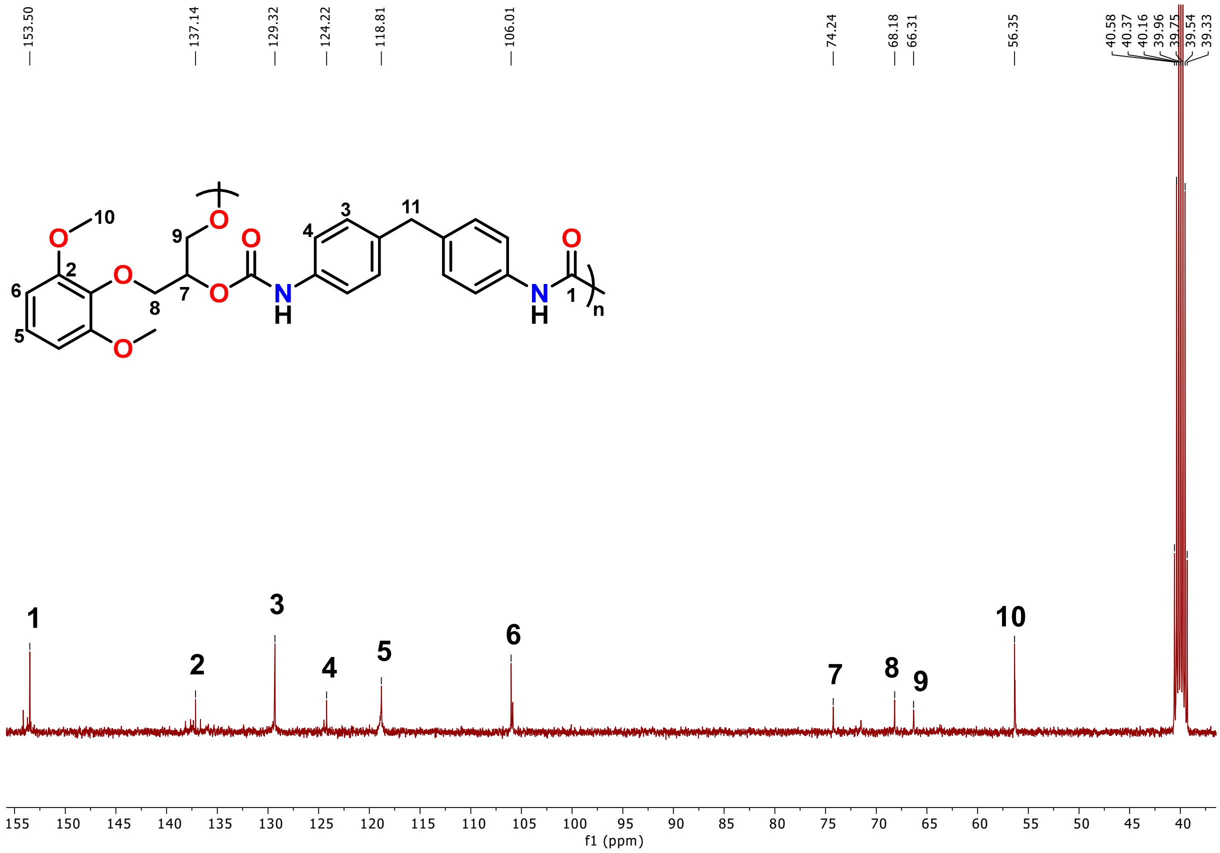

Figure S37: ^{13}C NMR of **PU-8a** in DMSO-d$_6$

^{13}C NMR (DMSO-d$_6$): δ = 153.50 (s, C1), 137.14 (s, C2), 129.32 (s, C3), 124.22 (s, C4), 118.81 (s, C5), 106.01 (s, C6), 74.24 (s, C7), 68.18 (s, C8), 66.31 (s, C9), 56.25 (s, C10) ppm (C11 not observed)

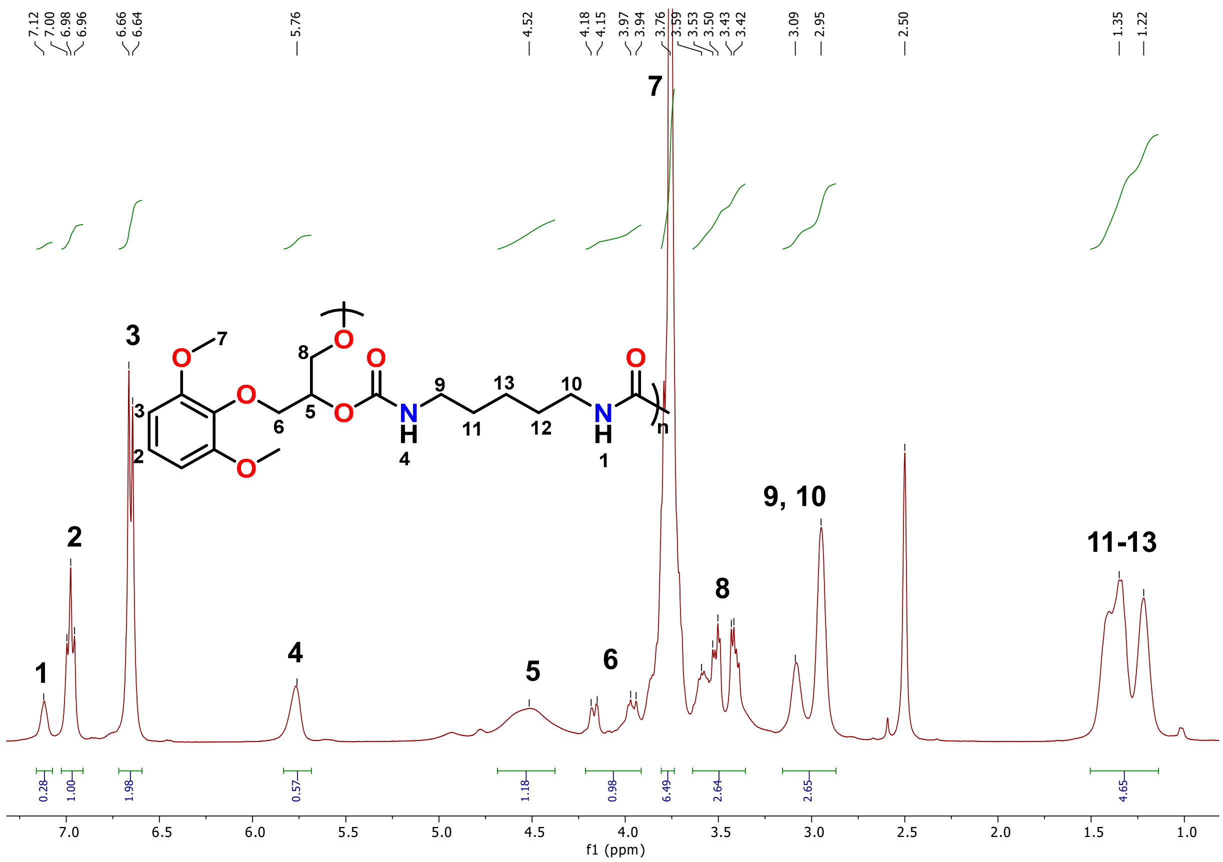

Figure S38: ^{1}H NMR of **PU-8b** in DMSO-d6

^{1}H NMR (DMSO-d6): δ = 7.12 (br s, 1H, H1), 6.96-7.00 (t, 1H, H2), 6.64-6.66 (d, 2H, H3), 5.76 (br s, 1H, H4), 4.52 (br s, 1H, H5), 3.94-4.18 (br m, 1H, H6), 3.79 (br s, 6H, H7), 3.42-3.59 (br m, 3H, H8), 2.95-3.09 (br d, 3H, H9 and H10), 1.22-1.35 (br m, 5H, H11, H12 and H13) ppm

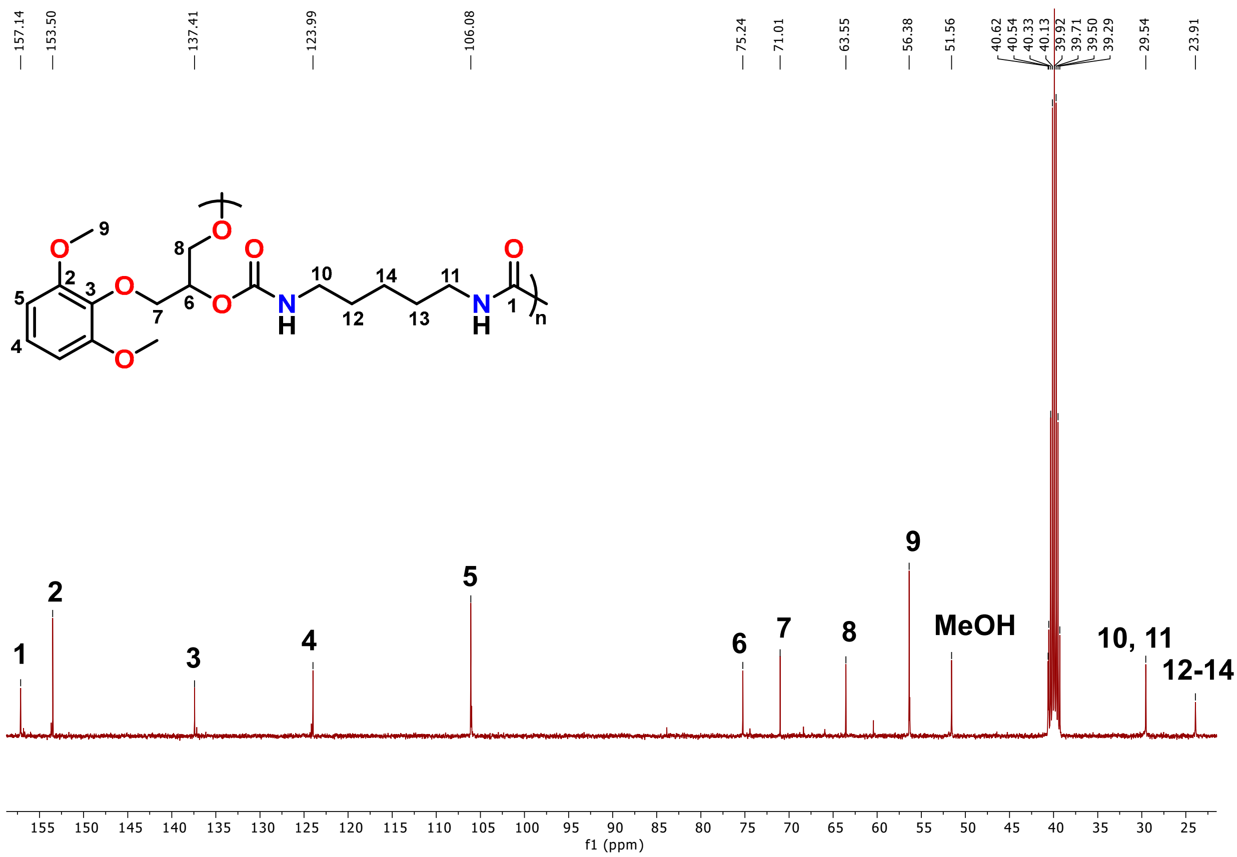

Figure S39: ^{13}C NMR of **PU-8b** in DMSO-d$_6$

^{13}C NMR (DMSO-d$_6$): δ = 157.14 (s, C1), 153.50 (s, C2), 137.41 (s, C3), 123.99 (s, C4), 106.08 (s, C5), 75.24 (s, C6), 71.01 (s, C7), 63.55 (s, C8), 56.38 (s, C9), 29.54 (s, C10 and C11), 23.91 (s, C12, C13 and C14) ppm

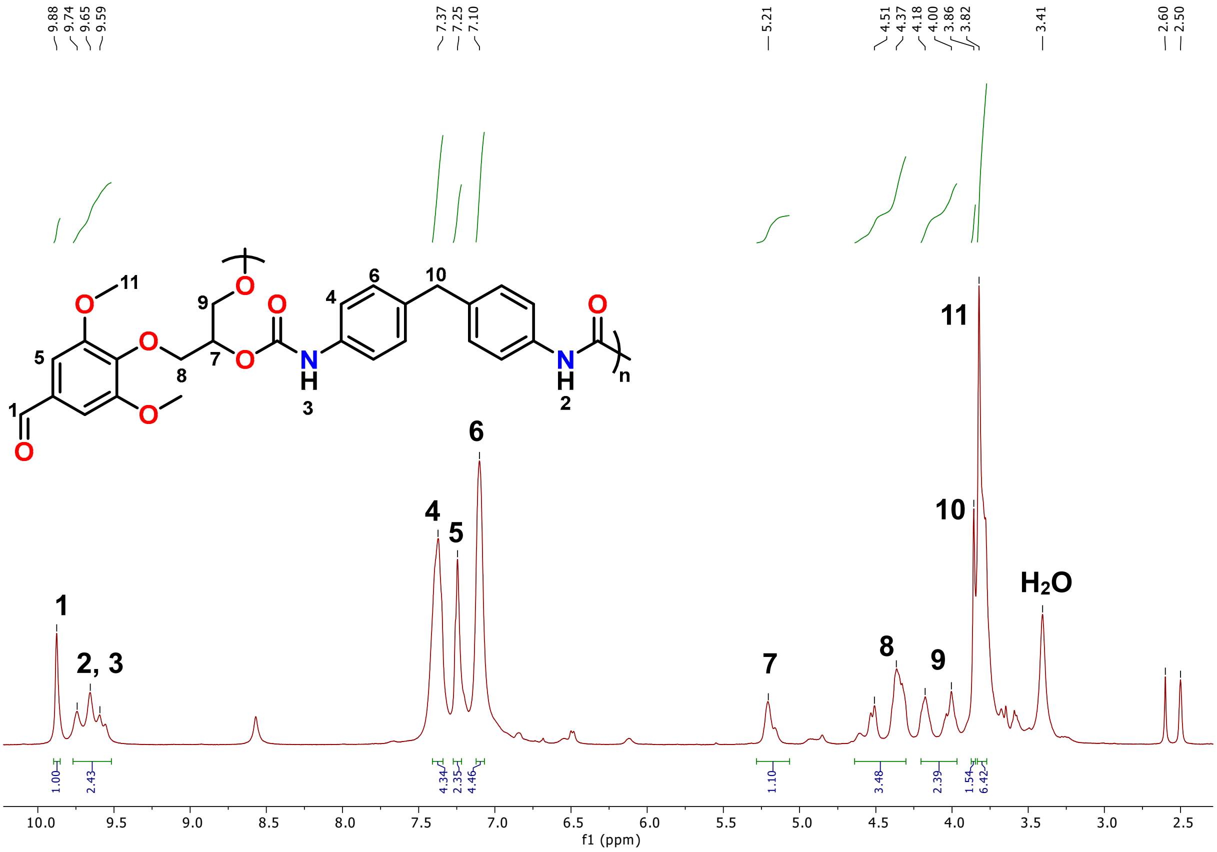

Figure S40: ^{1}H NMR of **PU-9a** in DMSO-d$_6$

^{1}H NMR (DMSO-d$_6$): δ = 9.88 (br s, 1H, H1), 9.59-9.74 (br t, 2H, H2 and H3), 7.37 (br s, 4H, H4), 7.25 (br s, 2H, H5), 7.10 (br s, 4H, H6), 5.21 (br m, 1H, H7), 4.37-4.51 (br m, 3H, H8), 4.00-4.18 (br d, 2H, H9), 3.86 (br s, 2H, H10), 3.82 (br s, 6H, H11) ppm

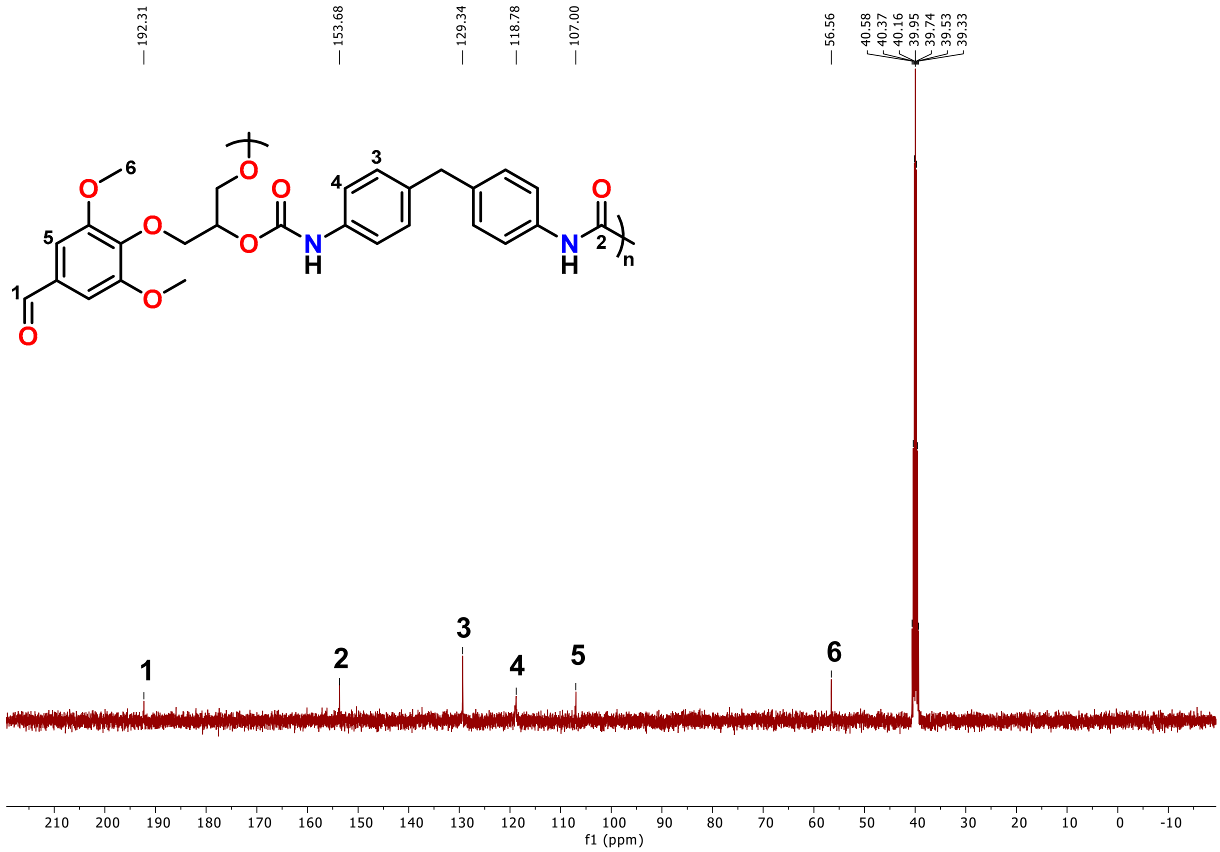

Figure S41: ^{13}C NMR of **PU-9a** in DMSO-d_6

^{13}C NMR (DMSO-d_6): δ = 192.31 (s, C1), 153.68 (s, C2), 129.34 (s, C3), 118.78 (s, C4), 107.00 (s, C5), 56.56 (s, C6) ppm (other carbon signals not observed)

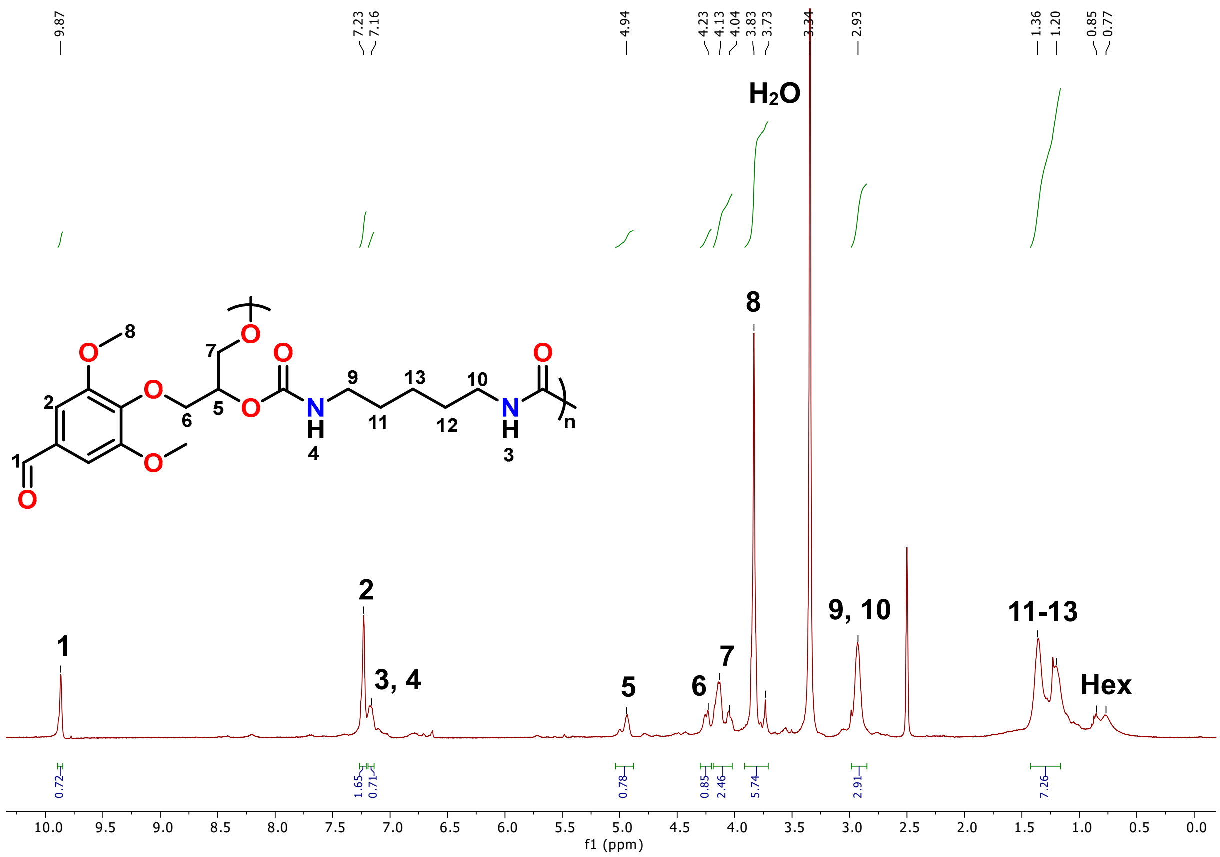

Figure S42: ^{1}H NMR of **PU-9b** in DMSO-d$_6$

^{1}H NMR (DMSO-d$_6$): δ = 9.87 (br s, 1H, H1), 7.23 (br s, 2H, H2), 7.16 (br s, 1H, H3 and H4), 4.94 (br s, 1H, H5), 4.23 (br s, 1H, H6), 4.04-4.13 (br m, 2H, H7), 3.73-3.83 (br d, 6H, H8), 2.93 (br s, 3H, H9 and H10), 1.20-1.36 (br d, 7H, H11, H12 and H13) ppm

Polyurethane FTIR Data

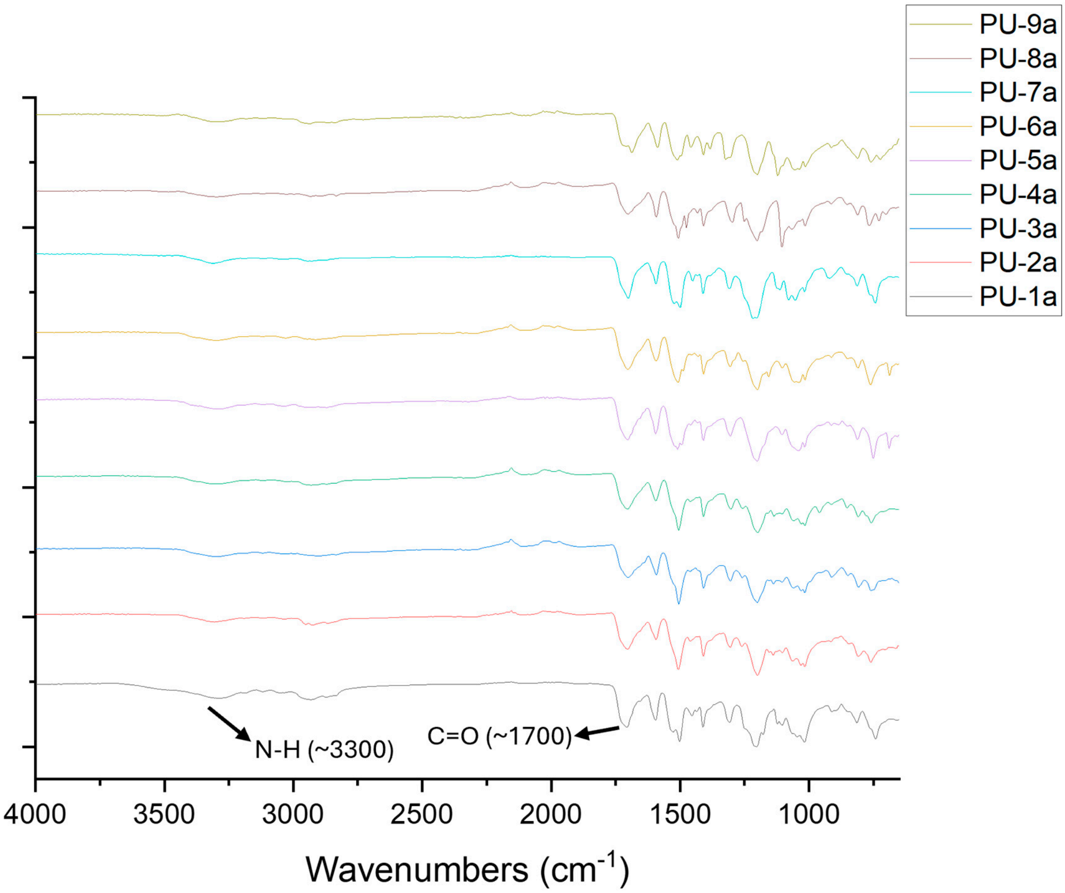

Figure S43: FTIR analysis of **PU-1a** to **PU-9a**

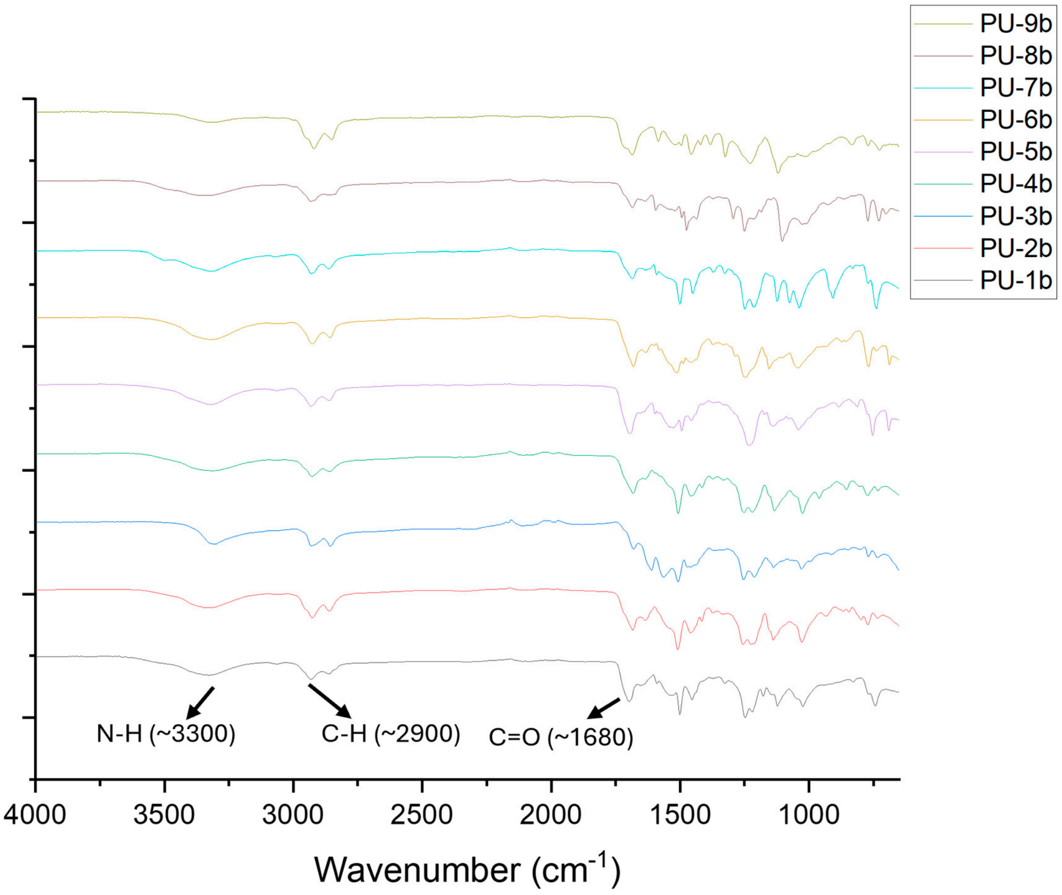

Figure S44: FTIR analysis of **PU-1b** to **PU-9b**

Polyurethane TGA Data

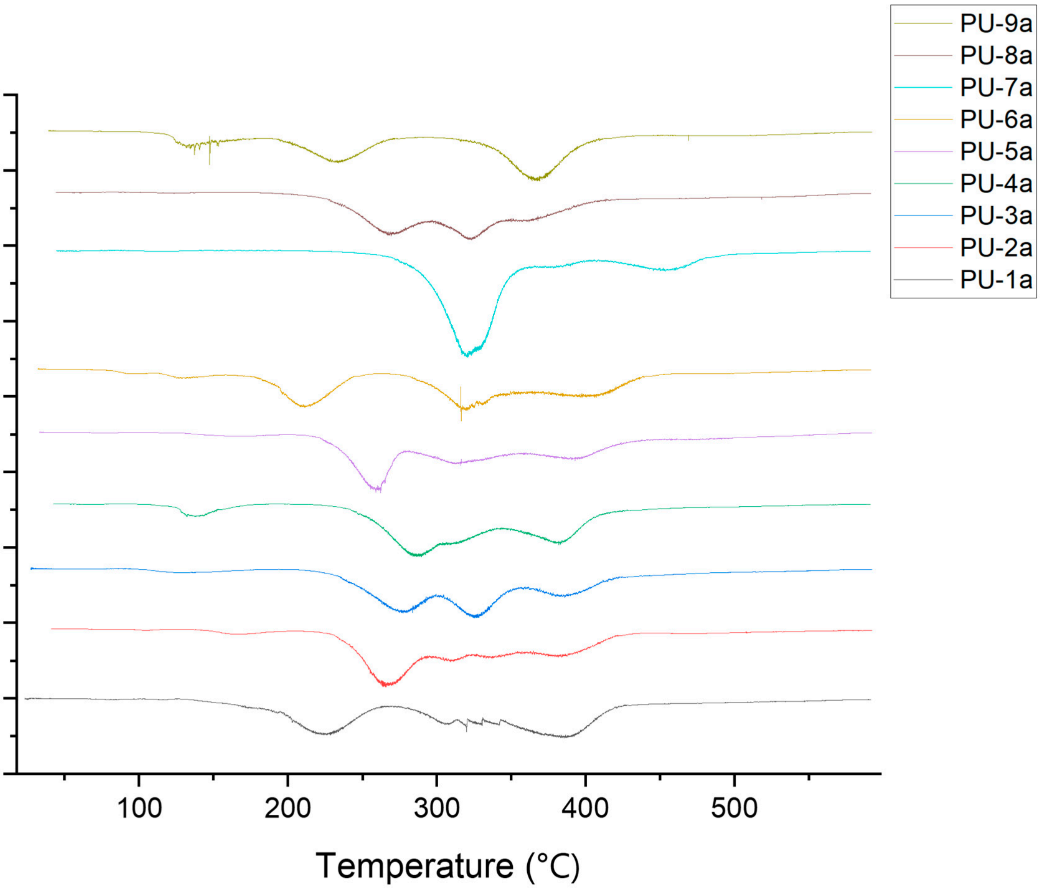

Figure S45: Derivative Weight Change of **PU-1a** to **PU-9a**

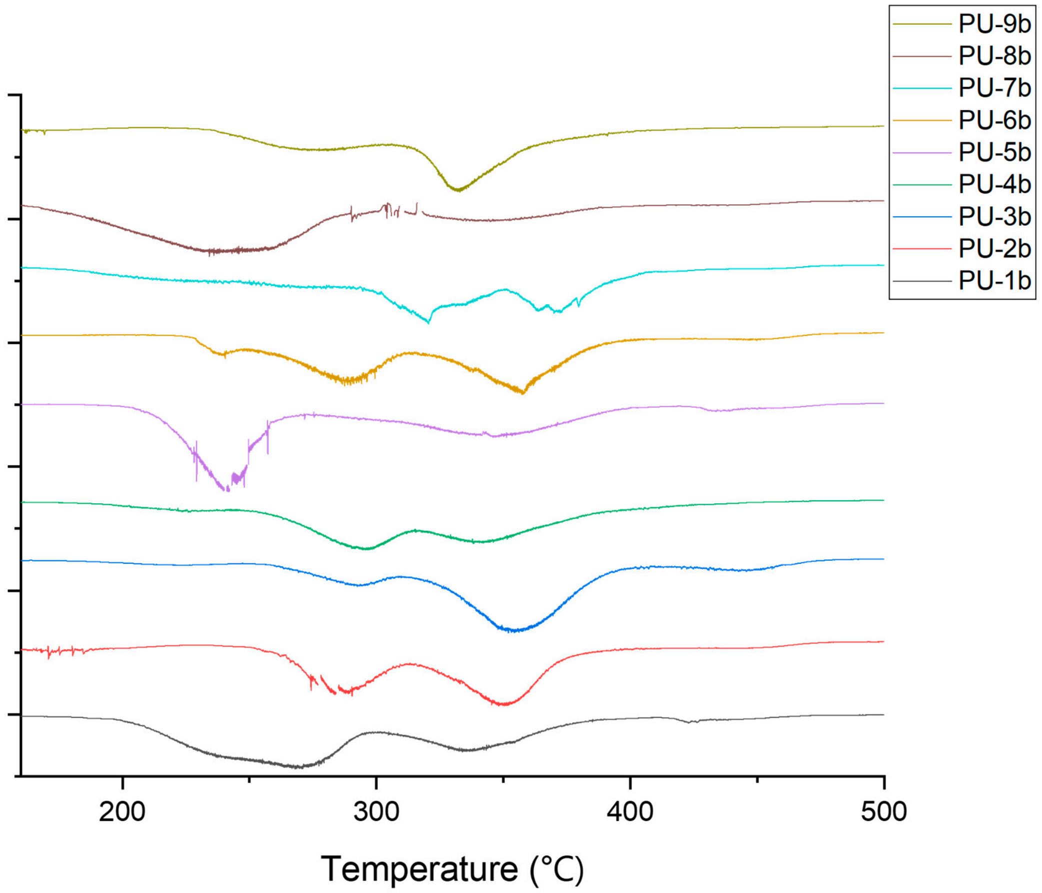

Figure S46: Derivative Weight Change of **PU-1b** to **PU-9b**

Polyester NMRs

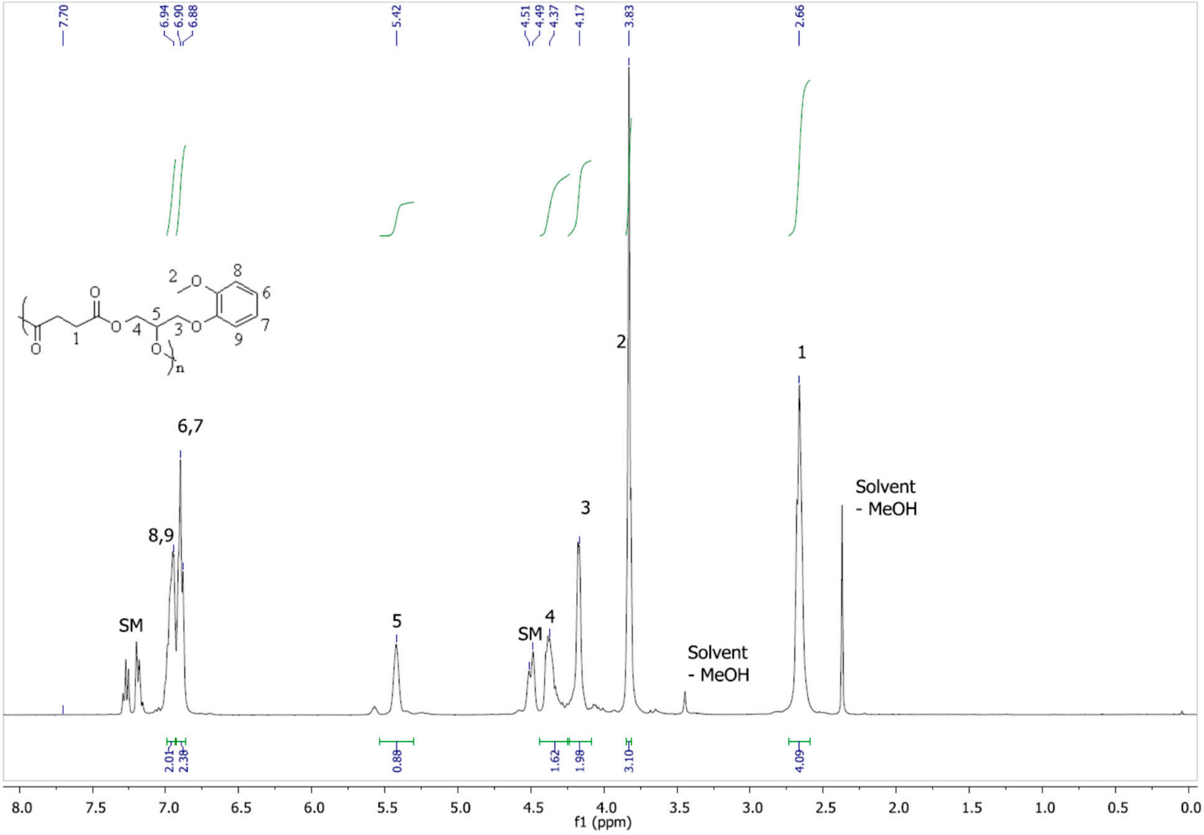

Figure S47: ^{1}H NMR of **PE-1a** in $CDCl_3$

^{1}H NMR (400 MHz, $CDCl_3$): δ = 6.94 (m, 2H, H8 and H9), 6.90 (m, 2H, H7 and H8), 5.42 (m, 1H, H6), 4.50 (m, 1H, H5), 4.37 (m, 1H, H4), 4.17 (m, 2H, H3), 3.83 (s, 3H, H2), 2.66 (s, 4H, H1) ppm

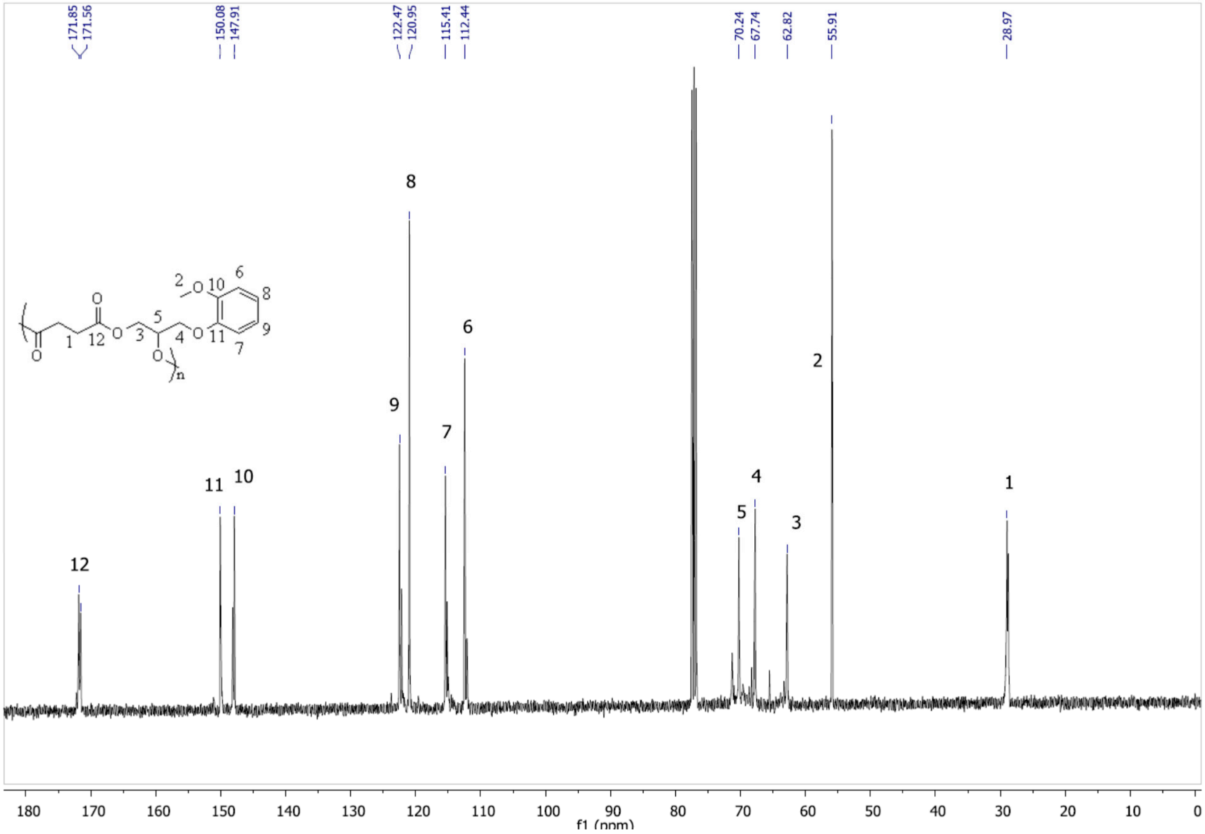

Figure S48: ^{13}C NMR of **PE-1a** in $CDCl_3$

$^{13}C\{^1H\}$ NMR (101 MHz, $CDCl_3$): δ = 171.85 (s, C13), 171.56 (s, C12), 150.08 (s, C11), (C10) (s, C10), 122.50 (s, C9), 120.96 (s, C8), 115.47 (s, C7), 112.49 (s, C6), 70.26 (s, C5), 67.79 (s, C4), 62.82 (s, C3), 55.91 (s, C2), 28.90 (s, C1) ppm

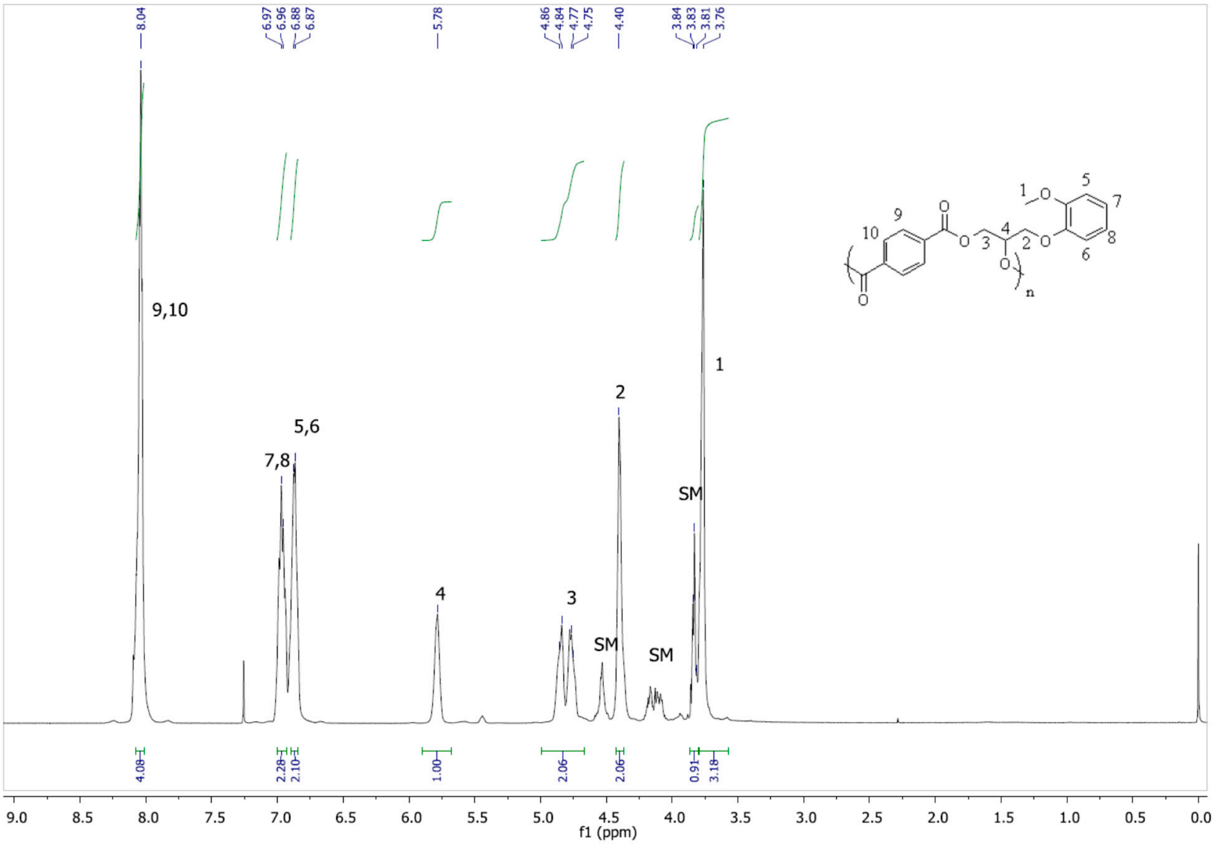

Figure S49: ^{1}H NMR of **PE-1b** in $CDCl_3$

^{1}H NMR (400 MHz, $CDCl_3$) δ = 8.68 (d, 1H, H13, $^{3}J_{HH}$ = 7.5 Hz), 8.19 (d, 2H, H12, $^{3}J_{HH}$ = 7.2 Hz), 7.46 (m, 1H, H11), 6.94 (m, 2H, H9 and H10), 6.86 (m, 2H, H7 and H8), 5.79 (m, 1H, H6), 4.85 (m, 1H, H5), 4.76 (m, 1H, H4), 4.39 (m, 2H, H2 and H3), 3.76 (s, 3H, H1) ppm

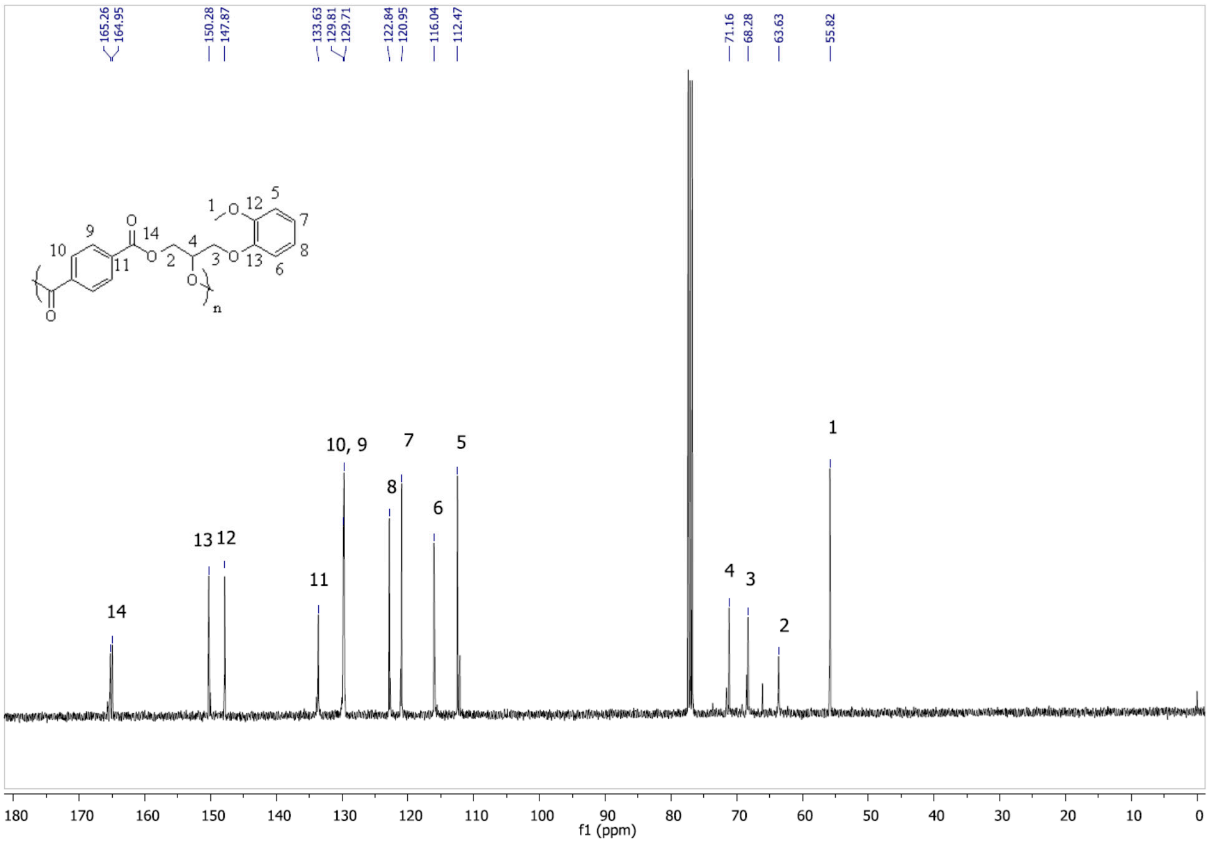

Figure S50: ^{13}C NMR of **PE-1b** in $CDCl_3$

$^{13}C\{^1H\}$ NMR (101 MHz, $CDCl_3$) δ = 165.20 (s, C18), 164.89 (s, C17), 150.23 (s, C16), 147.86 (s, C15), 134.19 (s, C13 and C14), 131.03 (s, C11 and C12), 130.20 (s, C10), 128.75 (s, C9), 122.71 (s, C8), 120.95 (s, C7), 115.95 (s, C6), 112.47 (s, C5), 71.08 (s, C4), 68.20 (s, C3), 63.63 (s, C2), 55.81 (s, C1) ppm

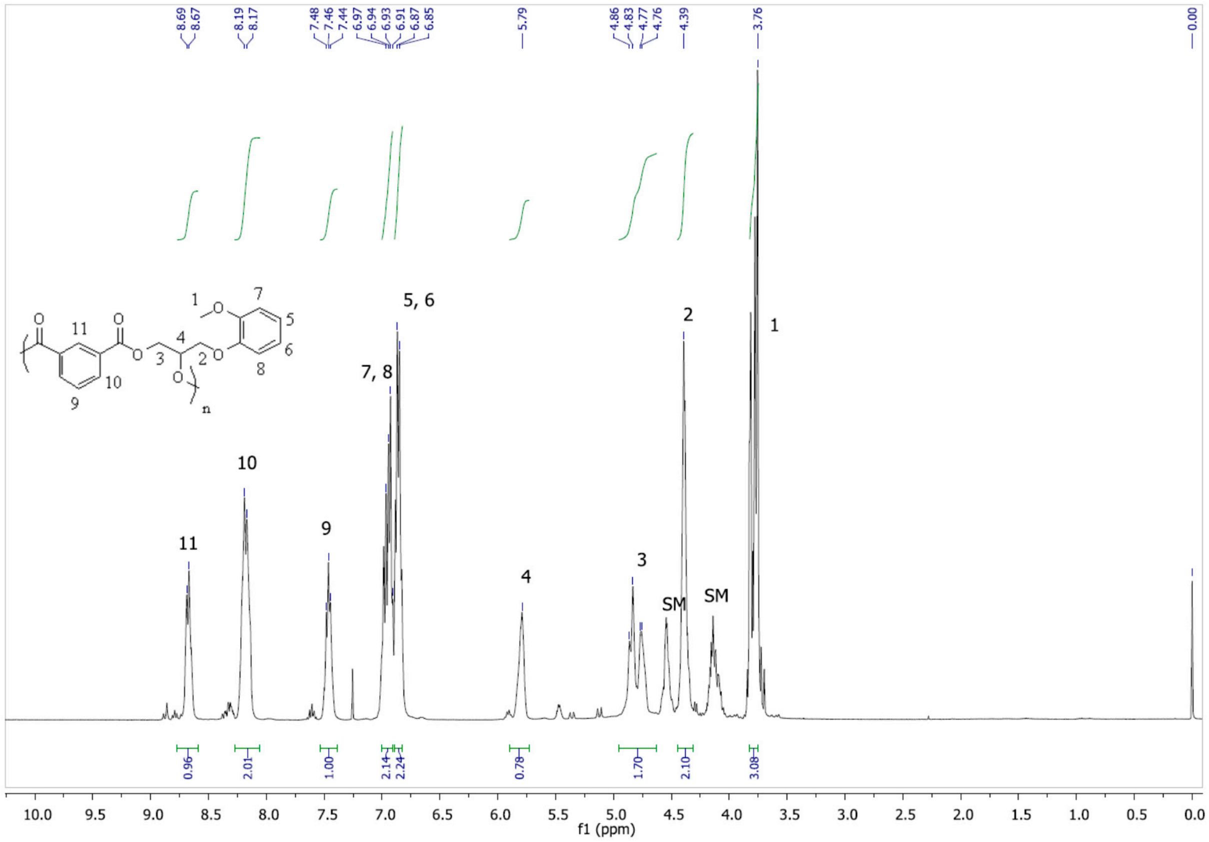

Figure S51: ^{1}H NMR of **PE-1c** in $CDCl_3$

^{1}H NMR (400 MHz, $CDCl_3$) δ = 8.04 (m, 4H, H8 and H9), 6.96 (m, 2H, H7), 6.87 (m, 2H, H6), 5.78 (m, 1H, H5), 4.85 (m, 1H, H4), 4.76 (m, 1H, H3), 4.40 (m, 2H, H2), 3.76 (s, 3H, H1) ppm

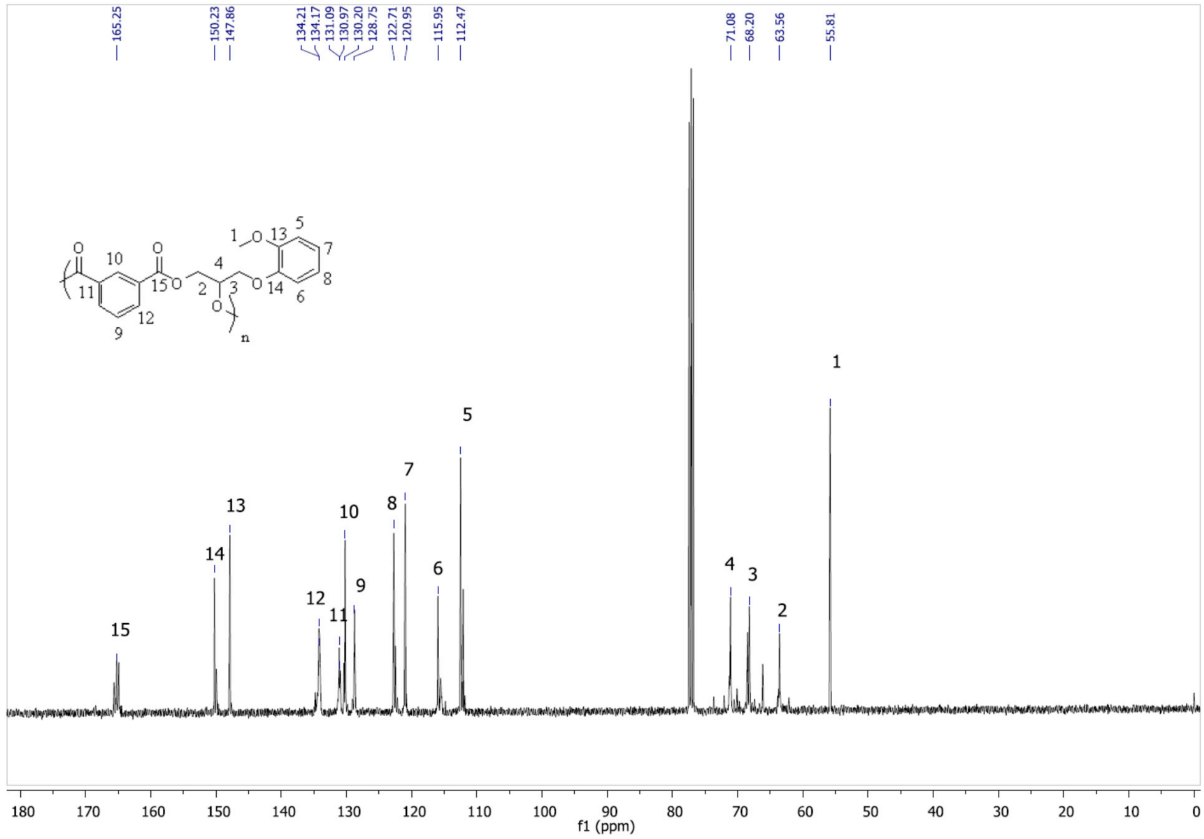

Figure S52: ^{13}C NMR of **PE-1c** in $CDCl_3$

^{13}C{^{1}H} NMR (101 MHz, $CDCl_3$) δ = 164.95 (s, C13), 150.28 (s, C12), 147.87 (s, C11), 133.63 (s, C10), 129.75 (s, C9), 122.84 (s, C8), 120.96 (s, C7), 116.04 (s, C6), 112.47 (s, C5), 71.16 (s, C4), 68.28 (s, C3), 63.63 (s, C2), 55.82 (s, C1) ppm

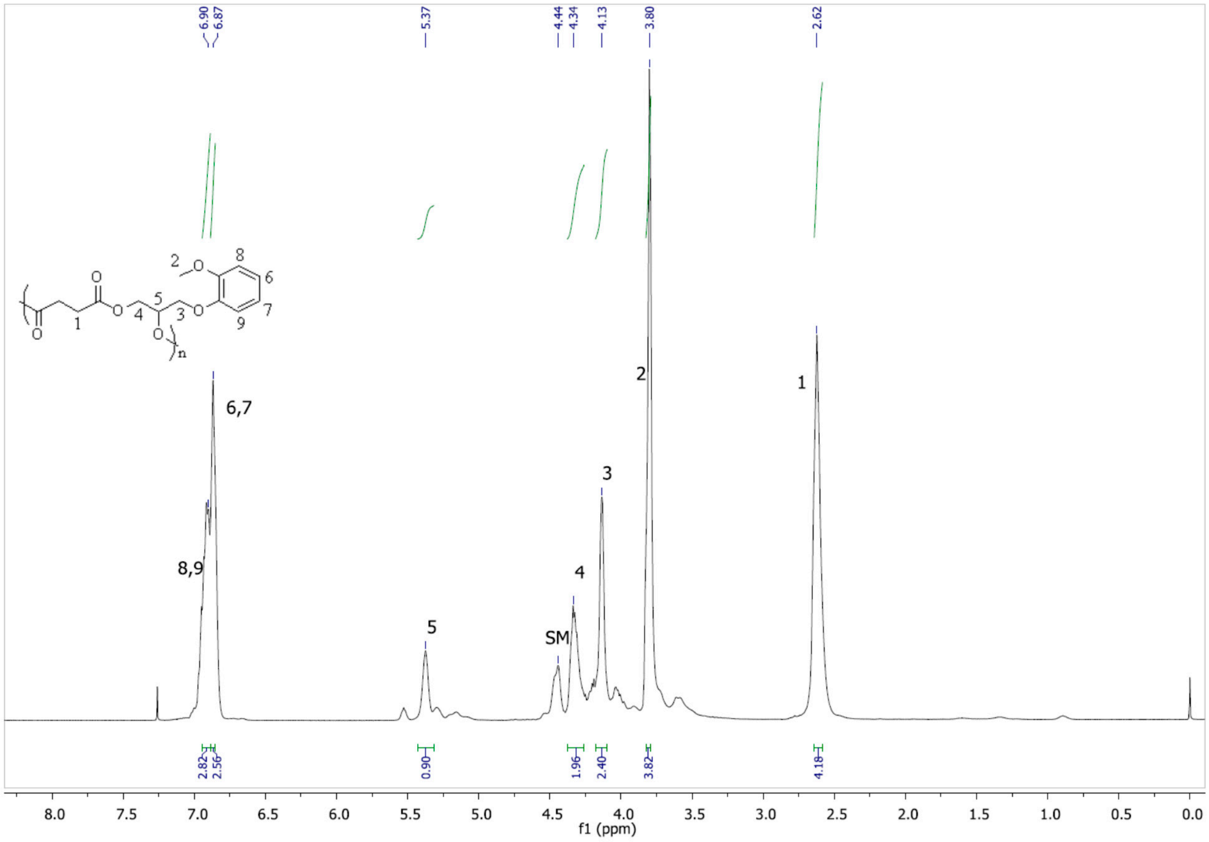

Figure S53: ^{1}H NMR of **PE-1a** using titanium catalyst in $CDCl_3$

^{1}H NMR (400 MHz, $CDCl_3$) δ = 6.90 (m, 2H, H8 and H9), 6.87 (m, 2H, H7 and H8), 5.37 (m, 1H, H6), 4.44 (m, 1H, H5), 4.34 (m, 1H, H4), 4.13 (m, 2H, H3), 3.80 (s, 3H, H2), 2.62 (s, 4H, H1) ppm

References

(1) Truscello, A. M.; Gambarotti, C.; Lauria, M.; Auricchio, S.; Leonardi, G.; Shisodia, S. U.; Citterio, A. One-Pot Synthesis of Aryloxypropanediols from Glycerol: Towards Valuable Chemicals from Renewable Sources. *Green Chem.* **2013**, *15* (3), 625–628. https://doi.org/10.1039/c2gc36793a.

(2) Kosalka, J. J. S. Green Resins from Renewable Resources, Ryerson University, 2014.

(3) Kosalka, J. J. S.; Sacripante, G. G.; Lough, A. J.; Foucher, D. A.; Gossage, R. A. Biomass Utilisation Strategies for Applications in Novel Polymer and Polymer Resin Production. *Polym. From Renew. Resour.* **2017**, *8* (1), 1–10. https://doi.org/10.1177/204124791700800101.

(4) Sayyed, I. A.; Thakur, V. V.; Nikalje, M. D.; Dewkar, G. K.; Kotkar, S. P.; Sudalai, A. Asymmetric Synthesis of Aryloxypropanolamines via OsO4-Catalyzed Asymmetric Dihydroxylation. *Tetrahedron* **2005**, *61* (11), 2831–2838. https://doi.org/10.1016/j.tet.2005.01.074.

(5) Bredikhin, A. A.; Bredikhina, Z. A.; Antonovich, O. A.; Zakharychev, D. V.; Krivolapov, D. B. Crystallization Features and Spontaneous Resolution of 3-(2,6-Dimethoxyphenoxy)Propane-1,2-Diol: The Case of Stable Conglomerate and Metastable Solid Solution. *J. Mol. Struct.* **2017**, *1144*, 443–450. https://doi.org/10.1016/j.molstruc.2017.05.091.
